# Endoxifen downregulates AKT phosphorylation through protein kinase C beta 1 inhibition in ERα+ breast cancer

**DOI:** 10.1038/s41523-023-00606-2

**Published:** 2023-12-19

**Authors:** Swaathi Jayaraman, Xinyan Wu, Krishna R. Kalari, Xiaojia Tang, Mary J. Kuffel, Elizabeth S. Bruinsma, Shahrzad Jalali, Kevin L. Peterson, Cristina Correia, Rachel A. Kudgus, Scott H. Kaufmann, Santosh Renuse, James N. Ingle, Joel M. Reid, Matthew M. Ames, Alan P. Fields, Matthew J. Schellenberg, John R. Hawse, Akhilesh Pandey, Matthew P. Goetz

**Affiliations:** 1 Department of Oncology, Mayo Clinic, Rochester, MN 55905 USA; 2 Department of Laboratory Medicine and Pathology, Mayo Clinic, Rochester, MN 55905 USA; 3 Department of Molecular Pharmacology and Experimental Therapeutics, Mayo Clinic, Rochester, MN 55905 USA; 4 Department of Health Sciences Research, Mayo Clinic, Rochester, MN 55905 USA; 5 Department of Biochemistry and Molecular Biology, Mayo Clinic, Rochester, MN 55905 USA; 6 Department of Cancer Biology, Mayo Clinic Comprehensive Cancer Center, Jacksonville, FL 32224 USA; 7 Department of Cancer Biology, Mayo Clinic, Rochester, MN 55905 USA

**Keywords:** Breast cancer, Breast cancer

## Abstract

Endoxifen, a secondary tamoxifen metabolite, is a potent antiestrogen exhibiting estrogen receptor alpha (ERα) binding at nanomolar concentrations. Phase I/II clinical trials identified clinical activity of Z-endoxifen (ENDX), in endocrine-refractory metastatic breast cancer as well as ERα+ solid tumors, raising the possibility that ENDX may have a second, ERα-independent, mechanism of action. An unbiased mass spectrometry approach revealed that ENDX concentrations achieved clinically with direct ENDX administration (5 µM), but not low concentrations observed during tamoxifen treatment (<0.1 µM), profoundly altered the phosphoproteome of the aromatase expressing MCF7AC1 cells with limited impact on the total proteome. Computational analysis revealed protein kinase C beta (PKCβ) and protein kinase B alpha or AKT1 as potential kinases responsible for mediating ENDX effects on protein phosphorylation. ENDX more potently inhibited PKCβ1 kinase activity compared to other PKC isoforms, and ENDX binding to PKCβ1 was confirmed using Surface Plasma Resonance. Under conditions that activated PKC/AKT signaling, ENDX induced PKCβ1 degradation, attenuated PKCβ1-activated AKT^Ser473^ phosphorylation, diminished AKT substrate phosphorylation, and induced apoptosis. ENDX’s effects on AKT were phenocopied by siRNA-mediated PKCβ1 knockdown or treatment with the pan-AKT inhibitor, MK-2206, while overexpression of constitutively active AKT diminished ENDX-induced apoptosis. These findings, which identify PKCβ1 as an ENDX target, indicate that PKCβ1/ENDX interactions suppress AKT signaling and induce apoptosis in breast cancer.

## Introduction

Estrogen receptor alpha positive (ERα+) breast cancer is the predominant breast cancer subtype, accounting for nearly 70% of all diagnosed cases. For this subtype, endocrine therapies such as the selective estrogen receptor modulator (SERM) tamoxifen (TAM), aromatase inhibitors (AIs) or the selective estrogen receptor degrader ICI-182780 (ICI) (fulvestrant) are standard treatments used alone or in combination with other targeted therapies in the adjuvant and metastatic settings^1–4^. When administered in the adjuvant setting for five years, TAM significantly improves disease-free and overall survival^5^.

Prior studies have demonstrated that endoxifen, a secondary TAM metabolite, is a more potent antiestrogen compared to the parent drug^6–10^, and studies have raised the possibility of additional targets^11^. Preclinical studies revealed superior antitumor activity for Z-endoxifen (ENDX), the most active endoxifen isomer, compared to TAM in both endocrine-sensitive and resistant ERα+ breast cancer models^11^. In a phase I clinical study (NCT01327781), ENDX demonstrated promising antitumor activity and manageable toxicities in endocrine-refractory ERα+ metastatic breast cancer, including *ESR1* mutant tumors, as well as in tumors of non-breast origin that are typically resistant to endocrine therapy^12,13^. In the Phase II randomized clinical trial comparing ENDX with TAM (NCT02311933) in women with ERα+/HER2- metastatic breast cancer who progressed on endocrine therapy, while ENDX did not improve progression free survival (PFS) overall [4.3 m vs 1.8 m; hazard ratio (HR) 0.77, 95% confidence interval (CI) 0.49–1.22)], PFS in CDK4/6 inhibitor (CDK4/6i) naïve patients was prolonged by ENDX compared to TAM (7.2 m vs 2.4 m; HR 0.42, 95% CI 0.22–0.80) (8) supporting the further development of ENDX^14^.

In the ENDX phase I clinical trials, the ENDX peak serum concentrations (C_max_) (measured on Day 28) were reported as 913 ± 142 ng/ml (i.e., 2.44 ± 0.38 µM) at the 80 mg/day dose and 1641 ± 830 ng/ml (i.e., 4.39 ± 2.22 µM) at the 200 mg/day dose^12,13^. Given that endoxifen binds ERα and potently suppresses ERα signaling at nanomolar concentrations^15^, ENDX concentrations > 0.1 µM would be expected to have little additional benefit if ERα were the only pertinent target. To permit better identification of breast cancers that are ENDX sensitive, it is important to identify pathways and proteins impacted by clinically achievable low micromolar ENDX concentrations. Therefore, to identify potential ENDX targets extending beyond ERα signaling, we employed an unbiased mass spectometry approach to analyze the phosphoproteome and total proteome utilizing the aromatase expressing MCF7AC1 cell line model in response to ENDX concentrations achievable in TAM treated patients (0.01 and 0.1 μM) as well as higher concentrations (5 μM) clinically achievable only with direct administration of ENDX. These studies have identified PKCβ1 as a ENDX target whose engagement results in inhibition of AKT signaling and induction of apoptosis.

## Results

### ENDX at 5 μM inhibits growth and induces apoptosis in estrogen deprived ERα+ breast cancer cells

**Fig. 1:**
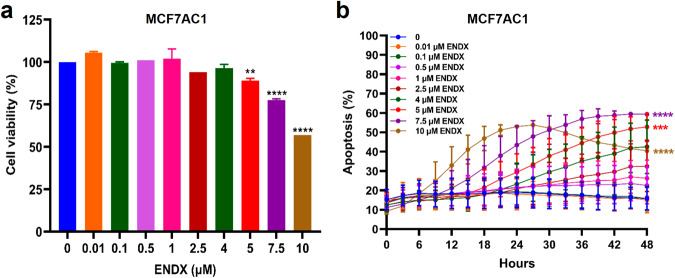
Z-endoxifen (ENDX) effects on cell viability and apoptosis in estrogen deprived ERα+ /HER2- MCF7AC1 cells. **a** Cells grown in CSS medium were treated with vehicle control or the indicated ENDX concentrations for 48 h. Cell viability was assessed by the crystal violet assay. **b** Cells were co-treated with vehicle control or the indicated ENDX concentrations, IncuCyte Annexin V green and NucLight red reagents in CSS medium for 48 h. The apoptosis (%) graphs are presented as the green object count (which correspond to cells that are stained with the IncuCyte green fluorescence Annexin V reagent) divided by the red object count (which correspond to the total number of cells in the culture that are stained with the IncuCyte red fluorescence Nuclight Rapid Red Cell Labeling reagent that labels the nucleus of all cells without perturbing cell function or biology) and displayed as percentage using the IncuCyte S3 analysis software. Data represents the mean of six wells per treatment performed as biological duplicates ± s.d. **p ≤ 0.01; ***p ≤ 0.001; ****p ≤ 0.0001 by one-way ANOVA.

The aromatase expressing endocrine-sensitive ERα+/HER2- MCF7AC1 cell line is a clinically relevant model previously used to predict the clinical efficacy of AIs over TAM, as well as the clinical efficacy of ICI in combination with anastrozole^16,17^. Utilizing this model and a letrozole-resistant derivative, we published that ENDX exhibits superior in vivo antitumor activity compared to TAM and letrozole in both the aromatase inhibitor-sensitive and -resistant models^11^. To build on these observations, utilizing ENDX concentrations ranging from 0 to 10 µM, we evaluated dose-dependent effects of ENDX on cell viability under estrogen deprived conditions, i.e., in medium containing charcoal stripped serum (CSS), to evaluate ENDX effects that may extend beyond ERα inhibition. ENDX concentrations ≥ 2.5 µM significantly reduced cell viability (Fig. 1a) and induced apoptosis in these cells (Fig. 1b). These findings suggest that higher plasma concentrations of ENDX may elicit cytotoxic, and not just cytostatic, effects in ERα+ breast cancer cells.

### ENDX concentration-dependent effects on the phosphoproteome of ERα+ breast cancer cells

**Fig. 2:**
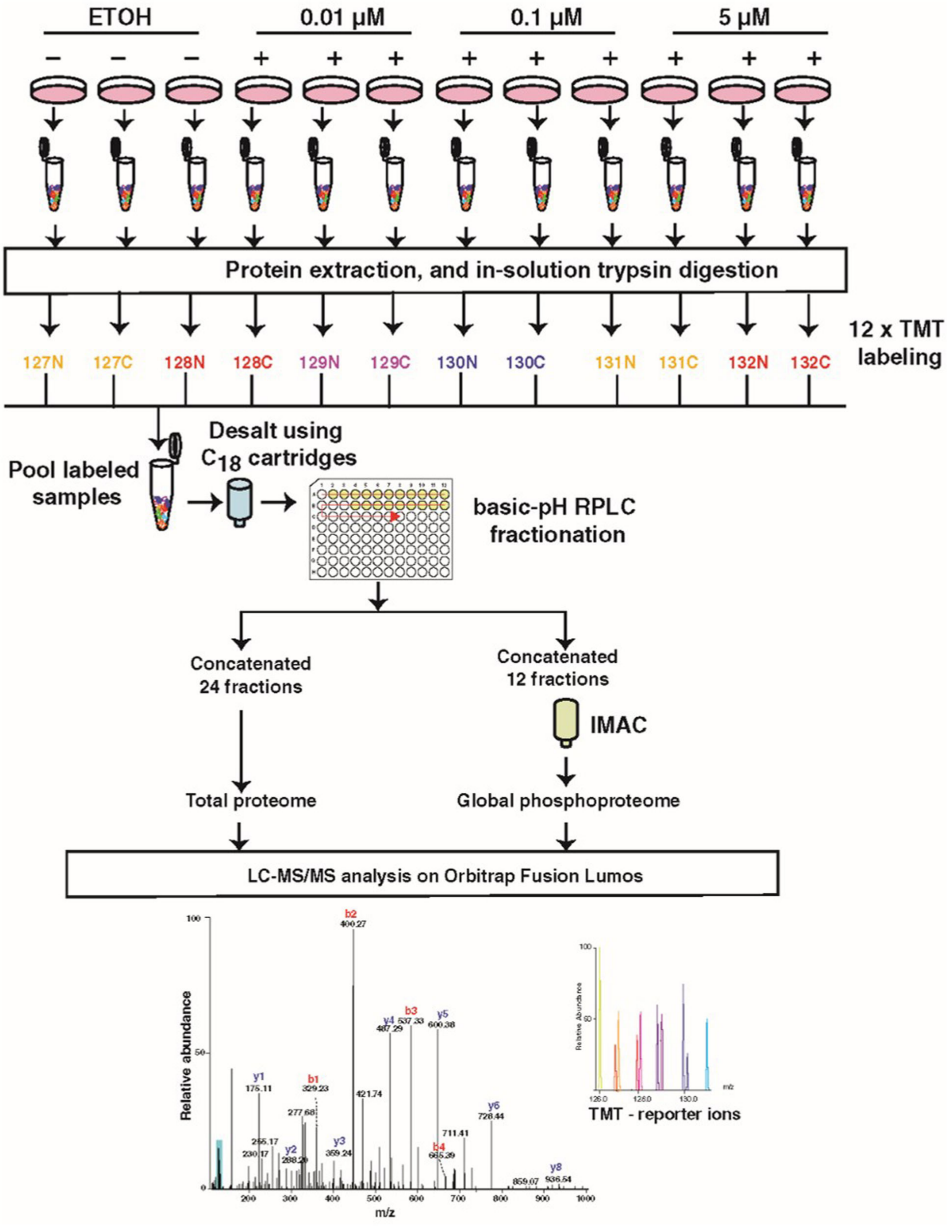
A schematic depicting the strategy used for quantitative proteomic and phosphoproteomic profiling of ENDX-treated MCF7AC1 cells. All experiments were performed in triplicate. Cells maintained in CSS medium were treated with ETOH vehicle control or ENDX at specified dosages for 24 h. Following treatments, cells were harvested and lysed in 8 M urea buffer, followed by trypsin digestion, desalting and TMT labeling. The labeled peptides were fractionated, and phosphopeptides were enriched with immobilized metal affinity chromatography (IMAC) approach. Both fractionated peptides and IMAC-enriched phosphopeptides were analyzed by Orbitrap Lumos mass spectrometer.

We then sought to identify additional protein targets of ENDX that may contribute to its anticancer effects in estrogen deprived conditions. To this end, MCF7AC1 cells were treated with 0.01, 0.1, and 5 µM ENDX concentrations achieved in various clinical settings for 24 h in CSS medium and subjected to TMT labeling-based LC-MS/MS mass spectrometry analysis to evaluate changes in the global protein expression and the phosphoproteome relative to vehicle treated cells (Fig. 2). Assessment of the total proteome identified and quantified 8894 unique proteins (accession number: PXD035007). The impact on global total protein expression induced by ENDX treatment for 24 h in estrogen deprived cells was limited, with only 25, 34, and 65 total proteins differentially altered by treatment with 0.01, 0.1, and 5 µM ENDX, respectively, compared to vehicle treated cells (based on criteria of |1.5|-fold change and *p* value of < 0.05) (Supplementary Table 1). Although ENDX impact on the total proteome was limited, ENDX at 5 µM downregulated two-fold more total proteins compared to 0.1 µM concentration (44 versus 22) and four-fold more total proteins compared to the 0.01 µM concentration (44 versus 11) (Supplementary Fig. 1a). Also, the number of total proteins uniquely downregulated by 5 μM ENDX (35) was greater than the number of total proteins uniquely downregulated by 0.01 μM (3) and 0.1 μM (9) concentrations (Supplementary Fig. 1b).

**Fig. 3:**
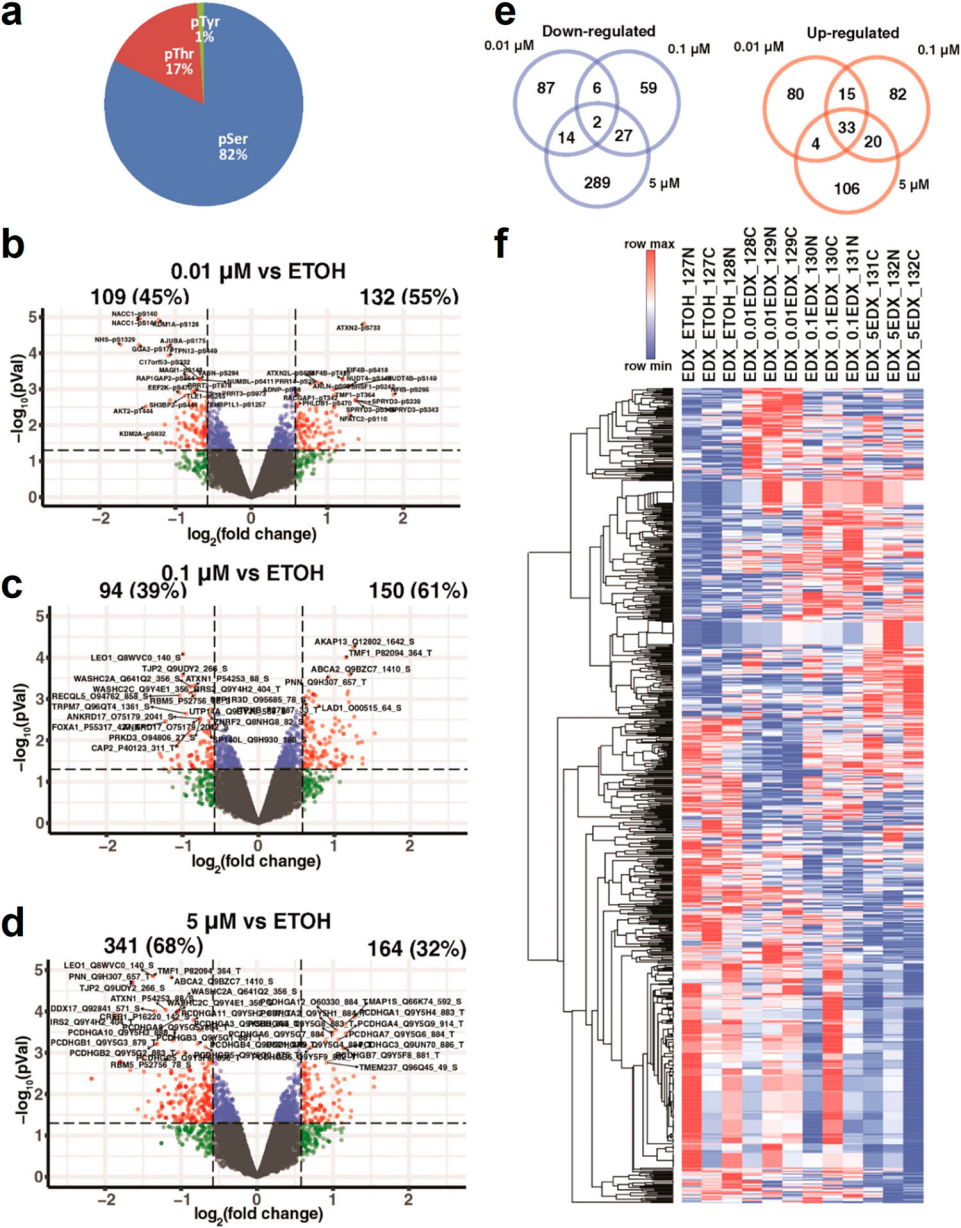
Effects of ENDX on the phosphoproteome of MCF7AC1 cells. **a** A pie chart showing the distribution of identified phosphorylation sites. Volcano plots showing the total number of phosphosites, and the percentage that are upregulated (right side) and downregulated (left side) (Fold change (FC) |1.5|; *p* < 0.05) in cells treated for 24 h in CSS medium with 0.01 (**b**), 0.1 (**c**) or 5 μM (**d**) ENDX relative to vehicle treated cells, as detected by mass spectrometry analysis. **e** Venn diagram indicating the overlap of upregulated (pink) and downregulated (blue) phosphosites in ENDX-treated cells relative to vehicle treated cells. **f** Heatmap indicating relative abundance of the phosphosites analyzed in the ENDX-treated cells relative to vehicle treated cells. The hierarchical clustering of phosphosites is shown on the left.

In contrast, ENDX displayed a much greater impact on the phosphoproteome compared to the total proteome during the course of the 24-h treatment. Phosphoproteomic analyses identified 14,715 unique phosphosites derived from 4480 proteins (accession number: PXD035007). Out of all sites, 10,046 (82%) are phospho-Serine (pS) sites, 2042 (17%) are phospho-Threonine (pT) sites, and 134 (1%) phospho-Tyrosine sites, as shown in Fig. 3a. In MCF7AC1 cells, treatment with 0.01 µM ENDX resulted in the downregulation of 109 phosphosites and upregulation of 132 phosphosites (|1.5|-fold change and a *p* value of < 0.05) (Fig. 3b). Similarly, treatment with 0.1 µM ENDX led to the downregulation of 94 phosphosites and the upregulation of 150 phosphosites (Fig. 3c). Finally, 5 µM ENDX treatment downregulated 341 phosphosites and upregulated 164 phosphosites (Fig. 3d). Remarkably, ENDX at 5 µM downregulated three-fold more phosphosites compared to 0.1 µM (341 versus 94) and 0.01 µM (341 versus 109, Fig. 3b–d). In addition, the number of phosphosites uniquely downregulated by 5 µM ENDX (289) was also three-fold greater than the number uniquely downregulated by 0.01 µM (87) and 0.1 µM (59, Fig. 3e). Heat-map analysis suggested an ENDX concentration-dependent downregulation for a subset of these phosphosites (Fig. 3f). A comparison of the phosphosite list with the total protein list altered by ENDX revealed minimal overlap (Supplementary Fig. 1c).

**Fig. 4:**
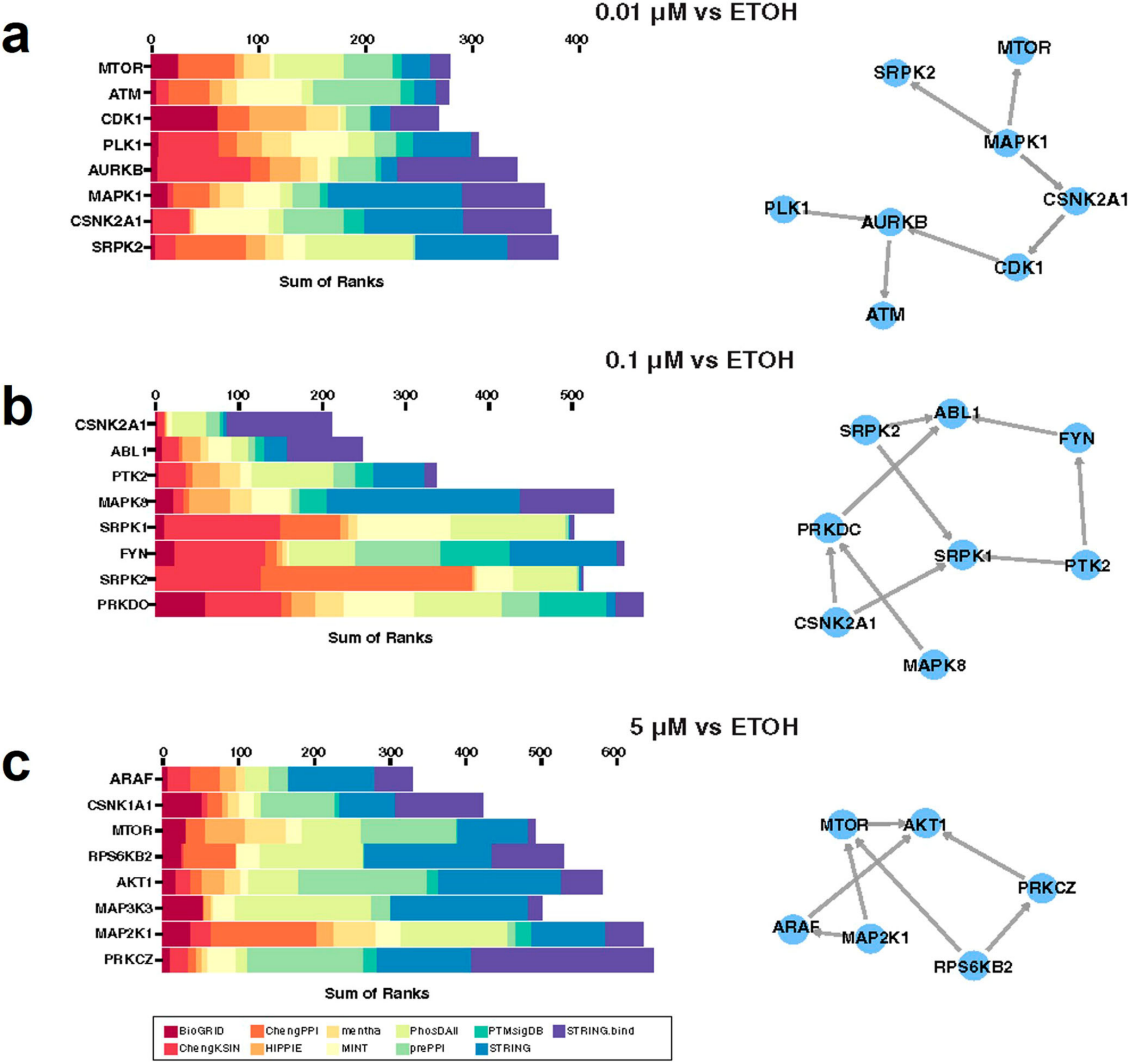
Kinase enrichment analysis predicts AKT signaling is regulated by high-dose ENDX. 210 phospho-regulated proteins from 0.01 μM (**a**), 224 phospho-regulated proteins from 0.1 μM (**b**), and 347 phospho-regulated proteins from 5 μM (**c**) ENDX treated cells were respectively used for KEA3 upstream kinase analysis. Left panels: Integrated rankings of most enriched kinases across libraries based on the MeanRank. The stacking bar chart shows the summation of the MeanRank derived from the libraries used for the KEA analysis. The libraries are color-coded. Right panels: Kinase co-regulatory networks constructed from kinase-kinase interactions between top-ranked kinase results for the integrated rankings. Directed edges indicate interactions supported by kinase-substrate evidence.

In order to identify protein phosphorylation signaling pathways regulated by ENDX, we performed a kinase enrichment analysis^18^, which integrated multiple databases covering kinase-substrate interactions, kinase-protein interactions, and interactions supported by co-expression and co-occurrence data to infer the overrepresentation of upstream kinases whose putative substrates are among the phosphorylated proteins altered by ENDX treatments. We performed three separate analyses for 210 proteins, 224 proteins, and 347 proteins differentially phosphorylated in cells treated with 0.01 µM, 0.1 µM, and 5 µM ENDX, respectively. Figure 4 shows the top enriched upstream kinases predicted to regulate the protein phosphorylation changes induced by ENDX at different concentrations. Casein kinase (CSNK1A1), Serine/arginine-rich protein-specific kinase (SRPK1, SRPK2), and Mitogen-activated protein kinases (MAPK1 and MAPK8) were identified as putative kinases regulated by low-dose (0.01 and 0.1 µM) ENDX (Fig. 4a, b). This analysis suggested that proteins (MTOR, RPS6K, and AKT1) involved in the AKT signaling pathway could potentially be regulated by high-dose (5 µM) ENDX (Fig. 4c).

**Fig. 5:**
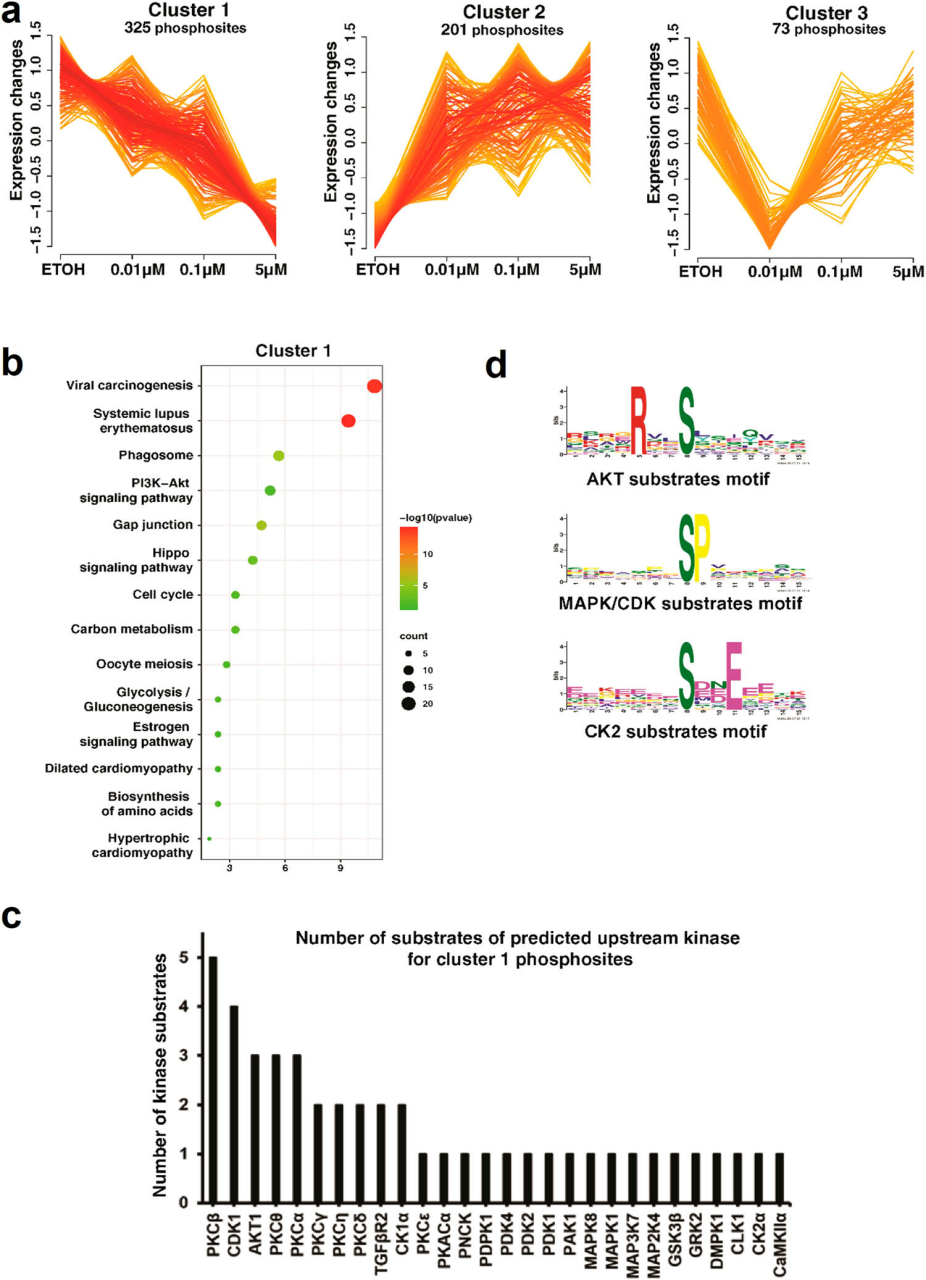
Downregulated phosphosites following ENDX treatment are enriched for PKCβ, CDK1, and AKT target sequences. **a** Fuzzy c-means clustering showing the classification of ENDX treatment effects on the phosphoproteome into three regulatory clusters. Cluster 1 represents phosphosites downregulated by ENDX in a dose-dependent manner. Cluster 2 represents phosphosites that are upregulated by ENDX at all concentrations examined. Cluster 3 represents phosphosites downregulated at 0.01 μM concentration but mostly unaffected at the 0.1 and 5 μM concentrations. **b** Molecular and cellular pathways potentially impacted by ENDX. Kyoto Encyclopedia of Genes and Genomes (KEGG) database analysis of the phosphosites altered by ENDX in cluster 1 showing the top biological pathways associated with these phosphosites. **c** Enriched phosphorylation motifs identified in cluster 1 phosphosites. **d** Graph showing the frequency of kinases known to phosphorylate cluster 1 phosphosites that were depleted by ENDX as assessed using NetworKIN and RoKAI prediction tools.

In addition to recognizing potential upstream kinases based on the effects of ENDX on phosphorylation, we also performed fuzzy-C mean clustering to identify the regulation patterns induced by different ENDX concentrations. This analysis identified three clusters depicting three different regulatory patterns (Fig. 5a). Cluster 1 represents 325 phosphosites downregulated by ENDX in a dose-dependent manner, cluster 2 represents 201 phosphosites upregulated by ENDX at all concentrations and cluster 3 represents 73 phosphosites downregulated at 0.01 μM concentration but mostly unaffected at the 0.1 and 5 μM concentrations (Supplementary Table 2).

Given our interest in studying ENDX dose-dependent effects and the mechanistic basis for induction of apoptosis at 5 µM ENDX, we focused on cluster 1. KEGG pathway enrichment analysis using DAVID, an online gene functional annotation tool^19,20^, identified viral carcinogenesis, systemic lupus erythematosus, phagosome, PI3K-AKT signaling pathway, and gap junction as the top five biological pathways impacted by ENDX (Fig. 5b).

### ENDX downregulated phosphosites are enriched for PKCβ, CDK1, and AKT1 target sequences

We postulated that the observed ENDX effects on cluster 1 phosphosites are due to effects on kinase mediators of these phosphorylation events. To identify these kinases, we used NetworKIN and RoKAI kinase prediction tools^21,22^. Using the 325 phosphosites from cluster 1 as an input, these two tools collectively identified protein kinase C beta (PKCβ) and cyclin-dependent kinase 1 (CDK1) followed by AKT1 and PKCθ/PKCα as the top five most frequently predicted kinases involved (Fig. 5c; Supplementary Table 3). Further, motif enrichment analysis identified that RXXpS, pS/pTP and pSXXE as prevalent motifs in the regulated cluster 1 phosphosites. These motifs can be mapped to the AKT, MAPK/CDK and CK2 kinase-substrate motifs, respectively (Fig. 5d).

### ENDX at 5 µM downregulates AKT^Ser473^ phosphorylation in ERα+ breast cancer cells

**Fig. 6:**
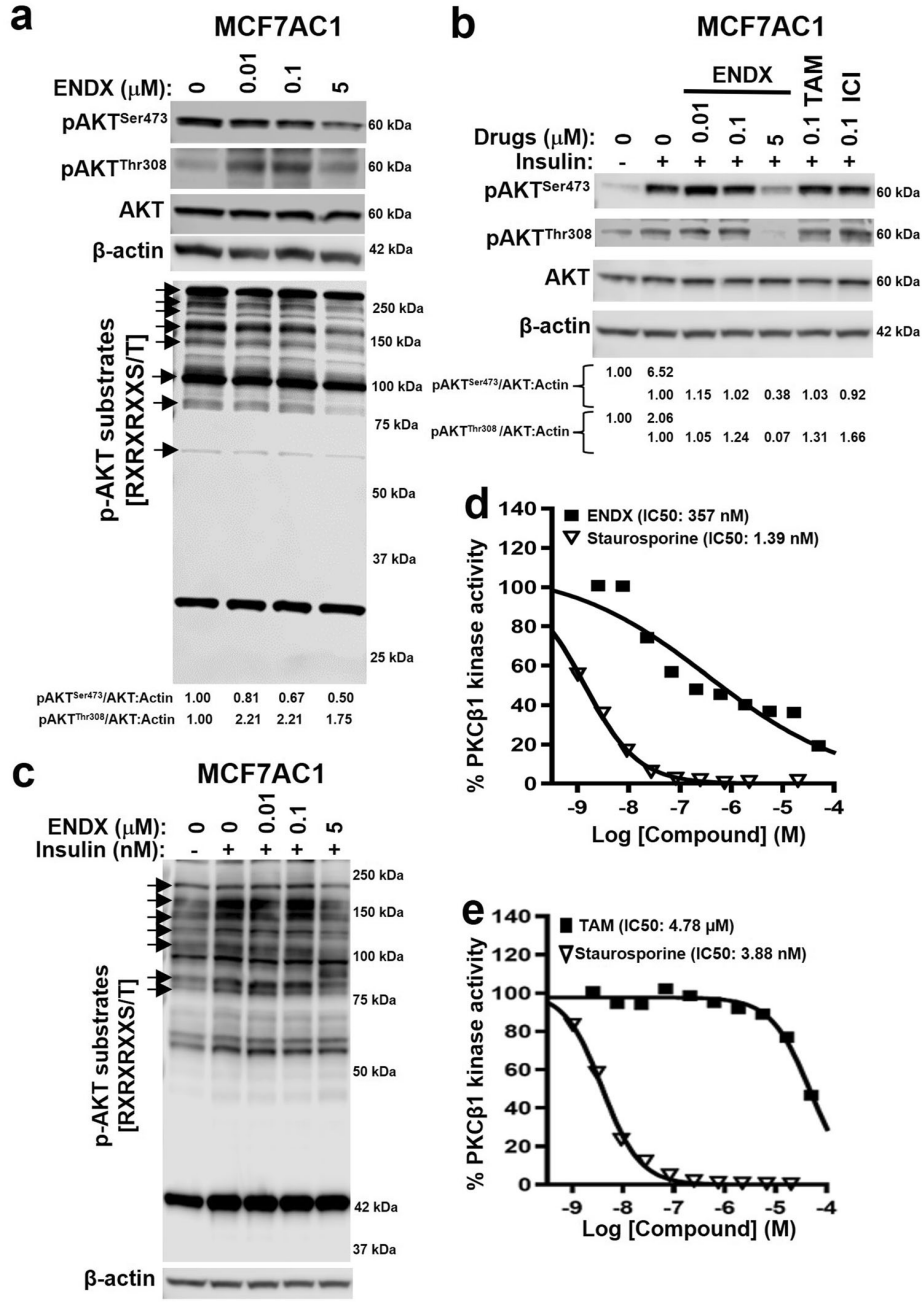
ENDX specifically downregulates pAKT^Ser473^ at 5 µM and inhibits PKCβ1 kinase activity. **a** MCF7AC1 cells in CSS medium were treated for 24 h with vehicle control or 0.01, 0.1, and 5 µM ENDX. Immunoblot assays of pAKT^Ser473^, pAKT^Thr308^, AKT and p-AKT substrates are shown with β-actin as a loading control. **b** Serum starved MCF7AC1 cells were pretreated with vehicle control, 0.01, 0.1, 5 µM ENDX, 0.1 µM tamoxifen (TAM) or 0.1 µM ICI-182780 (ICI) followed by the addition of 100 nM insulin for 1 h as indicated. IB assays of pAKT^Ser473^, pAKT^Thr308^, AKT and β-actin are shown. **c** Serum starved MCF7AC1 cells were pretreated with vehicle control and 0.01, 0.1, and 5 µM ENDX for 2 h followed by the addition of 100 nM insulin for 1 h as indicated. IB assay of pAKT substrates and β-actin are shown. **d**, **e** In vitro kinase assay showing % PKCβ1 kinase activity in the presence of different concentrations of ENDX and TAM. The broad-spectrum kinase inhibitor staurosporine serves as a positive control. The IC_50_ concentration of ENDX, TAM, and staurosporine are indicated.

We previously showed that ENDX, but not TAM, attenuates AKT phosphorylation at Ser473, a modification required for full AKT activation, both in vitro and in vivo in a letrozole-resistant MCF7AC1 tumor model^11^. Our pathway analysis studies identified PI3K-AKT signaling as one of the top biological pathways targeted by ENDX (Fig. 5b), with AKT1 frequently predicted as a top kinase for phosphosites downregulated by ENDX (Fig. 5c). Therefore, we examined the effect of ENDX on AKT^Ser473^ phosphorylation in estrogen deprived MCF7AC1 cells. Immunoblot assays revealed that ENDX at 5 µM, but not at 0.01 and 0.1 µM, attenuated AKT^Ser473^ phosphorylation compared to vehicle treated cells (Fig. 6a). Given that phosphorylation of AKT is initiated at Thr308 followed by phosphorylation at Ser473 for full AKT activation^23^, we also evaluated ENDX effects on AKT^Thr308^ phosphorylation. While 0.01 and 0.1 µM concentrations of ENDX stimulated AKT^Thr308^ phosphorylation, ENDX at 5 µM did not alter AKT^Thr308^ phosphorylation under estrogen deprived conditions (Fig. 6a). Because commercially available phosphosite specific antibodies for predicted AKT-mediated phosphorylations in cluster 1 were limited, we turned to an alternate approach where we evaluated ENDX effects on the levels of total phospho-AKT substrates. We used a commercially available antibody that specifically recognizes the RXXpS/pT AKT motif in AKT substrates to determine whether downregulation of AKT^Ser473^ phosphorylation by 5 μM ENDX has a global impact on the phosphorylation of AKT substrates. Consistent with reduced AKT^Ser473^ phosphorylation, ENDX at 5 μM, but not 0.01 and 0.1 μM, also reduced the phosphorylation of AKT substrates compared to vehicle treated estrogen deprived MCF7AC1 cells (Fig. 6a; arrows). We next evaluated ENDX effects on AKT^Ser473^ and AKT^Thr308^ phosphorylations in the endocrine-sensitive MCF7AC1 xenograft model in vivo, where ENDX exhibited superior antitumor activity compared to letrozole and TAM^11^. Consistent with the in vitro findings, treatment of mice harboring MCF7AC1 tumors with high-dose ENDX (75 mg/kg) but not low-dose ENDX (25 mg/kg), TAM or letrozole, attenuated AKT^Ser473^ phosphorylation and reduced the levels of AKT phosphorylated substrates but had no appreciable impact on AKT^Thr308^ phosphorylation compared to control treatment (Supplementary Fig. 2a). Of note, previously published murine PK studies demonstrated that in ENDX treated mice receiving a single dose of 25 mg/kg or 75 mg/kg, plasma C_max_ values were 103 ± 97 ng/ml (0.28 ± 0.26 µM) and 660 ± 511 ng/ml (1.77 ± 1.37 µM), respectively^24^. A follow-up study demonstrated that in mice treated with 25 and 100 mg/kg doses for five consecutive days, ENDX plasma concentrations were 73.1 ± 16.8 ng/ml (0.20 ± 0.04 µM) and 1700 ± 60 ng/ml (4.55 ± 0.16 µM), respectively^24^. Thus, these data support a dose and concentration-dependent effect of ENDX on AKT signaling both in vitro and in vivo.

Next, we asked whether ENDX can block ligand stimulated AKT^Ser473^ phosphorylation. Insulin, a known activator of AKT^Ser473^ phosphorylation^25^, robustly stimulated AKT^Ser473^ phosphorylation in serum starved MCF7AC1 cells. Pretreatment with ENDX at 5 μM, but not at 0.01 and 0.1 μM, for two hours prior to insulin stimulation blocked AKT^Ser473^ phosphorylation (Fig. 6b). Insulin treatment also stimulated AKT^Thr308^ phosphorylation, albeit modestly compared to AKT^Ser473^ phosphorylation in serum starved MCF7AC1 cells (Fig. 6b). Interestingly, pretreatment with ENDX at 5 μM was also able to block AKT^Thr308^ phosphorylation (Fig. 6b). Additionally, ENDX at 5 µM also diminished insulin-stimulated phosphorylation of AKT substrates (Fig. 6c). In contrast, treatment with clinically attainable concentrations of 0.1 µM TAM or 0.1 µM ICI, failed to inhibit insulin-stimulated AKT^Ser473^ and AKT^Thr308^ phosphorylations (Fig. 6b), an effect also observed with ERα-targeting 0.1 µM concentration of ENDX. While ENDX is equipotent with 4-HT^26^, 4-HT was not included as a comparator in this study, as clinically attainable serum concentrations of 4-HT reported in ERα+ breast cancer patients receiving 20 mg/day TAM monotherapy is <5 nM^27,28^. The ability of ENDX at 5 µM, but not at 0.01 or 0.10 µM, to block insulin-stimulated AKT^Ser473^ phosphorylation was also observed in the ERα+/HER2- T47D breast cancer cells under serum starved conditions, with TAM and ICI again failing to block insulin-stimulated AKT^Ser473^ phosphorylation (Supplementary Fig. 2b). However, contrary to the observation in serum starved MCF7AC1 cells (Fig. 6b), insulin induced stimulation of AKT^Thr308^ phosphorylation was not blocked by ENDX at 5 µM in serum starved T47D cells (Supplementary Fig. 2b). Collectively, these findings suggest that ENDX attenuates AKT signaling primarily through attenuation of AKT^Ser473^ phosphorylation in ERα+ breast cancer cells at clinically relevant 5 µM concentration, a unique effect not observed with other SERM’s at clinically relevant concentrations.

### ENDX inhibits PKCβ1 kinase activity and binds to PKCβ1

Based on a report that PKCβ1 phosphorylates AKT at Ser473^29^, we postulated that ENDX might mediate its effects on AKT through PKCβ1. To address this possibility, we first sought to evaluate ENDX effects on PKCβ1 kinase activity. To this end, we evaluated the concentration-dependent effects of ENDX on a kinase panel composed of 12 PKC isoforms, since multiple PKC family members including PKCβ were identified in the kinase prediction analysis (Fig. 5c). While ENDX inhibited the kinase activity of PKCβ1 with an IC_50_ concentration of 360 nM (Fig. 6d), ENDX did not inhibit other PKC family members as potently (Supplementary Table 4). TAM, a known PKC inhibitor^30^, also inhibited PKCβ1 kinase activity, but at higher concentrations (IC_50_ = 4.9 μM) (Fig. 6e), a concentration not achievable with the 20 mg/day dose. These findings suggest that ENDX may inhibit PKCβ1 kinase activity in vitro.

We next determined whether ENDX directly bound PKCβ1. By employing surface plasmon resonance (SPR), a widely used method for assessing protein-ligand interactions and using a wide range of ENDX concentrations (100–8000 nM), we demonstrate that ENDX binds PKCβ1 (Supplementary Fig. 3a, b). However, given that ENDX binding did not reach saturation levels over the concentration range studied (Supplementary Fig. 3c, d), an accurate K_D_ for PKCβ1 binding could not be established. Taken together, these findings establish PKCβ1 as a potential ENDX substrate.

### ENDX downregulates AKT^Ser473^ phosphorylation through PKCβ1 inhibition in ERα+ breast cancer cells

**Fig. 7:**
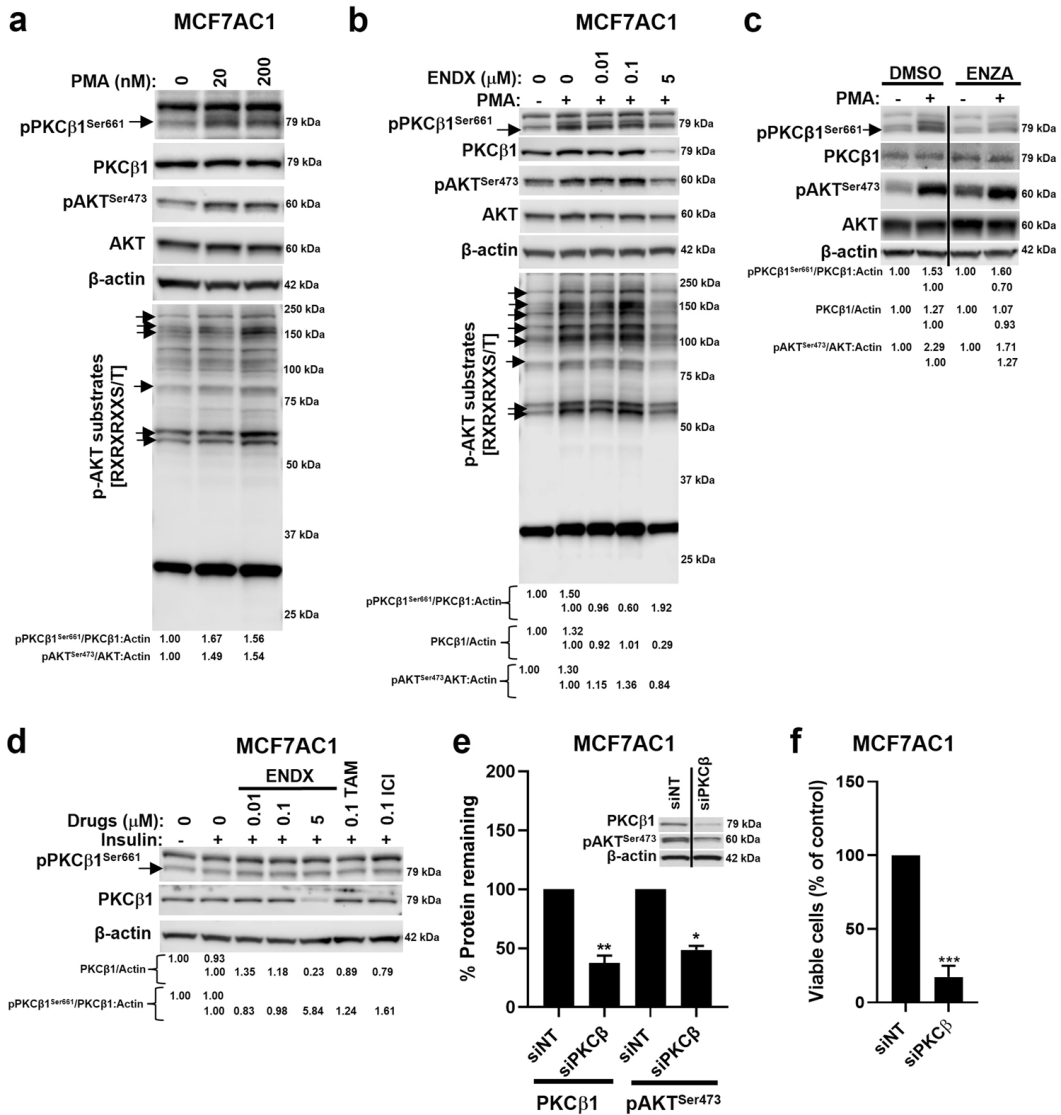
Role of PKCβ1 in mediating ENDX inhibition of AKT^Ser473^ phosphorylation. **a** Serum starved MCF7AC1 cells were treated with vehicle control or 20 and 200 nM PMA for 20 min. IB assays of pPKCβ1^Ser661^, PKCβ1, pAKT^Ser473^, AKT, p-AKT substrates and β-actin are shown. **b** Serum starved MCF7AC1 cells were pretreated with vehicle control or 0.01, 0.1, and 5 µM ENDX for 2 h followed by the addition of 200 nM PMA for 20 min as indicated. IB assays of pPKCβ1^Ser661^, PKCβ1, pAKT^Ser473^, AKT, p-AKT substrates and β-actin are shown. **c** Serum starved MCF7AC1 cells were pretreated with vehicle control or 1 µM ENZA for 2 h followed by the addition of 200 nM PMA for 30 min as indicated. IB assays of pPKCβ1^Ser661^, PKCβ1, pAKT^Ser473^, AKT and β-actin are shown. **d** Serum starved MCF7AC1 cells were pretreated with vehicle control, 0.01, 0.1, and 5 µM ENDX, 0.1 µM TAM or 0.1 µM ICI followed by the addition of 100 nM insulin for 1 h as indicated. IB assays of pPKCβ1^Ser661^, PKCβ1 and β-actin are shown. **e** MCF7AC1 cells in CSS medium were transfected with non-targeting (siNT) or PKCβ-targeting (siPKCβ) siRNAs for 48 h. IB assays of PKCβ1, pAKT^Ser473^ and β-actin are shown. The histogram indicates the percentage (%) of PKCβ1 and pAKT^Ser473^ protein levels remaining upon PKCβ1 knockdown in siPKCβ-treated cells relative to siNT-treated cells from two biological replicates ± s.d. The vertical lines indicate that different lanes of the same blot were juxtaposed to remove intervening lanes. **f** MCF7AC1 cells were treated with siNT or siPKCβ1 in CSS medium for 6 days. Cell viability was assessed by crystal violet assays. Data represents the mean of six wells per treatment performed as biological triplicates ± s.d. *p ≤ 0.05; **p ≤ 0.01; ***p < 0.001 by one sample *t*-test.

To determine the role of PKCβ1 in mediating ENDX effects on AKT^Ser473^ phosphorylation, we first assessed whether PKCβ1 activation impacts AKT^Ser473^ phosphorylation. In serum starved MCF7AC1 cells, the PKC agonist phorbol myristyl acetate (PMA) stimulated PKCβ1^Ser661^ auto-phosphorylation and AKT^Ser473^ phosphorylation, which was associated with increased levels of AKT substrate phosphorylation (Fig. 7a). We next evaluated the effects of ENDX on PKCβ1 under PMA-stimulated conditions. Pretreatment with ENDX at 0.01, 0.1, and 5 μM had either no (0.01, 0.1 μM) or minimal (5 μM) effects to block PMA-stimulated PKCβ1^Ser661^ phosphorylation respectively. In contrast, only ENDX 5 μM robustly reduced PKCβ1 total protein levels, which correlated with reduced AKT^Ser473^ phosphorylation and AKT substrate phosphorylation (Fig. 7b). In contrast, while treatment with the potent and selective ATP competitive PKCβ kinase inhibitor enzastaurin reduced PKCβ1^Ser661^ phosphorylation, it neither impacted the expression of PKCβ1 nor downregulated AKT^Ser473^ phosphorylation (Fig. 7c). Further, in insulin treated MCF7AC1 cells, ENDX pretreatment also reduced PKCβ1 total protein levels, with no effects on PKCβ1^Ser661^ phosphorylation. In contrast, both TAM and ICI pretreatments failed to diminish PKCβ1 total protein expression in insulin-treated MCF7AC1 cells (Fig. 7d), which correlated with the lack of attenuation of AKT^Ser473^ phosphorylation (Fig. 6b).

Given that ENDX robustly blocked PMA- and insulin-stimulated AKT^Ser473^ phosphorylation and additionally targeted PKCβ1 for degradation (Fig. 6b, Fig. 7b), we sought to determine the effects of downregulating PKCβ1 protein expression on AKT^Ser473^ phosphorylation using three different approaches to silence PKCβ1 expression in MCF7AC1 cells. In the first approach, we used a commercially available siRNA that targets an mRNA sequence common to both PKCβ1 and PKCβ2 isoforms (siPKCβ) (Fig. 7e). In the second, we used a custom-designed siRNA from Dharmacon that specifically targets nucleotides 2049–2067 of the PKCβ1 mRNA, a target sequence that is unique and distinct from PKCβ2 (Supplementary Fig. 4a). In the third approach, we utilized a doxycycline (dox)-inducible SMART vector inducible human PRKCB mCMV-TurboGFP shRNA for PRKCB gene silencing (shPKCβ1^dox^) (Supplementary Fig. 4b). Of these approaches, siPKCβ resulted in the greatest reduction in PKCβ1 protein levels (Fig. 7e). Accordingly, we sought to assess the biological effects of PKCβ1 knockdown using siPKCβ. Even though PKCβ2 is theoretically targeted by this reagent, PKCβ2 protein expression is undetectable in MCF7AC1 cells (Supplementary Fig. 4c), indicating that effects on expression of PKCβ2 are unlikely to contribute to the effects of siPKCβ. Reduction in PKCβ1 protein expression by siPKCβ resulted in a 51% reduction in AKT^Ser473^ phosphorylation levels compared to non-targeting (siNT) control at 48 h (Fig. 7e). Importantly, PKCβ siRNA did not affect the expression of other PKC family members (Supplementary Fig. 4d), suggesting that the decrease in AKT^Ser473^ phosphorylation was due to PKCβ1 alone. Downregulation of PKCβ1 protein levels also significantly inhibited the growth of MCF7AC1 cells (Fig. 7f). Thus, PKCβ siRNA recapitulated both the signaling and growth inhibitory effects of ENDX.

To determine whether the observed effects of ENDX on PKCβ1 degradation and AKT^Ser473^ phosphorylation inhibition are dependent on the presence of ERα, we additionally evaluated ENDX effects on PKCβ1 degradation and AKT^Ser473^ phosphorylation in the ER negative (ER-) MDAMB231 breast cancer cells and nonbreast HEK293F cells, a human embryonic kidney cell line, both of which express higher amounts of PKCβ1 compared to ERα+ MCF7AC1 cells (Supplementary Fig. 5a). Pretreatment with 5 µM ENDX for 2 h followed by treatment with 100 nM insulin for 1 h did not impact PKCβ1 protein levels in either cell line (Supplementary Fig. 5b). While insulin treatment induced AKT^Ser473^ phosphorylation in ER- cells, pretreatment with ENDX did not inhibit this phosphorylation (Supplementary Fig. 5b). To determine whether the addition of ERα to the MDAMB231 cell line could facilitate ENDX effects on PKCβ1, we performed doxycycline (dox)-induced ERα protein expression in MDAMB231 cells and evaluated ENDX effects on PKCβ1 using the above-mentioned experimental conditions. While forced expression of ERα in dox-induced cells modestly decreased PKCβ1 protein levels compared to nondox-induced cells, ENDX pretreatment displayed no impact on PKCβ1 protein expression in the presence of ERα (Supplementary Fig. 5c). ERα overexpression also resulted in increased AKT^Ser473^ phosphorylation that remained unaffected by ENDX pretreatment in these cells. Additionally, we pretreated MDAMB231 and HEK293F cells with or without 5 µM ENDX for 1 h followed by 0 or 200 nM PMA treatment for 20 min and evaluated effects on PKCβ1. Treatment with PMA had minimal effects on PKCβ1 phosphorylation and ENDX pretreatment in the presence of PMA displayed no impact on PKCβ1 protein expression (Supplementary Fig. 5d). Taken together, these data demonstrate that in ER- cells, while AKT signaling may be further activated by insulin, PMA does not result in meaningful activation of PKCβ1, suggesting that activation of AKT signaling in ER- cells may not be mediated through PKCβ1 nor blocked by ENDX.

### ENDX at 5 μM replicates apoptotic effects of the pan-AKT inhibitor MK-2206 in estrogen deprived ERα+ breast cancer cells

**Fig. 8:**
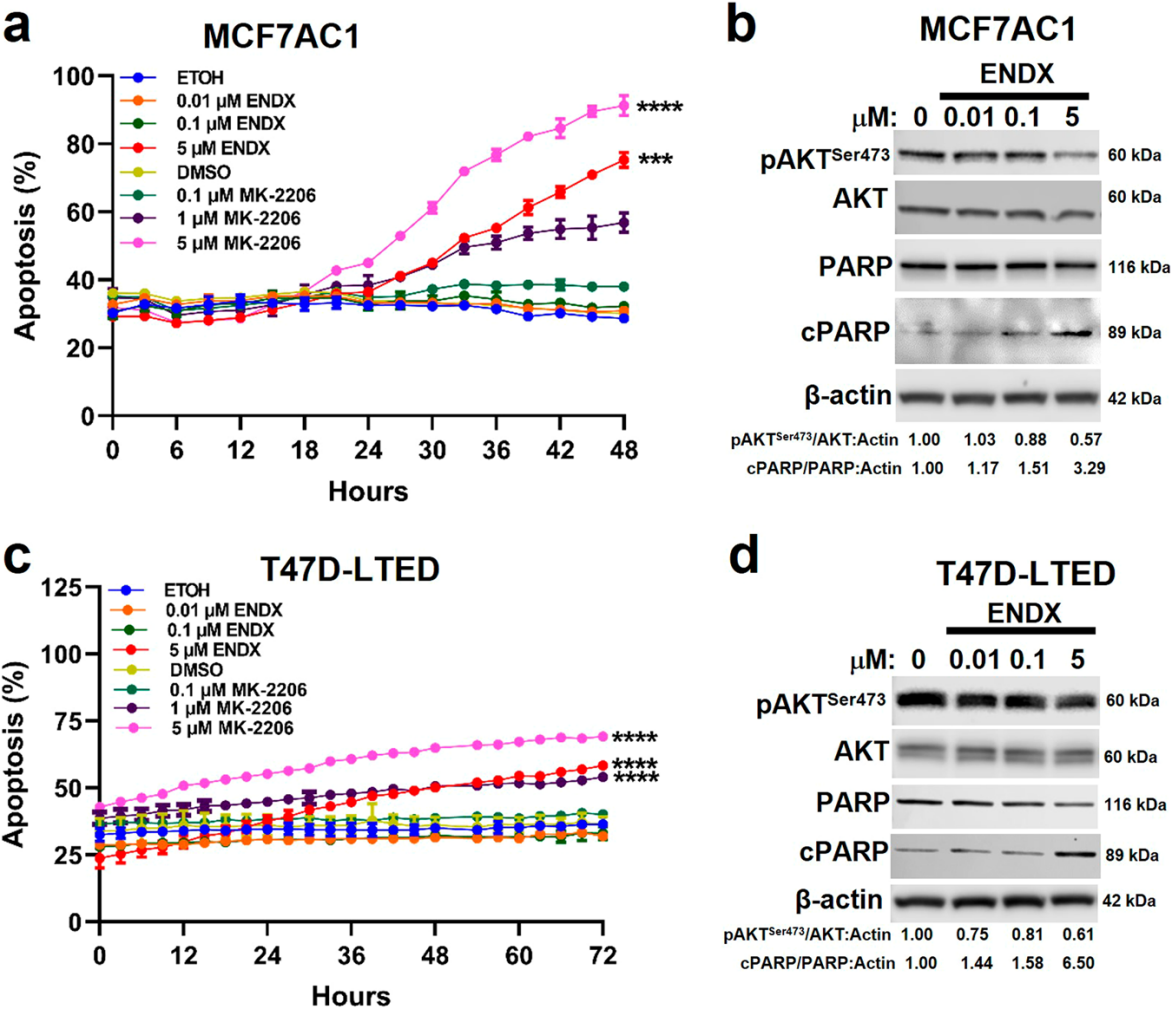
ENDX replicates the effects of the pan-AKT inhibitor, MK-2206, on apoptosis. MCF7AC1 (**a**) and T47D-LTED (**c**) cells were co-treated with vehicle control or 0.01, 0.1, and 5 µM ENDX or MK-2206 in the presence of IncuCyte Annexin V green and NucLight red reagents in CSS medium for 48 h. The apoptosis (%) graph was generated as described in Fig. 1b. Data represents the mean of six wells per treatment performed as biological duplicates ± s.d. ***p ≤ 0.001; ****p < 0.0001 by one-way ANOVA. MCF7AC1 (**b**) and T47D-LTED (**d**) cells in CSS medium were treated with vehicle control or 0.01, 0.1 and 5 µM ENDX for 24 h. IB assay of pAKT^Ser473^, AKT, PARP, cleaved PARP, and β-actin are shown.

AKT activation promotes cell survival by blocking apoptosis^31–33^. Conversely, inhibition of AKT signaling with the allosteric pan-AKT inhibitor MK-2206 induces apoptosis in malignant cells^34–36^. To assess the impact of AKT inhibition on estrogen deprived MCF7AC1 cells, MK-2206 was administered at a variety of concentrations. These studies showed that growth was inhibited at MK-2206 concentrations ≥ 0.1 µM, AKT^Ser473^ phosphorylation was reduced at MK-2206 concentrations ≥ 1 µM, and apoptosis as manifested by both annexin V binding and caspase-mediated PARP1 cleavage was induced at 5 µM MK-2206 (Fig. 8a, Supplementary Fig. 6a–c). In these same cells, treatment with ENDX at 5 µM induced apoptosis (Fig. 8a). We went on to evaluate low (0.01 and 0.1 µM) and higher (5 µM) ENDX concentrations and demonstrated that only the 5 µM concentrations reduced AKT^Ser473^ phosphorylation and increased PARP cleavage (Fig. 8b). Consistent with these in vitro finding, in the MCF7AC1 xenograft model, in vivo treatment with ENDX at 75 mg/kg but not at 25 mg/kg also increased PARP cleavage (Supplementary Fig. 6d).

We next asked whether ENDX had similar effects on T47D cells. Unlike MCF7AC1 cells, parental T47D cells failed to proliferate in CSS medium (Supplementary Fig. 6e). Therefore, we examined ENDX effects on the growth of the ERα+/HER2- long-term estrogen-deprived (LTED) T47D cell line model (T47D-LTED) that proliferates well in CSS medium. Evaluation of basal protein expression revealed reduced ERα levels and a modest decrease in AKT^Ser473^ phosphorylation in the T47D-LTED cell line compared to the parental T47D cell line (Supplementary Fig. 6f). As noted with MCF7AC1 cells, treatment of T47D-LTED cells with MK-2206 significantly inhibited growth starting at 0.1 µM (Supplementary Fig. 6g). Further, MK-2206 at 1 and 5 µM, but not 0.1 µM, also reduced AKT^Ser473^ phosphorylation and increased apoptosis, as indicated by both annexin V staining and increased PARP cleavage (Supplementary Fig. 6h, i), phenocopying the reported biological effects of MK-2206^37,38^. Treatment of T47D-LTED cells with 5 µM ENDX likewise attenuated AKT^Ser473^ phosphorylation, inhibited growth, and induced apoptosis as manifested by annexin V binding and PARP1 cleavage (Figs. 8c, d and Supplementary Fig. 6j), replicating the MCF7AC1 response. We then went on to compare ENDX (5 µM) with clinically attainable concentrations of TAM and ICI (0.1 µM) in terms of their effects on apoptosis. Interestingly, unlike ENDX, both TAM and ICI failed to induce apoptosis (Supplementary Fig. 7).

### Expression of constitutively active AKT attenuates ENDX-induced apoptosis in ERα+ breast cancer cells

**Fig. 9:**
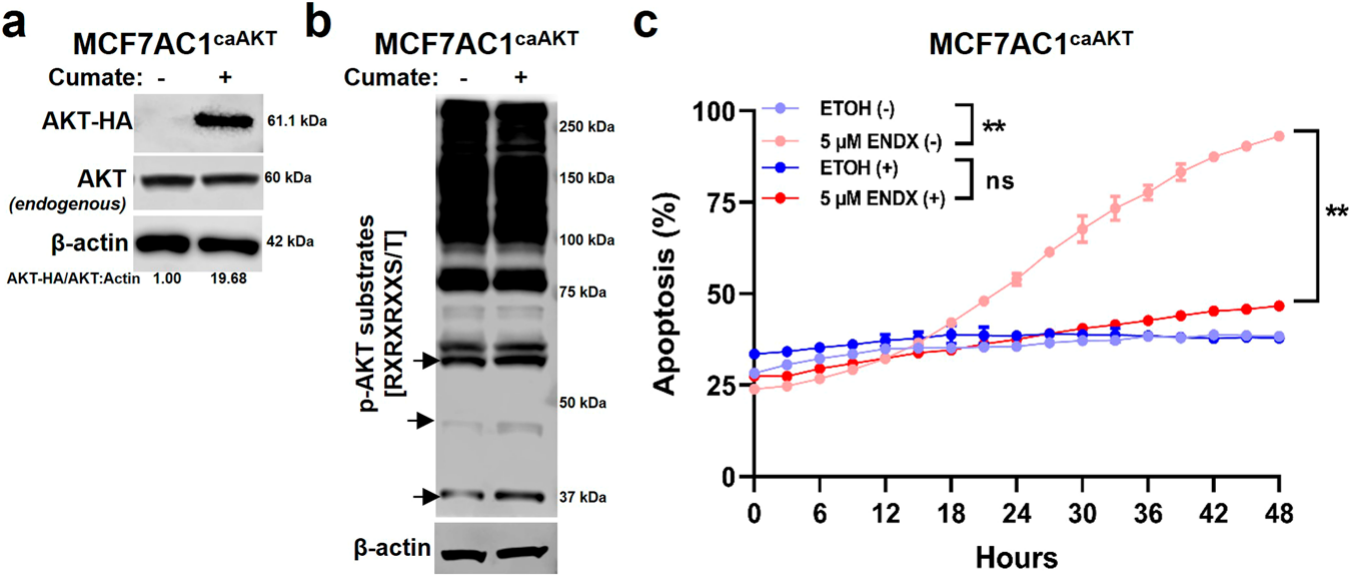
Expression of catalytically active AKT diminishes ENDX ability to induce apoptosis. **a** MCF7AC1^caAKT^ cells were grown in FBS medium in the absence (-) or presence (+) of cumate for 48 h. IB assay of C-terminally hemagglutinin tagged AKT (AKT-HA), endogenous AKT and β-actin. **b** MCF7AC1^caAKT^ cells grown in FBS medium in the (-) or (+) of cumate for 48 h. IB assay of pAKT-substrates and β-actin. **c** MCF7AC1^caAKT^ cells grown in CSS medium were treated for 48 h in the (-) or (+) of cumate, following which cells were co-treated with vehicle control or 5 µM ENDX and IncuCyte Annexin V green and NucLight red reagents for 48 h. The percentage (%) of cells undergoing apoptosis was calculated as indicated in Fig. 1b. Data represents the mean of six wells per treatment performed as biological duplicates ± s.d. ns: nonsignificant. **p < 0.01 by one-way ANOVA.

To confirm the role of AKT inhibition in ENDX-induced apoptosis, we utilized a cumate inducible expression system to overexpress a C-terminally HA-tagged constitutively active AKT in MCF7AC1 cells (MCF7AC1^caAKT^ cells). Immunoblot assays with an anti-HA antibody confirmed cumate-induced expression of caAKT (Fig. 9a) that was associated with increased phosphorylation of AKT substrates (Fig. 9b, arrows). While ENDX at 5 µM induced apoptosis in the absence of cumate in this cell model, expression of caAKT significantly diminished the ability of ENDX to induce apoptosis (Fig. 9c). Taken together, these findings establish that ENDX not only inhibits proliferation, but at higher concentrations, induces apoptosis of ERα+ breast cancer cells, and this may occur in part through inhibition of PKCβ1 and the resulting decrease in AKT kinase activity.

Evaluation of ENDX dose response effects (0–10 µM) on apoptosis in MDAMB231 cells cultured in CSS medium showed that ENDX did not induce apoptosis in these cells until it reached the highest concentration of 10 µM (Supplementary Fig. 8a). Concurrent studies performed to evaluate ENDX effects on growth of MDAMB231 cells at the above-mentioned concentrations, revealed that ENDX did not inhibit growth until it reached concentrations of ≥ 7.5 µM (Supplementary Fig. 8b). Given the lack of MDAMB231 response to ENDX treatment, we extended our investigation and evaluated ENDX response in the ER- BT549 and MDAMB436 breast cancer cells. Similar to MDAMB231 cells, ENDX did not inhibit growth of these cells until concentrations > 7.5 µM (Supplementary Fig. 8b). Immunoblot confirmed basal expression of PKCβ1 in all three cell lines (Supplementary Fig. 8c). Collectively, the lack of an effect of ENDX on apoptosis in ER- cells are consistent with the null findings regarding ENDX’s effects on AKT signaling in ER- cells, and suggest that unlike ERα+ cells, ENDX is unable to elicit pharmacodynamic effects on apoptosis through PKCβ1 targeting.

## Discussion

Clinical trials with ENDX at doses ranging from 20 mg/day to 360 mg/day have demonstrated plasma concentrations ranging from 150 nM to 5 µM, with antitumor activity observed in patients with endocrine resistant breast cancer (including patients with prior progression on AIs, TAM, and fulvestrant) as well as in other tumors that do not express ERα, including TAM resistant desmoid and ovarian cancer^12–14^. These clinical studies have suggested the possibility that the antitumor activity of ENDX may extend beyond its ability to potently block ERα. In order to identify additional protein signaling events targeted by ENDX, we performed an unbiased mass spectrometry studies comparing low and high ENDX concentrations in the absence of estradiol (CSS media). As expected in this estrogen depleted experiment and at low ENDX concentrations, ENDX treatment did not substantially alter the phosphoproteome of MCFAC1 cells. However, at higher but clinically achievable concentrations, endoxifen decreased PKCβ1 protein level in the setting of PMA and insulin, inhibited PMA induced AKT phosphorylation, and induced apoptosis. While others have reported that ENDX inhibits PKC kinase activity^39^, our findings suggest a unique effect of ENDX to induce PKCβ1 protein degradation that may be critical for its anticancer effects.

Our further studies with PKCβ siRNA indicate that PKCβ1 downregulation suppresses AKT signaling and attenuates tumor growth, two of the prominent effects observed with ENDX. Furthermore, similar to drugs which directly target AKT, ENDX results in the induction of apoptosis in endocrine-sensitive ERα+ breast cancer under estrogen deprived conditions.

**Fig. 10:**
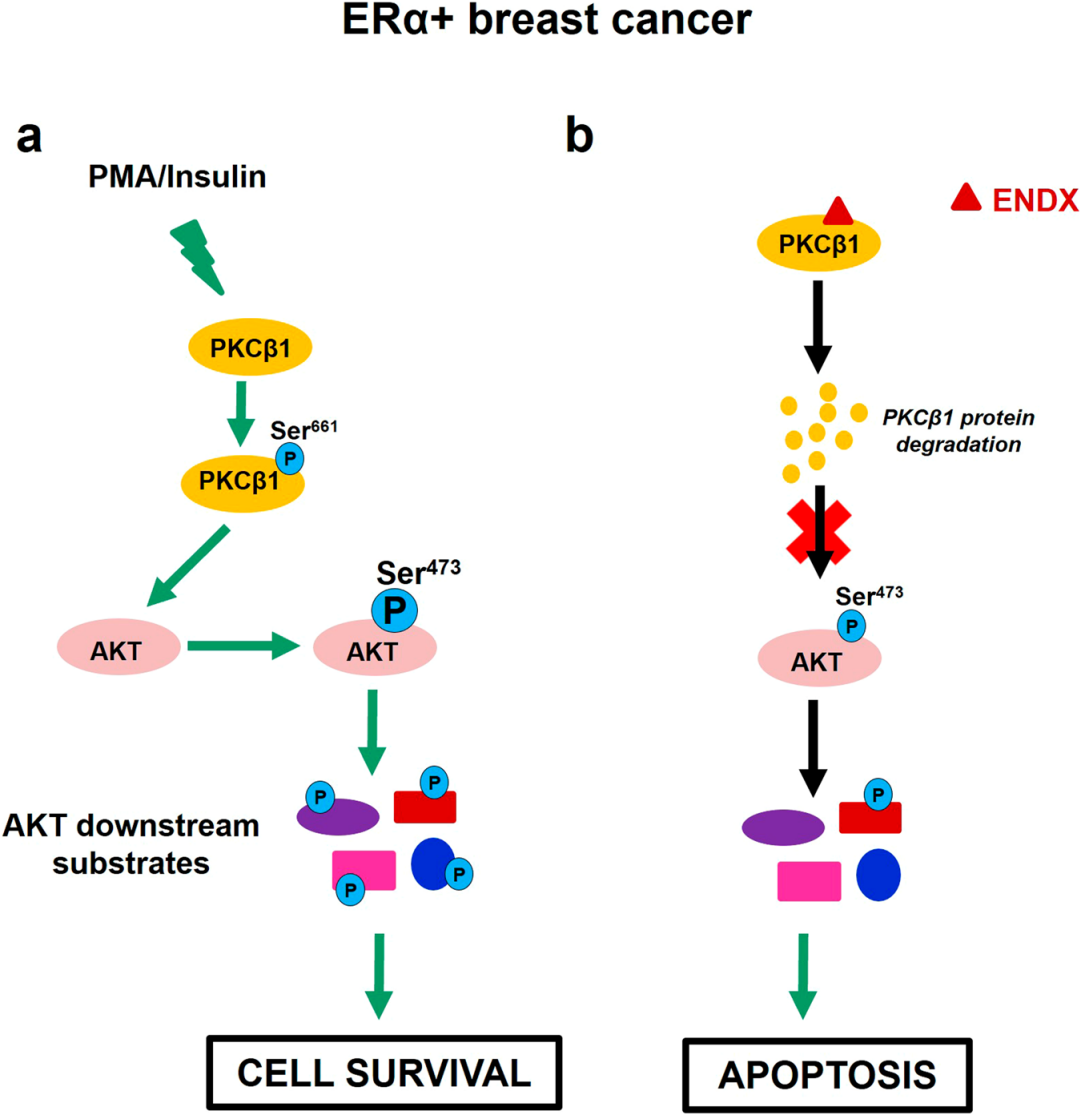
Summary of ENDX anticancer effects in ERα+ breast cancer cells. **a** Activation of PKCβ1^Ser661^ by the PKC agonist PMA and/or insulin phosphorylates AKT^Ser473^ resulting in the activation of p-AKT downstream substrates, which mediates cell survival. **b** ENDX binds to PKCβ1 and facilitates PKCβ1 protein degradation, resulting in the attenuation of phosphorylation of AKT^Ser473^ as well as downstream p-AKT substrates, leading to induction of apoptosis.

Previous studies have demonstrated that TAM and AI do not elicit apoptotic effects in ERα+/HER2- breast cancer patients^40^. In contrast, ENDX began to induce apoptosis at clinically achievable ENDX concentrations of ≥ 2.5 µM (concentrations achieved in the early phase clinical trials), with no effects on PMA induced phosphorylation of AKT nor apoptosis at lower concentrations. Importantly, these effects are distinct from other endocrine therapies, as neither TAM nor ICI (using clinically achievable concentrations) target PKCβ1, inhibit AKT^Ser473^ phosphorylation or induce apoptosis. In total, these observations suggest that ENDX, at higher concentrations, may exhibit a distinct mechanism of action, as shown in Fig. 10, in which ENDX, acting through downregulation of PKCβ1, diminishes AKT-mediated survival signaling, resulting in cell death.

While the apoptotic effects of ENDX were similar to those observed with the AKT inhibitor MK-2206, ENDX induced inhibition of AKT^Ser473^ phosphorylation was less robust compared to MK-2206, suggesting that ENDX induced apoptosis may involve additional effects extending beyond inhibition of AKT signaling.

The parental MCF7 and T47D cell lines harbor E545K and H1047R mutations respectively, in the helical (exon 9) and kinase (exon 20) domain of *PIK3CA* gene^41,42^. Given that ENDX at 5 µM displayed promising antitumor activity in cell line models harboring activating *PIK3CA* mutations, which occur in nearly 40% of patients with advanced ERα+/HER2- breast cancer^43^ and are associated with poor survival outcomes^44^, further studies are ongoing to evaluate the clinical activity of ENDX therapy in breast tumors harboring *PIK3CA* mutations.

In a previous study, Ali et al. utilized racemic endoxifen and demonstrated the drug to be a potent inhibitor of protein kinase C activity compared to TAM^39^. However, that study did not discriminate between the PKC isoforms. Here, we identified that ENDX is a potent inhibitor of PKCβ1 kinase activity and moreover, ENDX may promote PKCβ1 protein degradation, opening up the exciting possibility for the potential development of PKCβ1-targeting ENDX PROTACs for ERα+ breast cancer. Interestingly, ENDX induced degradation of PKCβ1 protein in both PMA and insulin treated cells was associated with the biological effects of apoptosis, suggesting that PKCβ1 degradation may be needed for the biological effect of ENDX to inhibit AKT signaling.

The role of PKCβ in promoting tumorigenesis is well documented^45–47^. However, clinical trials testing ATP competitive PKC kinase inhibitor enzastaurin have demonstrated a lack of antitumor activity in multiple solid tumor clinical trials, including breast cancer^48,49^. The present study demonstrated that while enzastaurin potently inhibits PKCβ1 kinase activity, it displayed no effects on PKCβ1 protein level nor AKT^Ser473^ phosphorylation, which might explain its lack of antitumor effects. These findings also strengthen the notion that the potent anticancer effects of ENDX mediated via PKCβ1 is likely driven by additional mechanisms extending beyond ATP competitive inhibition of PKCβ1 kinase. Further study is needed to better understand the structural basis for the binding of ENDX to PKCβ1 and the molecular basis of ENDX mediated PKCβ1 degradation.

A critical finding from our study was that PMA was unable to activate AKT signaling in ER- cells. Furthermore, in these same cells, ENDX was unable to induce PKCβ1 protein degradation, nor block AKT signaling in ER- cells. These data suggest that the presence of ERα may be critical for PMA induced AKT signaling and that PKCβ1 is unlikely to be a viable drug target for ENDX in ER- cell lines.

ENDX has been reported to elicit additional nontumor effects that extends beyond its anticancer activity. In preclinical studies, treatment with both low (10 mg/kg) and high (50 mg/kg) doses of ENDX elicited beneficial effects on the bone in ovary-intact and ovariectomized animal models by increasing cancellous and cortical bone mass, reducing bone turnover, and protecting against bone loss^50,51^. Also in intact rats, ENDX treatment substantially lowered uterine weight and increased epithelial cell height. In terms of ENDX effects on gene expression, in intact rats ENDX treatment significantly decreased the mRNA expression PGR, SFRP4, and RALDH2 and increased the mRNA expression of IGF1 whereas in ovariectomized rats, ENDX treatment significantly increased the expression of PGR and IGF1 and decreased the expression of proliferating cell nuclear antigen (PCNA) and IGF1R^51^. A separate study evaluating ENDX effects in ovariectomized Sprague-Dawley rats have shown that ENDX is less potent than estradiol in eliciting uterotrophic effects^52^.

In summary, we have demonstrated a dose-dependent effect of ENDX on the phosphoproteome and identified AKT signaling as an important pathway targeted by ENDX. We further implicate PKCβ1 in mediating ENDX effects on AKT. These results suggest that the ability of ENDX to dually target ERα and PKCβ1 may result in greater anticancer actions relative to other endocrine targeting agents in ERα+ breast cancer. These effects are being studied in an ongoing neoadjuvant study evaluating ENDX for the treatment of premenopausal women with ERα+/HER2- breast cancer (EVANGELINE) (NCT05607004).

## Methods

### Cell culture

MCF7 human breast cancer cells stably transfected with the aromatase gene (MCF7AC1)^53^ (a kind gift from Angela H. Brodie, University of Maryland, Baltimore, MD) were cultured in phenol-red free IMEM medium (Gibco #A10488-01) supplemented with 10% fetal bovine serum (FBS) (Gemini #900-108), 600 μg/ml geneticin (G418) (Gibco #10131-027) and 1% Antibiotic-Antimycotic (AA) (Gibco #15240-062). To maintain an estrogen deprived state, MCF7AC1 cells were cultured in IMEM medium containing 10% CSS (Hyclone #SH30068), 600 μg/ml G418, and 1% AA. T47D cells (a kind gift from John R. Hawse, Mayo Clinic, Rochester, MN) were cultured in DMEM/F12 medium (Corning #16-405-V) containing 10% FBS and 1% AA. T47D-long-term estrogen deprived (LTED) cells (a kind gift from John R. Hawse, Mayo Clinic, Rochester, MN) were cultured in DMEM/F12 medium containing 10% CSS and 1% AA. C-terminally hemagglutinin (HA)-tagged, catalytically active AKT expressing MCF7AC1 (MCF7AC1^caAKT^) cells were cultured in IMEM containing 10% FBS, 600 μg/ml G418, 1% AA and 0.5 µg/ml puromycin (Gibco #A11138-03). The ER- MDAMB231, BT549, and MDAMB436 breast cancer cells (a kind gift from John R. Hawse, Mayo Clinic, Rochester, MN) were cultured in DMEM/F12 medium containing 10% FBS and 1% AA. The HEK293F cells (a kind gift from Matthew J. Schellenberg, Mayo Clinic, Rochester, MN) were cultured in DMEM medium (Corning #34722014) containing 10% FBS and 1X penicillin-streptomycin (Sigma #P0781). The Doxycycline-inducible ERα-expressing MDAMB231 cell line was established using the T-REx^TM^ system (InVitrogen) as previously described^54,55^ and were maintained in DMEM/F12 medium containing 10% FBS 1% AA, 5 mg/L Blasticidin S (Sigma #15205) and 500 mg/L Zeocin (InVivoGen #ant-zn-5b).

The ENDX hydrochloride utilized in this study was synthesized in collaboration between Mayo Clinic and National Institute of Health (NIH). Estrogen deprived MCF7AC1 were treated with vehicle control or ENDX (National Cancer Institute) for 24 h. For the phorbol 12-myristate 13-acetate (PMA) (LC Laboratories, #P-1680) experiments, MCF7AC1 cells were maintained in serum-free medium for 24 h prior to pretreatment with vehicle or ENDX for 2 h followed by treatment with 20 or 200 nM PMA for 20 min. For the insulin (Sigma-Aldrich, #I0516) experiments, MCF7AC1 and T47D cells were maintained in serum-free medium for 24 h prior to pretreatment with vehicle control or drugs for 2 h followed by treatment with 100 nM insulin for 1 h.

### Proliferation assay

Cells were plated at a density of 2000 cells per well. Cell viability of (i) vehicle or drug treated MCF7AC1 and T47D-LTED cells in CSS medium, (ii) siNT or siPKCβ-transfected MCF7AC1 cells in CSS medium and (iii) T47D cells in FBS versus CSS medium were analyzed by crystal violet staining assay after 6 days of treatment or siRNA transfection as previously described^11^. Cell viability was calculated as the average absorbance of the drug treated cells divided by the average absorbance of the vehicle treated cells × 100.

### Apoptosis assay

MCF7AC1 and T47D-LTED cells were plated at a density of 2000 cells per well in CSS medium for 24 h. Cells were then co-treated for 48 h with vehicle or drug, IncuCyte Annexin V green reagent (#4642, 1:300), an early-stage apoptosis marker, and IncuCyte NucLight rapid red reagent (#4717, 1:500), a dye that stains all cell nuclei red, in CSS medium. MCF7AC1^caAKT^ cells were plated at a cell density of 2000 cells per well in CSS medium in the absence or presence of cumate for 48 h and then co-treated with vehicle or drug, Annexin V green and NucLight rapid red reagents in the absence or presence of cumate for an additional 48 h in CSS medium. The apoptosis graphs are presented as the green object count (which correspond to cells that are stained with the IncuCyte green fluorescence Annexin V reagent) divided by the red object count (which correspond to the total number of cells in the culture that are stained with the IncuCyte red fluorescence Nuclight Rapid Red Cell Labeling reagent that labels the nucleus of all cells without perturbing cell function or biology) and displayed as percentage (%) using the IncuCyte S3 analysis software.

### Mass spectrometry-based quantitative proteomics analysis

Following treatment of MCF7AC1 cells with vehicle control or 0.01, 0.1 or 5 µM ENDX for 24 h in CSS media, cells were harvested and lysed in 8 M urea buffer (8 M urea, 20 mM HEPES pH 8.0, 1 mM sodium orthovanadate, 2.5 mM sodium pyrophosphate, 1 mM β-glycerophosphate, and 5 mM sodium fluoride), followed by sonication, and centrifugation at 15,000 × *g* at 4 °C for 20 min to clear cell debris. BCA Protein Assay was used to measure the protein concentration. 2 mg of protein lysates from each treatment condition was used for digestion with trypsin. Briefly, the protein lysates were reduced with 5 mM dithiothreitol at 37 °C for 1 h and alkylated with 10 mM iodoacetamide at room temperature in dark for 30 min. The protein lysates were then diluted in 20 mM HEPES pH 8.0 to a final concentration <2 M urea and digested with TPCK-treated trypsin (Worthington Biochemical Corp. Lakewood, NJ) overnight at room temperature. Digested peptides were acidified with 20% trifluoroacetic acid (TFA) to a final concentration of 1% TFA. The tryptic peptides were desalted using SepPak C_18_ cartridge (Waters Corporation, Milford, MA). Eluted peptides were lyophilized and stored at −80 °C prior to Tandem Mass Tag (TMT) labeling.

For the TMT labeling of peptides and basic reversed-phase liquid chromatography (bRPLC) fractionation, the lyophilized tryptic peptides were reconstituted in 150 µl 100 mM triethylammonium bicarbonate (TEABC) and measured with peptide BCA assay (Thermo Scientific). 1 mg peptides from each sample in a final volume of 100 μl 1 mM TEABC were mixed with 1 mg µg TMTpro reagent that was dissolved in 20 µl anhydrous acetonitrile. After 1 h incubation at RT, 10 μL of 5% hydroxylamine was added and incubated for 15 min at RT to quench the labeling reaction. Peptides labeled by different TMT reagents were then mixed and dried with Speed-Vac. The dried TMT-labeled peptides were reconstituted in 20 mM ammonium formate and fractionated by high-pH reversed-phase liquid chromatography on Dionex Ultimate 3000 (Thermo Scientific). Peptides (12 mg) were separated on a 4.6 mm × 50 cm × 3.5 μm Xbridge column (Waters) with a 2-h gradient from 2 to 40% mobile phase B (MPB). Mobile phase A was composed of 20 mM ammonium formate in water, and MPB was composed of 20 mM ammonium formate in 80% acetonitrile. A total of 96 fractions were collected and concatenated into 24 fractions. A 20-μg equivalent of each fraction was set aside for global proteome analysis, and the rest of each sample was concentrated into 12 fractions and dried before phosphopeptide enrichment.

For the phosphopeptide enrichment, each fraction was reconstituted in 1 ml of 80% acetonitrile in 0.1% TFA. Phosphopeptides were enriched using an immobilized metal affinity chromatography (IMAC) approach. In brief, nickel-nitrilotriacetic (Ni-NTA) superflow agarose beads were stripped of nickel with 100 mM EDTA, incubated with 10 mM FeCl_3_ solution and equilibrated in 80% ACN/0.1%TFA. 10 μl IMAC beads were mixed with each fractionated peptide in 80% acetonitrile/0.1% TFA and rotated for 30 min at RT. Subsequently, incubated IMAC beads were washed with 500 μl 80% ACN/0.1%TFA four times and 500 μL 0.1% FA one time. Phosphopeptides were eluted from IMAC beads with 200 μL of 500 mM dibasic sodium phosphate (pH 7.0) for three times. The eluted phosphopeptides were desalted with C18 Stage Tips, and Speed-Vac dried.

For the LC-MS/MS analysis, the peptide fractions were loaded on a 2 cm trap column (Acclaim PepMap 100, C_18_, 5 µm particle size, 100 µm i.d. 100 Å pore size, Thermo Scientific, San Jose, CA) using 0.1% formic acid with a flow rate 20 µl/min for 4 min. The peptides were separated on a 50 cm analytical column (Acclaim PepMap 100, C_18_, 2 µm particle size, 75 µm i.d. 100 Å pore size, Thermo Scientific, San Jose, CA) with a 135 min gradient from 3% to 40% acetonitrile in 0.1% formic acid at a flow rate of 0.3 µl/min. The spray voltage was set to 2.3 kV while capillary temperature was set to 275 °C. The samples were analyzed on an Orbitrap Fusio Lumos mass spectrometer (Thermo Scientific, Bremen, Germany). The MS instrument was operated in data-dependent acquisition mode. A survey full scan MS (from 350–1500 *m/z*) was acquired in the Orbitrap with resolution 120,000 at *m/z* 200 with a maximum AGC target value of 800,000 ions. The data-dependent MS/MS was carried out using Top Speed method with a duty cycle of 2 s. Singly charged precursor ions were excluded while precursor ions with charge states 2–7 were sequentially isolated and fragmented in the higher-energy collisional dissociation cell using 34% normalized collision energy (NCE). The maximum ion injection time for MS and MS/MS were set to 50 ms. Fragment ion spectra were detected in Orbitrap mass analyzer with a resolution 30,000 at *m/z* 200. Dynamic exclusion was enabled one event of fragmentation followed by exclusion of the precursor for next 45 s within 7 ppm of the selected *m/z*. For all measurements with the Orbitrap detector, a lock-mass ion from ambient air (*m/z* 445.120025) was used for internal calibration.

### Mass spectrometry data analysis

Proteome Discoverer software suite (v 2.5; Thermo Fisher Scientific, San Jose, CA) was used for quantitation and database searches. The MS/MS data were searched using the SEQUEST search algorithm against a Human Uniport protein database supplemented with frequently observed contaminants. Search parameters included trypsin as a protease with full specificity and a maximum of two allowed missed cleavages; carbamidomethylation of cysteine and TMTpro tag (+304.207 Da) on lysine residues or peptide N-terminus as a fixed modification; oxidation at methionine and phosphorylation at serine/threonine/tyrosine as variable modifications. The precursor tolerance was set at 10 ppm, while the fragment match tolerance was set to 0.02 Da. The PSMs, peptides and proteins were filtered at 1% false discovery rate cut-off calculated using target-decoy database searches. The probability of an identified phosphorylation of specific Ser/Thr/Tyr residue on each identified phosphopeptide was determined from the PhosphoRS algorithm.

### Phosphoproteome data analysis

The intensities of TMT reporter ions were normalized based on the average total phosphopeptide intensity detected in each TMT labeling channel. Differentially phosphorylated sites were identified with an empirical Bayesian moderated t-statistics test as implemented in the R limma package. Multiple comparison correction was performed with Benjamini-Hochberg procedure. Phosphorylation sites with log2 fold change >1.5 and unadjusted p-value < 0.05 were selected for downstream analysis.

DAVID, an integrated online functional annotation tool^56^, was used to annotate the functions of the differentially modulated phosphoproteins. Kyoto Encyclopedia of Genes and Genomes (KEGG) database^57^ was selected to identify enriched signaling pathways. The ggplot package in R was used to generate the bubble plot depicting the enriched pathways. The Fuzzy C-means clustering showing the dynamic regulation patterns of phosphosites were generated using ggplot and mfuzz packages in R. Kinase substrate enrichment analysis (KSEA)^58^, PhosphoSitePlus^59^, NetworKIN^21^ and RoKAI^22^ datasets were used to predict upstream kinases of regulated phosphosites, as described below. To identify the motifs enriched in the ENDX-regulated phosphosites, MoMo program^60^ with motif-x^61^ algorithm were used.

### Upstream kinase prediction analysis

The 325 phosphosites in Cluster 1 was provided as an input in the NetworKIN and RoKAI kinase prediction tools. While NetworKIN predicted upstream kinases for 32 (10%) of the 325 phosphosites, RoKAI predicted upstream kinases for 14 (4%) of the 325 phosphosites. For the remaining 279 phosphosites no upstream kinase predictions were provided by these prediction tools. Taken together, both NetworKIN and RoKAI predicted a total of 46 upstream kinases for only 14% of the phosphosites in Cluster 1 and these kinases were graphed in the X-axis of Fig. 3a. The number of counts in the Y-axis refers to the total number of phosphosites substrates for which the given kinase is predicted as a potential upstream kinase. For example, PKCB is predicted as the upstream kinase for 5 phosphosites, CDK1 is predicted as the upstream kinase for 4 phosphosites, and AKT1 is predicted as the upstream kinase for 3 phosphosites, respectively in Cluster 1.

### Immunoblot (IB) analysis

Protein lysates were prepared using the RIPA lysis buffer system (ChemCruz #sc-24948) and quantified using the DC^TM^ Protein Assay reagents (Bio-Rad #5000112). Equal amounts of protein lysates were separated on 10% Criterion gels (Bio-Rad #3450112), transferred to PVDF membranes (Bio-Rad #1620177), blocked in TBST-5% milk and probed with primary antibodies listed in Supplementary Table 5, at the indicated dilutions. Membranes were incubated with HRP-conjugated anti-rabbit (CST #7074) or anti-mouse (CST #7076) secondary antibodies and visualized using chemiluminescent West Pico (Thermo Scientific #34580) or West Femto (Thermo Scientific #34096) reagents and a Li-Cor Odyssey® XF imager. Protein lysates from the MCF7AC1 xenograft model were obtained from a previous study^11^. Quantitation of the protein bands signal intensity was performed using the NIH ImageJ image analysis software (https://imagej.nih.gov/ij///index.html). For the quantitation of the phospho protein levels, we first normalized total protein levels to β-actin loading control and used these values to normalize the phospho protein levels and compared change in protein expression levels relative to vehicle control normalized to 1.0. For the quantitation of total protein levels, we normalized the total protein levels to β-actin and compared change in expression levels relative to vehicle control normalized to 1.0. All blots were processed in parallel and derived from the same experiments. Unprocessed immunoblot images are provided in Supplementary Figs. 9–13.

### In vitro kinase assay

For the evaluation of ENDX effects on the kinase activity of the protein kinase C (PKC) family members and TAM effects on the kinase activity of PKC beta 1 (PKCβ1), the drug compounds were tested in a 10-dose IC_50_ mode with three-fold serial dilution starting at 50 μM, in the presence of 10 μM ATP. Staurosporine, a broad-spectrum kinase inhibitor and a positive control, was also tested as described above. The IC_50_ concentrations of these drugs in inhibiting PKC kinase activity are provided in Supplementary Table 4.

### Affinity measurements by Surface Plasma Resonance (SPR)

Binding assays were performed at 25 °C on a Biacore T200 biosensor (GE healthcare). Purified PKCβ1 protein were immobilized on a CM5 S sensor chip using amino coupling and immobilization buffer (10 mM HEPES, 150 mM NaCl, pH 7.4, P20 0.01% (w/w)) and acetate pH 5.0 at a flow rate of 10 µL/min and reaching 10,000-12,000 resonance units (RUs). ENDX at concentrations ranging from 0–8000 nM in phosphate buffer (Gibco) with 2% DMSO (v/v) and 0.01% (w/w) P20 were run over the chips at a flow rate of 50 µl/min for 30 s. Binding kinetics were derived from sensograms using Biacore BIA evaluation software (GE). Sensograms were subtracted for background contributions, and affinity constants were derived using a steady-state affinity fitting of a 1:1 interaction model.

### siRNA transfection

MCF7AC1 cells were maintained in CSS medium and transfected with non-targeting siRNA (siNT) (SI03650325) or a pool of two different siRNA’s targeting total PKCβ (Hs_PRKCβ1_6 SI00605948 and Hs_PRKCβ1_4 SI00042273) (siPKCβ) (Qiagen) at 5 nM concentration in the presence of lipofectamine RNAiMAX transfection reagent (13778-075, Invitrogen) for up to 48 h for IB analysis and for up to 6 days for proliferation assays in biological triplicates.

The custom made siPKCβ1 siRNA (sense: 5′-AAGCCAAAAGCUAGAGACAUU-3′; antisense: 5′-UGUCUCUAGCUUUUGGCUUUU-3′) or the siGENOME non-Targeting siRNA Pool #1 (#D-001206-13-20) from Dharmacon were transfected into MCF7AC1 cells maintained in CSS medium at 40 nM concentration in the presence of DharmaFECT 1 (Dharmacon #T-2001-03) for up to 72 h for IB assay.

A SMART vector inducible human PRKCB mCMV-TurboGFP lentiviral shRNA (Horizon Discovery #V3SH7675-01EG5579) was used for the generation of doxycycline (dox)-inducible PRKCB gene silencing. MCF7AC1 cells grown in 6-well plate in IMEM medium containing 10% FBS, 600 μg/ml G418 and 1% amino acids and at a confluency of 50% were infected with the lentivirus at a multiplicity of infection of 5.0 in the presence of 1 µg/ml polybrene. After 48 h, 10 mg/ml puromycin (Gibco #A11138-03) was added to cells at a dilution of 1 µl per 10 ml to allow for the selection of lentivirus-transfected cells. The expression of the shRNA sequence was induced in the presence of 1 µg/ml of doxycycline (Sigma-Aldrich #D3072) for 72 h for IB assay. The target PRKCB gene sequence used is CAGTGTTGATGGCTGGTTT (Horizon Discovery #V3IHSMCG_9696026).

### Molecular cloning

Molecular cloning was performed for the generation of catalytically active AKT-expressing MCF7AC1 cells. For this purpose, vector pCDNA3.1+ containing an N-terminal SRCMyr signal AKT ORF and C-terminal hemagglutinin (HA)-tag was provided as a kind gift from Dr. Haojie Huang, Ph.D, Mayo Clinic, Rochester, MN. The constitutively active AKT (caAKT)-HA insert was excised using NheI/EcoRV restriction digest, gel purified and cloned into the NheI//PmeI site of an SBI (System biosciences, Palo Alto, CA) vector with modified restriction sites. Sanger sequencing was performed by Azenta/Genewiz, (South Plainfield, NJ) to confirm in frame sequence. After viral transduction of the caAKT-HA construct into MCF7AC1 cells, cells were selected beginning 48 h later for a mixed population (MCF7AC1^caAKT^ cells) using puromycin (Invitrogen) 0.5 µg/ml for several weeks. Expression of caAKT-HA was induced by adding 60 µg/ml cumate (Sigma-Aldrich 268402, 4-Isopropylbenzoic acid) for 48 h prior to drug treatment.

### Statistical analysis

Differences in cell proliferation in the drug treated MCF7AC1 and T47D-LTED cells compared to vehicle treated cells, the % of apoptosis in the drug treated MCF7AC1 and T47D-LTED cells compared to vehicle treated cells and the % of apoptosis in the vehicle or 5 µM ENDX treated MCF7AC1^caAKT^ cells in the presence and absence of cumate were analyzed by one-way ANOVA. Differences in the % of PKCβ1 and AKT^Ser473^ protein levels remaining upon PKCβ1 knockdown in the siNT versus siPKCβ or siPKCβ1 transfected MCF7AC1 cells and in the doxycycline-induced versus noninduced MCF7AC1 cells as well as differences in cell proliferation in the siNT versus siPKCβ transfected MCF7AC1 cells were analyzed by one sample t-test. Comparison of proliferation rates of T47D cells cultured in FBS medium versus CSS medium were analyzed by unpaired t-test. All statistical analysis was performed in Graphpad Prism imaging software (Version 9). A *p* value of <0.05 was considered statistically significant.

### Reporting summary

Further information on research design is available in the Nature Research Reporting Summary linked to this article.

### Supplementary information

Supplementary information file

Reporting Summary

## Data Availability

All mass spectrometry datasets acquired for this study were deposited to ProteomeXchange (http://proteomecentral.proteomexchange.org) and are available via the accession number PXD035007. The reviewer can access the dataset with the Username: reviewer_pxd035007@ebi.ac.uk and password: 9kkfUJ4E. All other relevant data are included in the manuscript or available from the corresponding author upon request.
