# Supplementary material for: Endoxifen downregulates AKT phosphorylation through protein kinase C beta 1 inhibition in ERα+ breast cancer
Source: NPJ Breast Cancer. 2023 Dec 19;9:101. doi: 10.1038/s41523-023-00606-2 (PMC10730845; doi:10.1038/s41523-023-00606-2)
Supplement: Supplementary file 1 — Supplementary information file [file 41523_2023_606_MOESM1_ESM.pdf]

SUPPLEMENTARY INFORMATION

Endoxifen Downregulates AKT Phosphorylation Through Protein Kinase C Beta 1 Inhibition in ERα+ Breast Cancer

Jayaraman, S. *et al*.

SUPPLEMENTARY TABLES:

Supplementary Table 1. List of the 25, 34 and 65 total proteins altered in cells treated with 0.01, 0.1 and 5 µM concentrations of ENDX, respectively, relative to vehicle treated cells based on a fold change (FC) of |1.5| and a p value of < 0.05.

**Accession**	**Gene**	**Description**	**Log FC**	**FC**	**Relative fold change**	**P value**
**0.01 µM ENDX:**						
Q9NRJ7	PCDHB16	Protocadherin beta-16	-0.85	0.554785	-1.802500925	0.0116
P52179	MYOM1	Myomesin-1	-0.692	0.618995	-1.615521555	0.0171
P48740	MASP1	Mannan-binding lectin serine protease 1	-0.678	0.625031	-1.599920257	0.000143
Q9Y680	FKBP7	Peptidyl-prolyl cis-trans isomerase FKBP7	-0.653	0.635957	-1.572434584	0.00356
Q96SR6	ZNF382	Zinc finger protein 382	-0.624	0.648869	-1.541142217	0.00071
Q9C0D6	FHDC1	FH2 domain-containing protein 1	-0.601	0.659297	-1.516767545	0.0175
Q9C073	FAM117A	Protein FAM117A	-0.586	0.666187	-1.501079098	0.00879
O43147	SGSM2	Small G protein signaling modulator 2	-0.529	0.693035	-1.442928687	0.0047
A4GXA9	EME2	Probable crossover junction endonuclease EME2	-0.445	0.734584	-1.361314116	0.00737
Q5HY98	ZNF766	Zinc finger protein 766	-0.323	0.799406	-1.25092908	0.0133
Q00987	MDM2	E3 ubiquitin-protein ligase Mdm2 =1	-0.314	0.804408	-1.243149669	0.0161
Q53HI1	UNC50	Protein unc-50 homolog	0.145	1.105731	1.105730653	0.0406
Q8TBK2	SETD6	N-lysine methyltransferase SETD6	0.245	1.185093	1.185092771	0.0373
Q9NPI6	DCP1A	mRNA-decapping enzyme 1A	0.286	1.219255	1.219255094	0.000989

Q96GV9	C5orf30	UNC119-binding protein C5orf30	0.447	1.363203	1.363202607	0.00943
Q9NZ53	PODXL2	Podocalyxin-like protein 2	0.517	1.430977	1.43097652	0.0308
Q5SR56	MFSD14B	Hippocampus abundant transcript-like protein 1	0.624	1.541142	1.541142217	0.000804
Q9Y2C4	EXOG	Nuclease EXOG, mitochondrial	0.636	1.554015	1.554014538	0.00332
Q7Z353	HDX	Highly divergent homeobox	0.652	1.571345	1.571345033	0.0228
Q9Y3C7	MED31	Mediator of RNA polymerase II transcription subunit 31	0.76	1.693491	1.693490625	0.0139
P29317	EPHA2	Ephrin type-A receptor 2	0.786	1.724287	1.72428709	1.54E-05
Q14914	PTGR1	Prostaglandin reductase 1	0.881	1.841651	1.841651394	0.00193
Q9UKU6	TRHDE	Thyrotropin-releasing hormone-degrading ectoenzyme	1.18	2.265768	2.265767771	0.0367
Q9BX10	GTPBP2	GTP-binding protein 2	1.41	2.657372	2.657371628	0.00124
P20062	TCN2	Transcobalamin-2	1.71	3.271608	3.271608234	0.00704
**0.1 µM ENDX:**						
Q53HI1	UNC50	Protein unc-50 homolog	-1.3	0.406126	-2.462288827	1.31E-05
Q9C0D6	FHDC1	FH2 domain-containing protein 1	-1.1	0.466516	-2.143546925	0.00428
Q8NE31	FAM13C	Protein FAM13C	-1.04	0.486327	-2.056227653	0.0349
Q8TBK2	SETD6	N-lysine methyltransferase SETD6	-0.951	0.517274	-1.933212194	0.000577
P78310	CXADR	Coxsackievirus and adenovirus receptor	-0.9	0.535887	-1.866065983	0.00703
O14526	FCHO1	F-BAR domain only protein 1	-0.89	0.539614	-1.853176124	0.0233
Q96IP4	TENT5A	Terminal nucleotidyltransferase 5A	-0.853	0.553632	-1.80625302	0.0171
Q52M93	ZNF585B	Zinc finger protein 585B	-0.807	0.571569	-1.74956953	0.0439
O43147	SGSM2	Small G protein signaling modulator 2	-0.781	0.581963	-1.71832151	0.0011
Q9Y680	FKBP7	Peptidyl-prolyl cis-trans isomerase FKBP7	-0.756	0.592136	-1.688801775	0.00304
Q08397	LOXL1	Lysyl oxidase homolog 1	-0.75	0.594604	-1.681792831	0.014
P48740	MASP1	Mannan-binding lectin serine protease 1	-0.67	0.628507	-1.591072968	0.0344
Q5HYA8	TMEM67	Meckelin	-0.658	0.633756	-1.577893682	0.00998
Q14DG7	TMEM132B	Transmembrane protein 132B	-0.656	0.634635	-1.575707772	0.00546
Q9BVX2	TMEM106C	Transmembrane protein 106C	-0.637	0.643049	-1.555092072	0.0345
Q00987	MDM2	E3 ubiquitin-protein ligase Mdm2	-0.618	0.651574	-1.534746096	0.000522

A4GXA9	EME2	Probable crossover junction endonuclease EME2	-0.586	0.666187	-1.501079098	0.00948
Q3ZCT1	ZNF260	Zinc finger protein 260	-0.577	0.670356	-1.491744027	0.0197
O14543	SOCS3	Suppressor of cytokine signaling 3	-0.575	0.671286	-1.489677463	0.00015
Q9BX10	GTPBP2	GTP-binding protein 2	-0.548	0.683968	-1.462057448	0.0131
Q9NRJ7	PCDHB16	Protocadherin beta-16	-0.475	0.719467	-1.38991822	0.042
A4QPH2	PI4KAP2	Putative phosphatidylinositol 4-kinase alpha-like protein P2	-0.423	0.745872	-1.340712592	0.0154
Q96QD8	SLC38A2	Sodium-coupled neutral amino acid transporter 2	0.352	1.276329	1.276328769	0.00252
P27449	ATP6V0C	V-type proton ATPase 16 kDa proteolipid subunit	0.449	1.365094	1.365093718	0.02
Q969W9	PMEPA1	Protein TMEPAI	0.623	1.540074	1.540074348	0.0334
Q9Y3C7	MED31	Mediator of RNA polymerase II transcription subunit 31	0.683	1.605475	1.605474777	0.00321
Q2M1P5	KIF7	Kinesin-like protein KIF7	0.73	1.658639	1.658639092	0.0416
Q9UPV0	CEP164	Centrosomal protein of 164 kDa	0.777	1.713564	1.71356391	0.0194
Q6PJG9	LRFN4	Leucine-rich repeat and fibronectin type-III domain-containing protein 4	0.795	1.735077	1.735077374	2.76E-05
Q96J66	ABCC11	ATP-binding cassette sub-family C member 11	0.847	1.798757	1.798756624	0.0215
Q96PQ0	SORCS2	VPS10 domain-containing receptor SorCS2	1.52	2.86791	2.867910496	0.0123
Q7Z353	HDX	Highly divergent homeobox	2.44	5.426417	5.42641731	0.000359
Q9UKU6	TRHDE	Thyrotropin-releasing hormone-degrading ectoenzyme	3.46	11.00433	11.00433455	0.000822
P20062	TCN2	Transcobalamin-2	4.19	18.25222	18.25221945	7.10E-07
**5 µM ENDX:**						
A6NKB5	PCNX2	Pecanex-like protein 2	-1.66	0.316439	-3.160165247	0.00184
Q99456	KRT12	Keratin, type I cytoskeletal 12	-1.65	0.31864	-3.138336392	3.05E-05
Q9H1X1	RSPH9	Radial spoke head protein 9 homolog	-1.52	0.348686	-2.867910496	0.000772
Q8NE31	FAM13C	Protein FAM13C	-1.51	0.351111	-2.848100391	0.000811

Q8N118	CYP4X1	Cytochrome P450 4X1	-1.47	0.360982	-2.770218936	0.00097
Q4G0N8	SLC9C1	Sodium/hydrogen exchanger 10	-1.41	0.376312	-2.657371628	0.00197
Q53HI1	UNC50	Protein unc-50 homolog	-1.34	0.395021	-2.531513188	0.000179
Q96BF6	NACC2	Nucleus accumbens-associated protein 2	-1.25	0.420448	-2.37841423	0.00278
Q8TBK2	SETD6	N-lysine methyltransferase SETD6	-1.21	0.432269	-2.313376368	1.50E-05
Q52M93	ZNF585B	Zinc finger protein 585B	-1.07	0.476319	-2.099433367	0.00124
Q9HBK9	AS3MT	Arsenite methyltransferase	-1.03	0.48971	-2.042024251	0.0086
P78310	CXADR	Coxsackievirus and adenovirus receptor	-1.02	0.493116	-2.02791896	0.00193
Q9UH03	SEPTIN3	Neuronal-specific septin-3	-0.969	0.51086	-1.957483301	0.000232
Q08397	LOXL1	Lysyl oxidase homolog 1	-0.96	0.514057	-1.945309895	0.00479
Q8N126	CADM3	Cell adhesion molecule 3	-0.939	0.521594	-1.917198877	0.017
P48740	MASP1	Mannan-binding lectin serine protease 1	-0.874	0.545632	-1.832737289	0.00159
Q9C0D6	FHDC1	FH2 domain-containing protein 1	-0.865	0.549046	-1.821339667	0.000301
Q8NAP3	ZBTB38	Zinc finger and BTB domain-containing protein 38	-0.856	0.552482	-1.810012926	0.0117
Q5TG30	ARHGAP40	Rho GTPase-activating protein 40	-0.846	0.556325	-1.797510253	0.0285
Q14DG7	TMEM132B	Transmembrane protein 132B	-0.834	0.560972	-1.782620992	0.00483
O43147	SGSM2	Small G protein signaling modulator 2	-0.822	0.565657	-1.767855062	0.00083
P19235	EPOR	Erythropoietin receptor	-0.82	0.566442	-1.765405993	0.0182
A4QPH2	PI4KAP2	Putative phosphatidylinositol 4-kinase alpha-like protein P2	-0.76	0.590496	-1.693490625	0.00732
Q5SZD1	C6orf141	Uncharacterized protein C6orf141	-0.758	0.591316	-1.691144575	0.00801
P01042	KNG1	Kininogen-1	-0.753	0.593368	-1.685293659	0.0429
Q3ZCT1	ZNF260	Zinc finger protein 260	-0.75	0.594604	-1.681792831	0.00962
Q5T1B0	AXDND1	Axonemal dynein light chain domain-containing protein 1	-0.744	0.597082	-1.674812975	0.0197
Q00987	MDM2	E3 ubiquitin-protein ligase Mdm2	-0.728	0.60374	-1.656341323	0.011
Q8TBP5	FAM174A	Membrane protein FAM174A	-0.716	0.608783	-1.642621402	0.00979
Q9Y680	FKBP7	Peptidyl-prolyl cis-trans isomerase FKBP7	-0.699	0.615999	-1.623379162	0.0015
Q5HY98	ZNF766	Zinc finger protein 766	-0.677	0.625465	-1.598811661	0.00103
O14771	ZNF213	Zinc finger protein 213	-0.667	0.629815	-1.587767862	0.0451

Q68CR1	SEL1L3	Protein sel-1 homolog 3	-0.664	0.631126	-1.584469622	0.00562
O95678	KRT75	Keratin, type II cytoskeletal 75	-0.66	0.632878	-1.580082624	0.00392
Q9H3M7	TXNIP	Thioredoxin-interacting protein	-0.633	0.644834	-1.550786413	0.0018
P10745	RBP3	Retinol-binding protein 3	-0.627	0.647521	-1.544350266	0.000271
Q9HBH9	MKNK2	MAP kinase-interacting serine/threonine-protein kinase 2	-0.621	0.65022	-1.537940831	0.00347
Q9BY08	EBPL	Emopamil-binding protein-like	-0.614	0.653383	-1.53049677	0.0115
Q2M1Z3	ARHGAP31	Rho GTPase-activating protein 31	-0.606	0.657016	-1.522033381	0.0479
P53634	CTSC	Dipeptidyl peptidase 1	-0.602	0.65884	-1.517819253	0.0129
Q8NBQ5	HSD17B11	Estradiol 17-beta-dehydrogenase 11	-0.598	0.660669	-1.513616793	0.0356
O14543	SOCS3	Suppressor of cytokine signaling 3	-0.597	0.661127	-1.512567997	4.26E-05
A4GXA9	EME2	Probable crossover junction endonuclease EME2	-0.536	0.68968	-1.449946833	0.00239
Q96SR6	ZNF382	Zinc finger protein 382	-0.532	0.691595	-1.445932295	0.0429
Q14914	PTGR1	Prostaglandin reductase 1	0.164	1.120389	1.120389214	0.027
Q5SR56	MFSD14B	Hippocampus abundant transcript-like protein 1	0.348	1.272795	1.272794935	0.0449
Q96GV9	C5orf30	UNC119-binding protein C5orf30	0.587	1.50212	1.502119927	0.019
Q86YB7	ECHDC2	Enoyl-CoA hydratase domain-containing protein 2, mitochondrial =2	0.602	1.517819	1.517819253	0.0146
Q96QD8	SLC38A2	Sodium-coupled neutral amino acid transporter 2	0.607	1.523089	1.52308874	5.70E-05
Q9NZ53	PODXL2	Podocalyxin-like protein 2	0.616	1.53262	1.53261996	0.00501
P27449	ATP6V0C	V-type proton ATPase 16 kDa proteolipid subunit	0.618	1.534746	1.534746096	0.00457
P29317	EPHA2	Ephrin type-A receptor 2	0.644	1.562656	1.56265576	0.0256
P36941	LTBR	Tumor necrosis factor receptor superfamily member 3	0.677	1.598812	1.598811661	0.00554
Q96CS7	PLEKHB2	Pleckstrin homology domain-containing family B member 2	0.702	1.626758	1.626758396	0.00361
Q15582	TGFBI	Transforming growth factor-beta-induced protein ig-h3	0.71	1.635804	1.635804117	0.0184

Q9BT67	NDFIP1	NEDD4 family-interacting protein 1	0.721	1.648324	1.64832417	3.21E-06
O95807	TMEM50A	Transmembrane protein 50A	0.734	1.663244	1.663244197	0.0326
Q15858	SCN9A	Sodium channel protein type 9 subunit alpha	0.752	1.684126	1.684125907	0.0178
Q96J66	ABCC11	ATP-binding cassette sub-family C member 11	0.818	1.76296	1.762960316	0.016
Q96DR4	STARD4	StAR-related lipid transfer protein 4	0.89	1.853176	1.853176124	0.000146
Q9NPI6	DCP1A	mRNA-decapping enzyme 1A	0.964	1.950711	1.950710923	3.08E-05
Q8TAP9	MPLKIP	M-phase-specific PLK1-interacting protein	1.01	2.013911	2.0139111	0.012
P20062	TCN2	Transcobalamin-2	2.08	4.228072	4.228072162	0.00199
Q7Z353	HDX	Highly divergent homeobox	2.54	5.81589	5.815890069	3.46E-05
Q9UKU6	TRHDE	Thyrotropin-releasing hormone-degrading ectoenzyme	3.91	15.03236	15.03236399	0.000102

Supplementary Table 2. List of the phosphosites in Cluster 1, 2 and 3 altered by ENDX based on Fuzzy c-mean clustering.

prt_site_ind	Ph_Site_Window	Gene symbol	Accession #	ETOH (1)	ETOH (2)	ETOH (3)	0.01 µM ENDX (1)	0.01 µM ENDX (2)	0.01 µM ENDX (3)	0.1 µM ENDX (1)	0.1 µM ENDX (2)	0.1 µM ENDX (3)	5 µM ENDX (1)	5 µM ENDX (2)	5 µM ENDX (3)
**0.01 µM ENDX:**															
S54	VGMGQKDsYVGDEAQ	ACTA1	P68133	33.21	22.69	24.60	21.31	23.00	20.93	13.47	26.07	16.49	15.49	21.34	12.35
S54	VGMGQKDsYVGDEAQ	ACTA2	P62736	33.21	22.69	24.60	21.31	23.00	20.93	13.47	26.07	16.49	15.49	21.34	12.35
S199	ILTERGYsFTTTAER	ACTB	P60709	43.12	32.29	37.63	19.77	35.52	34.10	14.97	45.56	20.91	14.46	24.55	13.97
T202	ERGYSFTtTAEREIV	ACTB	P60709	52.64	37.71	41.39	31.97	47.84	43.51	23.39	54.00	32.33	27.27	30.23	21.12
S52	VGMGQKDsYVGDEAQ	ACTB	P60709	33.21	22.69	24.60	21.31	23.00	20.93	13.47	26.07	16.49	15.49	21.34	12.35
S54	VGMGQKDsYVGDEAQ	ACTC1	P68032	33.21	22.69	24.60	21.31	23.00	20.93	13.47	26.07	16.49	15.49	21.34	12.35
S199	ILTERGYsFTTTAER	ACTG1	P63261	43.12	32.29	37.63	19.77	35.52	34.10	14.97	45.56	20.91	14.46	24.55	13.97
T202	ERGYSFTtTAEREIV	ACTG1	P63261	52.64	37.71	41.39	31.97	47.84	43.51	23.39	54.00	32.33	27.27	30.23	21.12
S52	VGMGQKDsYVGDEAQ	ACTG1	P63261	33.21	22.69	24.60	21.31	23.00	20.93	13.47	26.07	16.49	15.49	21.34	12.35
S53	VGMGQKDsYVGDEAQ	ACTG2	P63267	33.21	22.69	24.60	21.31	23.00	20.93	13.47	26.07	16.49	15.49	21.34	12.35
S329	IGIHHKNsPPKVTVP	AGGF1	Q8N302	14.03	11.75	13.89	11.78	9.75	12.16	9.26	12.40	9.76	8.47	8.68	9.10
S1549	LLSDLTLsPVPRDSL	AHDC1	Q5TGY3	1.48	4.70	2.22	2.36	3.39	3.20	2.81	2.87	2.58	1.19	1.23	1.25
S134	VVYAKCDsSPDSAED	AHSG	P02765	25.23	17.07	39.56	18.85	40.34	47.26	15.34	39.32	16.40	12.39	18.87	10.62
T260	AEVSKLVtDLTKVHT	ALB	P02768	18.79	18.09	25.57	15.47	26.38	27.61	10.20	30.71	11.88	10.95	14.80	9.21
S443	TKKVPQVsTPTLVEV	ALB	P02768	44.53	47.52	92.04	37.60	81.61	103.03	20.02	84.12	31.96	17.04	41.81	10.40
T446	VPQVSTPtLVEVSRN	ALB	P02768	22.52	21.66	43.70	21.62	36.44	50.19	11.04	43.37	16.12	13.53	18.65	4.66
S451	TPTLVEVsRNLGKVG	ALB	P02768	34.37	40.88	64.06	33.71	68.57	75.79	23.30	65.55	23.86	18.38	35.27	10.07
S132	TQGLDGLsERCAQYK	ALDOA	P04075	8.37	6.85	6.85	6.97	6.88	6.67	4.77	7.42	5.25	2.89	6.33	4.12

S309	YGRALQAsALKAWGG	ALDOA	P04075	10.43	3.68	9.07	3.38	6.57	8.41	1.50	10.71	4.42	2.38	4.50	2.06
S322	VNSTSEEsHDEDEIR	ATAD1	Q8NBU5	13.64	10.42	13.60	10.66	11.70	7.59	9.73	9.36	9.40	7.95	6.43	9.64
S939	VLPLALPsPPRQLSE	ATAD2B	Q9ULI0	3.35	2.35	2.12	2.97	1.13	2.29	2.43	0.93	2.21	1.14	1.18	2.38
S419	LSVSRVGsAAQTRAM	ATP5F1A	P25705	16.22	10.63	12.64	7.17	9.96	13.26	6.17	14.43	8.66	6.61	9.54	5.85
S76	EETGRVLsIGDGIAR	ATP5F1A	P25705	18.79	14.51	18.14	10.86	14.37	15.08	11.60	17.30	13.17	7.23	11.90	7.69
T330	AAPAHRGtPDTDLEV	ATXN1L	P0C7T5	36.17	51.40	33.09	33.91	40.55	35.20	29.28	30.88	27.63	20.76	28.52	25.57
T123	NRHFRHDtPDSSPRR	BUD13	Q9BRD0	11.71	16.96	20.07	12.81	13.55	14.08	11.13	10.29	12.99	10.84	10.61	8.02
S126	FRHDTPDsSPRRVRH	BUD13	Q9BRD0	8.88	8.89	13.12	9.02	10.27	7.31	8.70	6.33	10.04	5.37	7.83	5.42
S197	PRRARHDsPDPSPPR	BUD13	Q9BRD0	10.04	10.42	8.59	7.79	7.19	5.49	4.68	8.61	4.05	5.16	6.00	6.82
S201	RHDSPDPsPPRRPQH	BUD13	Q9BRD0	10.04	10.42	8.59	7.79	7.19	5.49	4.68	8.61	4.05	5.16	6.00	6.82
S80	RFYALSAsFEPFSNK	CALR	P27797	5.02	3.78	4.63	4.10	3.08	5.94	1.68	5.40	4.70	2.89	3.00	0.97
S585	EAEAGAGsPTSTPAP	CAMSAP3	Q9P1Y5	1.67	3.78	2.80	1.33	3.59	4.02	1.22	3.04	2.39	1.34	1.39	1.41
S769	RVPATRRsPGPGPSQ	CAMSAP3	Q9P1Y5	10.81	10.22	8.20	8.40	6.06	7.13	4.87	8.18	6.45	5.58	7.50	5.85
S699	LVAAAPTsPDHSP-	CBARP	Q8N350	4.76	3.68	3.28	2.46	3.29	2.47	4.12	1.86	2.49	1.14	1.18	1.19
S703	APTSPDHsP-	CBARP	Q8N350	4.76	3.68	3.28	2.46	3.29	2.47	4.12	1.86	2.49	1.14	1.18	1.19
S138	LRSQPDAsKEELRLL	CCDC183	Q5T5S1	19.69	10.73	16.88	12.91	22.38	20.57	4.96	21.43	10.32	5.58	11.26	4.87
S190	SSTGSVGsPDQLPLA	CCDC85B	Q15834	3.22	3.78	3.67	0.92	2.46	1.65	2.34	2.02	3.87	0.93	0.96	0.97
S23	NDMKVRKsSTPEEVK	CFL1	P23528	51.23	28.10	46.31	35.14	42.60	46.17	21.05	57.54	24.13	20.24	33.45	11.81
S203	KKAEAAAsALADADA	CHMP2A	O43633	7.47	5.52	6.95	5.53	4.82	5.58	3.46	4.89	3.68	5.16	2.57	5.74
S86	RDNLAQQsFNMEQAN	CHMP5	Q9NZZ3	5.92	6.13	5.31	6.76	7.19	5.03	4.21	5.91	4.88	4.34	2.68	4.33
S100	EDPCHPDsPPAPRAT	CREB3L4	Q8TEY5	3.35	3.88	4.92	4.41	3.90	4.02	4.68	4.72	2.03	3.20	1.18	2.60
T21	LLGGPAGtPPGGGAL	DBP	Q10586	4.63	6.13	4.05	2.97	1.33	4.48	4.87	4.30	2.39	3.82	1.39	1.41
S315	PTYTIPLsPVLSPTL	DCP1A	Q9NPI6	4.12	4.29	2.41	1.28	5.65	3.20	2.99	1.05	4.24	2.58	1.34	1.35
S754	QGHNSPDsPVTSAAK	DDX42	Q86XP3	4.38	5.82	5.98	2.36	5.44	7.95	5.33	5.48	4.88	2.27	3.97	4.22

T261	YKIGGIGtVPVGRVE	EEF1A1	P68104	19.44	15.43	16.59	13.42	15.71	15.91	9.82	23.29	14.28	9.81	13.08	6.07
T261	YKIGGIGtVPVGRVE	EEF1A1P5	Q5VTE0	19.44	15.43	16.59	13.42	15.71	15.91	9.82	23.29	14.28	9.81	13.08	6.07
T261	YKIGGIGtVPVGRVE	EEF1A2	Q05639	19.44	15.43	16.59	13.42	15.71	15.91	9.82	23.29	14.28	9.81	13.08	6.07
T86	TIVQEEDtQPLTEPI	EFTUD2	Q15029	5.15	8.58	9.36	3.59	7.70	3.84	6.27	6.67	4.33	5.37	2.14	3.47
Y189	MRIGAEVyHNLKNVI	ENO1	P06733	18.28	16.04	15.63	13.52	16.12	12.52	10.39	16.71	12.99	8.99	14.04	8.99
S419	RIEEELGsKAKFAGR	ENO1	P06733	55.22	27.08	46.79	28.48	43.83	51.19	18.15	60.41	30.12	22.10	26.91	17.87
Y774	ENSSDPTyTSSLGGK	EPHB4	P54760	7.34	8.89	8.10	6.56	8.42	5.58	7.86	8.69	7.00	6.82	4.50	4.44
S489	WEKTGSHsEPQARGD	FAM120A	Q9NZB2	1.29	2.04	2.03	1.02	3.59	2.10	0.94	3.29	0.92	1.03	1.07	1.08
T34	APPAPEAtPPPASAA	FAM207A	Q9NSI2	1.35	2.25	3.38	1.08	2.16	0.96	0.98	0.89	2.12	1.08	1.13	1.14
S324	QEPLLIGsTKSNMGH	FASN	P49327	7.08	5.01	6.37	5.33	5.44	4.66	3.37	8.10	3.32	4.03	4.40	3.68
S962	PIPKSPFsVAVSPSL	FLNA	P21333	1.29	2.55	2.80	2.25	1.03	2.56	0.94	1.69	0.92	1.03	1.07	1.08
S488	GRGDRRHsSDINHLV	FNBP1L	Q5T0N5	20.98	13.49	19.49	12.50	14.58	13.90	16.75	12.74	18.98	10.74	10.29	13.00
S148	DNSLKIIsNASCTTN	GAPDH	P04406	24.71	16.45	25.86	13.22	21.97	25.32	8.05	27.67	13.72	9.29	12.33	6.17
S266	KKVVKQAsEGPLKGI	GAPDH	P04406	43.12	16.25	39.46	24.08	31.00	35.01	11.88	41.76	20.54	15.28	19.94	9.53
S746	ESCSGLGsTSDDTDV	GAPVD1	Q14C86	21.62	19.72	23.35	17.62	13.45	17.19	17.59	14.85	15.01	14.05	13.40	15.49
T846	YMAKRLEtHYRILFR	GLDC	P23378	13.00	3.07	10.71	10.66	10.16	7.59	6.36	4.98	3.22	1.55	1.61	1.62
Y848	AKRLETHyRILFRGA	GLDC	P23378	13.00	3.07	10.71	10.66	10.16	7.59	6.36	4.98	3.22	1.55	1.61	1.62
S623	FGGCFGRsESPQPKA	GNAS	Q5JWF2	2.96	3.07	3.09	4.10	2.16	2.10	2.15	0.89	3.68	2.27	1.13	1.14
S187	KGNEAVAsRDLSENN	GOLM1	Q8NBJ4	12.74	21.87	17.37	16.19	18.89	13.90	15.91	16.20	13.82	9.60	9.65	7.91
S908	SRRHSKRsHDSDDSD	GPATCH8	Q9UKJ3	10.17	10.73	8.78	7.79	8.11	6.40	7.95	8.27	7.46	6.40	4.61	7.04
T466	GDSPDSStPKLSRAQ	GTSE1	Q9NYZ3	13.51	12.06	15.53	10.04	13.55	16.73	12.91	13.25	12.53	11.88	6.86	8.99
S113	ELAKHAVsEGTKAVT	H2BFS	P57053	30.89	12.16	27.40	15.68	26.69	17.55	18.52	21.43	20.45	10.95	15.12	7.80
S56	VHPDTGIsSKAMGIM	H2BFS	P57053	20.59	13.90	17.66	11.78	17.25	16.64	10.67	19.91	10.78	11.05	13.62	7.80
T97	ITSREIQtAVRLLLP	H2BFS	P57053	44.66	22.99	36.56	24.08	30.69	27.70	14.03	39.15	16.67	19.11	25.73	13.00

T46	PHRYRPGtVALREIR	H3F3A	P84243	25.36	12.16	13.99	10.04	18.68	13.16	6.83	22.27	10.78	7.33	10.51	4.01
T45	PHRYRPGtVALREIR	H3F3C	Q6NXT2	25.36	12.16	13.99	10.04	18.68	13.16	6.83	22.27	10.78	7.33	10.51	4.01
S105	QTKGTGAsGSFKLNK	HIST1H1A	Q02539	59.34	41.08	47.37	36.06	64.37	45.71	35.46	48.51	37.21	28.81	33.24	28.49
S105	QTKGTGAsGSFKLNK	HIST1H1B	P16401	59.34	41.08	47.37	36.06	64.37	45.71	35.46	48.51	37.21	28.81	33.24	28.49
S116	KLNKKAAsGEAKPKA	HIST1H1B	P16401	57.15	22.38	31.55	24.90	48.56	32.09	20.40	33.75	28.92	17.76	23.16	16.03
S102	QTKGTGAsGSFKLNK	HIST1H1C	P16403	59.34	41.08	47.37	36.06	64.37	45.71	35.46	48.51	37.21	28.81	33.24	28.49
S113	KLNKKAAsGEAKPKV	HIST1H1C	P16403	57.15	22.38	31.55	24.90	48.56	32.09	20.40	33.75	28.92	17.76	23.16	16.03
S41	KASGPPVsELITKAV	HIST1H1C	P16403	44.53	33.21	44.67	37.09	57.38	47.72	29.66	45.31	38.60	27.99	29.38	20.64
S103	QTKGTGAsGSFKLNK	HIST1H1D	P16402	59.34	41.08	47.37	36.06	64.37	45.71	35.46	48.51	37.21	28.81	33.24	28.49
S42	KASGPPVsELITKAV	HIST1H1D	P16402	44.53	33.21	44.67	37.09	57.38	47.72	29.66	45.31	38.60	27.99	29.38	20.64
S102	QTKGTGAsGSFKLNK	HIST1H1E	P10412	59.34	41.08	47.37	36.06	64.37	45.71	35.46	48.51	37.21	28.81	33.24	28.49
S113	KLNKKAAsGEAKPKA	HIST1H1E	P10412	57.15	22.38	31.55	24.90	48.56	32.09	20.40	33.75	28.92	17.76	23.16	16.03
S41	KASGPPVsELITKAV	HIST1H1E	P10412	44.53	33.21	44.67	37.09	57.38	47.72	29.66	45.31	38.60	27.99	29.38	20.64
S106	QTRGTGAsGSFKLSK	HIST1H1T	P22492	59.34	41.08	47.37	36.06	64.37	45.71	35.46	48.51	37.21	28.81	33.24	28.49
S114	ELAKHAVsEGTKAVT	HIST1H2BA	Q96A08	30.89	12.16	27.40	15.68	26.69	17.55	18.52	21.43	20.45	10.95	15.12	7.80
S57	VHPDTGIsSKAMSIM	HIST1H2BA	Q96A08	20.59	13.90	17.66	11.78	17.25	16.64	10.67	19.91	10.78	11.05	13.62	7.80
T98	ISSREIQtAVRLLLP	HIST1H2BA	Q96A08	44.66	22.99	36.56	24.08	30.69	27.70	14.03	39.15	16.67	19.11	25.73	13.00
S113	ELAKHAVsEGTKAVT	HIST1H2BB	P33778	30.89	12.16	27.40	15.68	26.69	17.55	18.52	21.43	20.45	10.95	15.12	7.80
S37	RKRSRKEsYSIYVYK	HIST1H2BB	P33778	10.94	5.93	7.53	4.61	6.98	10.42	2.99	6.75	3.78	1.65	3.65	1.73
S39	RSRKESYsIYVYKVL	HIST1H2BB	P33778	16.35	11.75	13.89	11.07	13.45	12.98	9.64	19.74	8.11	5.06	8.79	8.34
S56	VHPDTGIsSKAMGIM	HIST1H2BB	P33778	20.59	13.90	17.66	11.78	17.25	16.64	10.67	19.91	10.78	11.05	13.62	7.80
T97	ITSREIQtAVRLLLP	HIST1H2BB	P33778	44.66	22.99	36.56	24.08	30.69	27.70	14.03	39.15	16.67	19.11	25.73	13.00
S113	ELAKHAVsEGTKAVT	HIST1H2BC	P62807	30.89	12.16	27.40	15.68	26.69	17.55	18.52	21.43	20.45	10.95	15.12	7.80
S56	VHPDTGIsSKAMGIM	HIST1H2BC	P62807	20.59	13.90	17.66	11.78	17.25	16.64	10.67	19.91	10.78	11.05	13.62	7.80

T97	ITSREIQtAVRLLLP	HIST1H2BC	P62807	44.66	22.99	36.56	24.08	30.69	27.70	14.03	39.15	16.67	19.11	25.73	13.00
S113	ELAKHAVsEGTKAVT	HIST1H2BD	P58876	30.89	12.16	27.40	15.68	26.69	17.55	18.52	21.43	20.45	10.95	15.12	7.80
S56	VHPDTGIsSKAMGIM	HIST1H2BD	P58876	20.59	13.90	17.66	11.78	17.25	16.64	10.67	19.91	10.78	11.05	13.62	7.80
T97	ITSREIQtAVRLLLP	HIST1H2BD	P58876	44.66	22.99	36.56	24.08	30.69	27.70	14.03	39.15	16.67	19.11	25.73	13.00
S113	ELAKHAVsEGTKAVT	HIST1H2BH	Q93079	30.89	12.16	27.40	15.68	26.69	17.55	18.52	21.43	20.45	10.95	15.12	7.80
S56	VHPDTGIsSKAMGIM	HIST1H2BH	Q93079	20.59	13.90	17.66	11.78	17.25	16.64	10.67	19.91	10.78	11.05	13.62	7.80
T97	ITSREIQtAVRLLLP	HIST1H2BH	Q93079	44.66	22.99	36.56	24.08	30.69	27.70	14.03	39.15	16.67	19.11	25.73	13.00
S113	ELAKHAVsEGTKAVT	HIST1H2BJ	P06899	30.89	12.16	27.40	15.68	26.69	17.55	18.52	21.43	20.45	10.95	15.12	7.80
S37	RKRSRKEsYSIYVYK	HIST1H2BJ	P06899	10.94	5.93	7.53	4.61	6.98	10.42	2.99	6.75	3.78	1.65	3.65	1.73
S39	RSRKESYsIYVYKVL	HIST1H2BJ	P06899	16.35	11.75	13.89	11.07	13.45	12.98	9.64	19.74	8.11	5.06	8.79	8.34
S56	VHPDTGIsSKAMGIM	HIST1H2BJ	P06899	20.59	13.90	17.66	11.78	17.25	16.64	10.67	19.91	10.78	11.05	13.62	7.80
T97	ITSREIQtAVRLLLP	HIST1H2BJ	P06899	44.66	22.99	36.56	24.08	30.69	27.70	14.03	39.15	16.67	19.11	25.73	13.00
S113	ELAKHAVsEGTKAVT	HIST1H2BK	O60814	30.89	12.16	27.40	15.68	26.69	17.55	18.52	21.43	20.45	10.95	15.12	7.80
S56	VHPDTGIsSKAMGIM	HIST1H2BK	O60814	20.59	13.90	17.66	11.78	17.25	16.64	10.67	19.91	10.78	11.05	13.62	7.80
T97	ITSREIQtAVRLLLP	HIST1H2BK	O60814	44.66	22.99	36.56	24.08	30.69	27.70	14.03	39.15	16.67	19.11	25.73	13.00
S113	ELAKHAVsEGTKAVT	HIST1H2BL	Q99880	30.89	12.16	27.40	15.68	26.69	17.55	18.52	21.43	20.45	10.95	15.12	7.80
S56	VHPDTGIsSKAMGIM	HIST1H2BL	Q99880	20.59	13.90	17.66	11.78	17.25	16.64	10.67	19.91	10.78	11.05	13.62	7.80
T97	ITSREIQtAVRLLLP	HIST1H2BL	Q99880	44.66	22.99	36.56	24.08	30.69	27.70	14.03	39.15	16.67	19.11	25.73	13.00
S113	ELAKHAVsEGTKAVT	HIST1H2BM	Q99879	30.89	12.16	27.40	15.68	26.69	17.55	18.52	21.43	20.45	10.95	15.12	7.80
S56	VHPDTGIsSKAMGIM	HIST1H2BM	Q99879	20.59	13.90	17.66	11.78	17.25	16.64	10.67	19.91	10.78	11.05	13.62	7.80
T97	ITSREIQtAVRLLLP	HIST1H2BM	Q99879	44.66	22.99	36.56	24.08	30.69	27.70	14.03	39.15	16.67	19.11	25.73	13.00
S113	ELAKHAVsEGTKAVT	HIST1H2BN	Q99877	30.89	12.16	27.40	15.68	26.69	17.55	18.52	21.43	20.45	10.95	15.12	7.80
S56	VHPDTGIsSKAMGIM	HIST1H2BN	Q99877	20.59	13.90	17.66	11.78	17.25	16.64	10.67	19.91	10.78	11.05	13.62	7.80
T97	ITSREIQtAVRLLLP	HIST1H2BN	Q99877	44.66	22.99	36.56	24.08	30.69	27.70	14.03	39.15	16.67	19.11	25.73	13.00

S113	ELAKHAVsEGTKAVT	HIST1H2BO	P23527	30.89	12.16	27.40	15.68	26.69	17.55	18.52	21.43	20.45	10.95	15.12	7.80
S37	RKRSRKEsYSIYVYK	HIST1H2BO	P23527	10.94	5.93	7.53	4.61	6.98	10.42	2.99	6.75	3.78	1.65	3.65	1.73
S39	RSRKESYsIYVYKVL	HIST1H2BO	P23527	16.35	11.75	13.89	11.07	13.45	12.98	9.64	19.74	8.11	5.06	8.79	8.34
S56	VHPDTGIsSKAMGIM	HIST1H2BO	P23527	20.59	13.90	17.66	11.78	17.25	16.64	10.67	19.91	10.78	11.05	13.62	7.80
T97	ITSREIQtAVRLLLP	HIST1H2BO	P23527	44.66	22.99	36.56	24.08	30.69	27.70	14.03	39.15	16.67	19.11	25.73	13.00
T46	PHRYRPGtVALREIR	HIST1H3A	P68431	25.36	12.16	13.99	10.04	18.68	13.16	6.83	22.27	10.78	7.33	10.51	4.01
T31	RDNIQGItKPAIRRL	HIST1H4A	P62805	63.58	28.31	49.78	30.84	49.17	46.62	15.53	61.76	27.73	17.35	32.27	7.47
S48	RGGVKRIsGLIYEET	HIST1H4A	P62805	55.73	44.25	44.96	45.80	43.83	53.84	27.13	55.94	34.08	29.64	36.88	27.08
Y52	KRISGLIyEETRGVL	HIST1H4A	P62805	33.98	17.99	20.36	12.60	22.07	20.39	14.13	24.21	15.20	12.81	15.12	10.51
S37	RKRSRKEsYSIYVYK	HIST2H2BC	Q6DN03	10.94	5.93	7.53	4.61	6.98	10.42	2.99	6.75	3.78	1.65	3.65	1.73
S39	RSRKESYsIYVYKVL	HIST2H2BC	Q6DN03	16.35	11.75	13.89	11.07	13.45	12.98	9.64	19.74	8.11	5.06	8.79	8.34
S37	RKRSRKEsYSIYVYK	HIST2H2BD	Q6DRA6	10.94	5.93	7.53	4.61	6.98	10.42	2.99	6.75	3.78	1.65	3.65	1.73
S39	RSRKESYsIYVYKVL	HIST2H2BD	Q6DRA6	16.35	11.75	13.89	11.07	13.45	12.98	9.64	19.74	8.11	5.06	8.79	8.34
S113	ELAKHAVsEGTKAVT	HIST2H2BE	Q16778	30.89	12.16	27.40	15.68	26.69	17.55	18.52	21.43	20.45	10.95	15.12	7.80
S37	RKRSRKEsYSIYVYK	HIST2H2BE	Q16778	10.94	5.93	7.53	4.61	6.98	10.42	2.99	6.75	3.78	1.65	3.65	1.73
S39	RSRKESYsIYVYKVL	HIST2H2BE	Q16778	16.35	11.75	13.89	11.07	13.45	12.98	9.64	19.74	8.11	5.06	8.79	8.34
S56	VHPDTGIsSKAMGIM	HIST2H2BE	Q16778	20.59	13.90	17.66	11.78	17.25	16.64	10.67	19.91	10.78	11.05	13.62	7.80
T97	ITSREIQtAVRLLLP	HIST2H2BE	Q16778	44.66	22.99	36.56	24.08	30.69	27.70	14.03	39.15	16.67	19.11	25.73	13.00
S113	ELAKHAVsEGTKAVT	HIST2H2BF	Q5QNW6	30.89	12.16	27.40	15.68	26.69	17.55	18.52	21.43	20.45	10.95	15.12	7.80
S56	VHPDTGIsSKAMGIM	HIST2H2BF	Q5QNW6	20.59	13.90	17.66	11.78	17.25	16.64	10.67	19.91	10.78	11.05	13.62	7.80
T97	ITSREIQtAVRLLLP	HIST2H2BF	Q5QNW6	44.66	22.99	36.56	24.08	30.69	27.70	14.03	39.15	16.67	19.11	25.73	13.00
T46	PHRYRPGtVALREIR	HIST2H3A	Q71DI3	25.36	12.16	13.99	10.04	18.68	13.16	6.83	22.27	10.78	7.33	10.51	4.01
S113	ELAKHAVsEGTKAVT	HIST3H2BB	Q8N257	30.89	12.16	27.40	15.68	26.69	17.55	18.52	21.43	20.45	10.95	15.12	7.80
S37	RKRGRKEsYSIYVYK	HIST3H2BB	Q8N257	10.94	5.93	7.53	4.61	6.98	10.42	2.99	6.75	3.78	1.65	3.65	1.73

S39	RGRKESYsIYVYKVL	HIST3H2BB	Q8N257	16.35	11.75	13.89	11.07	13.45	12.98	9.64	19.74	8.11	5.06	8.79	8.34
S56	VHPDTGIsSKAMGIM	HIST3H2BB	Q8N257	20.59	13.90	17.66	11.78	17.25	16.64	10.67	19.91	10.78	11.05	13.62	7.80
T46	PHRYRPGtVALREIR	HIST3H3	Q16695	25.36	12.16	13.99	10.04	18.68	13.16	6.83	22.27	10.78	7.33	10.51	4.01
S185	TPLPSLAsPAVPAPG	HJURP	Q8NCD3	6.56	5.52	5.11	3.69	5.95	5.21	6.08	7.85	3.87	2.38	3.65	3.79
S397	TAVQYIEsSDSEEIE	HLTF	Q14527	6.31	5.72	6.56	7.38	2.77	5.03	5.05	5.91	3.41	3.10	3.22	2.60
S398	AVQYIESsDSEEIET	HLTF	Q14527	6.31	5.72	6.56	7.38	2.77	5.03	5.05	5.91	3.41	3.10	3.22	2.60
S400	QYIESSDsEEIETSE	HLTF	Q14527	6.31	5.72	6.56	7.38	2.77	5.03	5.05	5.91	3.41	3.10	3.22	2.60
S121	KGEHPGLsIGDVAKK	HMGB1	P09429	36.55	15.02	28.27	15.68	25.66	28.07	6.92	32.57	12.07	12.60	15.76	5.20
S121	KGEHPGLsIGDVAKK	HMGB1P1	B2RPK0	36.55	15.02	28.27	15.68	25.66	28.07	6.92	32.57	12.07	12.60	15.76	5.20
S365	GGSSSSSsYGSGRR-	HNRNPA1	P09651	9.65	6.44	7.72	8.91	6.26	5.21	4.58	5.48	4.88	5.89	4.61	4.77
T110	GKPGAHVtVKKLFVG	HNRNPA2B1	P22626	11.20	10.42	15.53	10.55	11.81	14.72	7.77	12.15	6.08	6.30	8.36	3.36
S391	FIRGVVDsEDLPLNI	HSP90AA1	P07900	19.56	15.43	17.17	12.70	18.17	14.44	4.30	20.59	11.97	7.54	11.26	5.74
S68	YESLTDPsKLDSGKE	HSP90AA1	P07900	33.08	17.99	34.06	20.49	23.00	26.97	9.82	36.19	16.95	15.90	21.44	11.05
S68	YESLTDPsKLDSGKE	HSP90AA2P	Q14568	33.08	17.99	34.06	20.49	23.00	26.97	9.82	36.19	16.95	15.90	21.44	11.05
S383	FIRGVVDsEDLPLNI	HSP90AB1	P08238	19.56	15.43	17.17	12.70	18.17	14.44	4.30	20.59	11.97	7.54	11.26	5.74
S434	KKFYEAFsKNLKLGI	HSP90AB1	P08238	6.44	2.55	4.82	0.97	3.08	3.84	2.43	5.48	2.86	1.96	2.57	1.03
S48	RELISNAsDALDKIR	HSP90AB1	P08238	32.56	19.82	31.16	21.31	29.57	24.59	14.78	34.76	16.58	11.98	19.62	7.80
S63	YESLTDPsKLDSGKE	HSP90AB1	P08238	33.08	17.99	34.06	20.49	23.00	26.97	9.82	36.19	16.95	15.90	21.44	11.05
S48	WELISNAsDALDKIR	HSP90AB2P	Q58FF8	32.56	19.82	31.16	21.31	29.57	24.59	14.78	34.76	16.58	11.98	19.62	7.80
S63	YESLTDPsKLDSGKE	HSP90AB2P	Q58FF8	33.08	17.99	34.06	20.49	23.00	26.97	9.82	36.19	16.95	15.90	21.44	11.05
S325	FIHGVVDsEDLPLNI	HSP90AB3P	Q58FF7	19.56	15.43	17.17	12.70	18.17	14.44	4.30	20.59	11.97	7.54	11.26	5.74
S48	QELISNAsDALDKIR	HSP90AB3P	Q58FF7	32.56	19.82	31.16	21.31	29.57	24.59	14.78	34.76	16.58	11.98	19.62	7.80
S63	YESLTDPsKLDSGKE	HSP90AB3P	Q58FF7	33.08	17.99	34.06	20.49	23.00	26.97	9.82	36.19	16.95	15.90	21.44	11.05
S24	QELISNAsDALDKIR	HSP90AB4P	Q58FF6	32.56	19.82	31.16	21.31	29.57	24.59	14.78	34.76	16.58	11.98	19.62	7.80

S109	RELISNAsDALDKIR	HSP90B1	P14625	32.56	19.82	31.16	21.31	29.57	24.59	14.78	34.76	16.58	11.98	19.62	7.80
S98	TADRWRVsLDVNHFA	HSPB1	P04792	31.41	21.26	33.09	16.80	28.33	24.59	13.00	34.42	17.50	11.46	20.37	10.07
S67	RTVIIEQsWGSPKVT	HSPD1	P10809	11.97	13.28	9.55	7.48	14.89	13.71	10.48	11.39	11.51	10.64	6.33	6.17
S423	AGCIHGLsNVKLNEH	IDH2	P48735	26.26	18.90	18.91	11.07	12.11	17.00	13.19	20.16	17.69	10.02	12.33	10.29
S258	EDLCKIGsERSLVLD	IKZF1	Q13422	23.55	25.75	16.11	12.70	21.97	21.67	12.72	19.24	11.79	10.64	13.29	11.81
S274	PPRTGGPsPAGPAAA	IRX5	P78411	39.77	42.82	35.02	31.04	32.03	29.80	32.93	25.06	23.58	23.96	23.80	25.24
S25	DLGHTPLsKKEGIKW	KCNIP3	Q9Y2W7	6.56	5.62	7.53	3.89	5.65	6.22	2.53	5.65	2.95	3.00	3.97	1.46
T713	RSCDEPLtPPPHSPT	KDM2A	Q9Y2K7	4.12	5.01	3.86	3.18	4.52	2.29	4.21	3.46	5.90	4.03	1.34	1.35
S731	QLIHDPVsPRGMVTR	KDM2A	Q9Y2K7	4.12	5.01	3.86	3.18	4.52	2.29	4.21	3.46	5.90	4.03	1.34	1.35
S570	PGASVSSsLTSLCSS	KIAA1522	Q9P206	17.12	10.32	9.17	12.60	8.93	7.95	7.39	7.51	7.00	8.68	9.54	6.28
S193	SLNNQFAsFIDKVRF	KRT1	P04264	117.13	59.98	89.72	55.43	79.05	81.82	43.69	106.47	60.43	46.16	63.68	28.27
S159	NLNDRLAsYLDKVRA	KRT10	P13645	73.75	31.17	53.54	27.77	42.09	47.26	16.19	72.81	29.57	18.38	39.67	13.87
S138	NLNDRLAsYLDKVRA	KRT12	Q99456	73.75	31.17	53.54	27.77	42.09	47.26	16.19	72.81	29.57	18.38	39.67	13.87
S128	NLNDRLAsYLDKVRA	KRT14	P02533	73.75	31.17	53.54	27.77	42.09	47.26	16.19	72.81	29.57	18.38	39.67	13.87
S118	NLNDRLAsYLDKVRA	KRT15	P19012	73.75	31.17	53.54	27.77	42.09	47.26	16.19	72.81	29.57	18.38	39.67	13.87
S130	NLNDRLAsYLDKVRA	KRT16	P08779	73.75	31.17	53.54	27.77	42.09	47.26	16.19	72.81	29.57	18.38	39.67	13.87
S97	NLNDRLAsYLDKVRA	KRT17	Q04695	73.75	31.17	53.54	27.77	42.09	47.26	16.19	72.81	29.57	18.38	39.67	13.87
T11	TTRSTFStNYRSLGS	KRT18	P05783	35.14	26.57	37.72	22.95	30.08	32.54	15.34	41.09	19.62	16.83	18.01	13.11
S319	SMRNLKAsLENSLRE	KRT18	P05783	40.67	44.55	28.56	19.36	31.00	34.10	20.68	39.32	23.30	19.73	25.19	12.89
S323	LKASLENsLREVEAR	KRT18	P05783	28.70	25.44	30.78	14.86	23.71	19.75	15.44	28.18	15.38	10.95	20.05	10.94
T425	VVSETNDtKVLR-	KRT18	P05783	27.93	16.96	18.52	16.80	19.92	18.65	7.67	24.72	13.36	10.74	12.54	7.80
S93	SLNDRLAsYLDRVRS	KRT18	P05783	89.71	35.36	62.90	28.89	54.10	51.47	26.38	68.68	35.19	24.48	31.95	12.89
S48	GGRGVSVsSARFVSS	KRT19	P08727	14.93	14.31	16.02	10.04	13.76	15.36	8.79	18.98	7.74	7.54	9.22	9.53
S93	NLNDRLAsYLDKVRA	KRT19	P08727	73.75	31.17	53.54	27.77	42.09	47.26	16.19	72.81	29.57	18.38	39.67	13.87

S191	TLNNKFAsFIDKVRF	KRT2	P35908	117.13	59.98	89.72	55.43	79.05	81.82	43.69	106.47	60.43	46.16	63.68	28.27
S211	TLNNKFAsFIDKVRF	KRT3	P12035	117.13	59.98	89.72	55.43	79.05	81.82	43.69	106.47	60.43	46.16	63.68	28.27
S181	TLNNKFAsFIDKVRF	KRT5	P13647	117.13	59.98	89.72	55.43	79.05	81.82	43.69	106.47	60.43	46.16	63.68	28.27
S176	TLNNKFAsFIDKVRF	KRT6A	P02538	117.13	59.98	89.72	55.43	79.05	81.82	43.69	106.47	60.43	46.16	63.68	28.27
S176	TLNNKFAsFIDKVRF	KRT6B	P04259	117.13	59.98	89.72	55.43	79.05	81.82	43.69	106.47	60.43	46.16	63.68	28.27
S176	TLNNKFAsFIDKVRF	KRT6C	P48668	117.13	59.98	89.72	55.43	79.05	81.82	43.69	106.47	60.43	46.16	63.68	28.27
S104	TLNNKFAsFIDKVRF	KRT7	P08729	117.13	59.98	89.72	55.43	79.05	81.82	43.69	106.47	60.43	46.16	63.68	28.27
S143	ALNNKFAsFIDKVRF	KRT71	Q3SY84	117.13	59.98	89.72	55.43	79.05	81.82	43.69	106.47	60.43	46.16	63.68	28.27
S138	ALNNKFAsFIDKVRF	KRT72	Q14CN4	117.13	59.98	89.72	55.43	79.05	81.82	43.69	106.47	60.43	46.16	63.68	28.27
S145	VLNNKFAsFIDKVRF	KRT73	Q86Y46	117.13	59.98	89.72	55.43	79.05	81.82	43.69	106.47	60.43	46.16	63.68	28.27
S153	VLNDKFAsFIDKVRF	KRT74	Q7RTS7	117.13	59.98	89.72	55.43	79.05	81.82	43.69	106.47	60.43	46.16	63.68	28.27
S162	TLNNKFAsFIDKVRF	KRT75	O95678	117.13	59.98	89.72	55.43	79.05	81.82	43.69	106.47	60.43	46.16	63.68	28.27
Y327	IAEVKAQyEDIANRS	KRT75	O95678	29.22	20.34	25.95	16.29	25.66	25.60	9.08	34.09	21.37	10.33	12.33	7.69
S196	TLNNKFAsFIDKVRF	KRT76	Q01546	117.13	59.98	89.72	55.43	79.05	81.82	43.69	106.47	60.43	46.16	63.68	28.27
S177	VLNNKFAsFIDKVRF	KRT77	Q7Z794	117.13	59.98	89.72	55.43	79.05	81.82	43.69	106.47	60.43	46.16	63.68	28.27
S124	TLNNQFAsFIDKVRF	KRT78	Q8N1N4	117.13	59.98	89.72	55.43	79.05	81.82	43.69	106.47	60.43	46.16	63.68	28.27
S155	TLNNKFAsFIDKVRF	KRT79	Q5XKE5	117.13	59.98	89.72	55.43	79.05	81.82	43.69	106.47	60.43	46.16	63.68	28.27
S104	TLNNKFAsFIDKVRF	KRT8	P05787	117.13	59.98	89.72	55.43	79.05	81.82	43.69	106.47	60.43	46.16	63.68	28.27
Y267	IAEVKAQyEDIANRS	KRT8	P05787	29.22	20.34	25.95	16.29	25.66	25.60	9.08	34.09	21.37	10.33	12.33	7.69
S291	IKYEELQsLAGKHGD	KRT8	P05787	15.32	9.50	14.09	12.50	12.22	12.43	8.51	20.08	12.16	7.54	9.76	5.52
S58	GGGYGGAsGMGGITA	KRT8	P05787	14.42	14.20	10.71	6.56	11.91	10.24	4.21	11.31	7.37	5.78	4.82	4.55
S178	TLNNKFAsFIDKVRF	KRT84	Q9NSB2	117.13	59.98	89.72	55.43	79.05	81.82	43.69	106.47	60.43	46.16	63.68	28.27
T865	TVGSYGCtPQSLPKF	LARP1	Q6PKG0	8.88	8.89	9.17	12.50	7.08	8.23	7.11	7.34	8.47	7.85	3.97	5.74
S144	LGFSSDEsDVEASPR	LEMD3	Q9Y2U8	66.54	74.09	48.14	46.00	58.62	59.88	34.52	59.90	34.27	28.71	35.17	35.64

S140	EGSEKAHsDDEKWGR	LEO1	Q8WVC0	13.13	12.57	13.51	11.78	14.06	13.53	6.74	5.82	7.09	10.74	5.25	5.52
S301	QSRIRIDsLSAQLSQ	LMNA	P02545	24.84	16.55	21.13	17.42	15.40	22.40	15.16	16.37	9.95	17.87	13.72	13.54
S1616	IQDRFLNsFEELQAE	LTBP1	Q14766	23.17	34.64	31.55	21.72	23.10	19.47	20.96	24.64	17.59	20.76	17.58	18.09
S214	SGSTYTPsEAGNELD	MAF1	Q9H063	8.69	12.77	8.59	9.78	8.01	8.41	10.85	8.69	9.40	6.30	5.63	7.64
S507	LNGERDGsLCQQQNE	MAP4K3	Q8IVH8	4.25	2.76	3.67	4.82	2.77	2.29	0.98	1.77	2.39	3.20	1.13	2.27
S410	HKVQRSVsSSQKQRR	MARK3	P27448	57.02	39.44	30.58	37.19	37.67	26.15	32.75	28.85	26.53	30.36	24.12	23.72
S1384	SPLARTPsPTPQPTS	MAST4	O15021	12.36	13.18	8.30	10.04	15.91	11.43	7.77	8.69	5.53	10.02	8.15	5.85
T1425	TSTDQPVtPEPTSQA	MDC1	Q14676	13.39	11.85	10.71	10.76	15.71	12.98	6.92	11.39	5.71	9.91	7.18	7.91
S1119	SPQVSGPsPAARMPG	MED14	O60244	16.09	12.77	15.15	12.91	10.88	13.62	8.51	21.09	11.97	7.02	11.26	6.17
Y47	VSGKRPDyAPMESSD	MFAP1	P55081	15.70	15.84	8.39	12.19	17.76	11.70	6.46	8.77	3.96	10.95	5.47	7.69
S1406	LSLPTPRsPSDRELR	MICAL3	Q7RTP6	10.43	5.72	9.36	7.68	7.39	3.11	6.36	5.32	5.90	4.65	5.15	6.17
S732	PPGPPNAsSNPDLRR	MINK1	Q8N4C8	41.06	81.65	33.86	43.44	36.34	58.78	21.61	60.58	25.33	28.61	25.19	29.68
T117	EVAATTAtPDGGPRA	NAA30	Q147X3	7.47	7.87	6.85	6.35	8.32	5.21	4.21	10.12	6.82	6.92	3.65	2.49
S145	APSSEPQsPVAQTSG	NACC1	Q96RE7	1.61	3.37	3.76	1.28	1.28	3.57	2.34	2.36	1.15	1.29	1.34	1.35
S282	RNLVYDQsPNRTGGP	NDE1	Q9NXR1	16.09	15.94	14.18	14.65	18.99	18.37	13.75	16.03	12.44	8.88	9.11	11.37
S106	TSLANLIsPVRNGAV	NET1	Q7Z628	1.48	2.86	2.89	1.18	3.80	1.05	1.08	1.94	1.06	1.19	1.23	1.25
S44	AMKFLRAsEEHLKQH	NME2	P22392	18.15	9.71	18.43	9.22	13.14	12.89	5.80	14.43	13.17	5.68	9.43	5.20
S29	AMKFLPAsEEHLKQH	NME2P1	O60361	18.15	9.71	18.43	9.22	13.14	12.89	5.80	14.43	13.17	5.68	9.43	5.20
S43	DENEHQLsLRTVSLG	NPM1	P06748	19.44	11.04	15.82	13.52	17.25	20.39	3.74	16.62	9.03	4.96	11.15	5.74
S948	RGDCSTNsPVGVSKV	NSD1	Q96L73	10.43	11.96	11.09	7.89	10.37	9.96	9.36	5.74	7.18	7.13	6.33	10.07
S259	SDRCALSsPSLAFTP	NUP35	Q8NFH5	74.78	79.35	66.47	52.46	62.00	69.48	40.32	69.10	45.09	42.24	43.53	43.76
T65	IITLAGPtNAIFKAF	PCBP2	Q15366	1.48	2.86	2.22	1.18	2.36	2.10	1.08	2.28	1.06	1.19	1.23	1.25
S232	NRYGMGTsVERAAAS	PDHA1	P08559	50.97	43.63	53.83	43.85	49.07	42.23	38.83	32.48	38.87	29.54	32.27	33.26
S415	LPGVDALsN-	PGK1	P00558	11.58	5.82	6.85	5.74	8.32	7.13	3.74	8.69	2.58	3.10	5.57	4.44

S259	NMTSGHSsAPPKETS	PM20D1	Q6GTS8	10.55	14.51	11.67	10.76	10.06	13.99	9.92	10.55	9.58	7.44	7.29	9.10
S512	GKIRRTQsGNFYTDT	PPFIBP2	Q8ND30	8.37	7.15	7.81	7.07	7.19	6.58	6.08	5.82	4.24	5.58	4.40	4.22
S150	AGKDTNGsQFFITTV	PPIB	P23284	11.33	11.34	14.28	9.12	13.24	9.51	9.73	11.81	7.46	4.85	8.47	5.31
T256	VAAPGDAtPPAEKKY	PPP1R10	Q96QC0	11.46	13.49	16.88	12.29	10.68	10.24	7.30	14.17	15.57	9.29	10.72	7.58
S437	RGAPHRHsMPELGLR	PRICKLE3	O43900	7.08	7.05	5.21	5.12	4.62	5.39	5.52	3.80	2.67	5.47	2.25	4.77
S34	GDGERRLsGSSLCSG	PRPF40A	O75400	7.08	10.93	8.10	7.79	9.65	8.23	7.48	9.36	7.83	4.85	6.11	5.96
S778	HNVFKRHsMREEDFI	PTK2B	Q14289	23.81	11.96	10.42	16.80	29.46	14.44	12.54	10.12	7.46	7.95	5.68	7.47
S255	LCAATGPsIKIWDLE	RACK1	P63244	3.73	2.35	5.50	4.92	3.39	3.47	1.08	5.06	3.04	1.19	1.23	1.25
S93	LDPAPAVsEAGPETH	RASEF	Q8IZ41	1.48	2.35	2.41	1.18	2.57	4.02	3.56	0.97	1.06	1.19	1.23	1.25
S78	RSEDGYHsDGDYGEH	RBM5	P52756	7.08	8.17	6.46	6.45	3.49	7.50	3.74	3.63	4.61	3.10	2.79	6.39
S120	SETVSEAsPGSTASQ	RFX1	P22670	8.75	7.66	6.13	4.35	6.06	6.03	7.44	7.76	5.43	4.91	4.34	4.50
S329	ATGEGGAsDLPEDPD	RNF113A	O15541	3.73	1.23	2.89	1.23	2.46	1.10	1.12	1.01	1.11	1.24	1.29	1.30
S77	VRAGRGFsLEELRVA	RPL13	P26373	23.04	16.45	20.94	15.88	19.71	19.65	16.65	22.36	17.32	12.19	14.47	10.07
S233	GITLLNVsKLNILKL	RPL4	P36578	3.09	2.86	2.22	1.08	2.16	2.47	0.98	3.71	0.97	1.08	1.13	1.14
S206	PRGTGIVsAPVPKKL	RPS2	P15880	4.63	3.88	6.27	4.30	3.18	1.83	2.25	3.97	3.22	2.58	1.07	1.08
S6	#NAME?	RPS6	P62753	4.89	4.29	6.85	4.41	4.41	7.04	3.46	7.59	3.50	1.08	2.25	2.38
S82	LLLSKGHsCYRPRRT	RPS6	P62753	22.27	15.33	22.38	14.65	21.66	21.21	10.57	20.08	17.04	8.78	14.15	10.62
S35	DGYNYTLsKTEFLSF	S100A11	P31949	38.36	30.15	36.18	31.45	28.95	25.78	26.01	39.40	23.95	22.51	24.87	17.77
S73	EKIANLGsCNDSKLE	S100A14	Q9HCY8	50.33	49.87	42.16	37.60	46.09	39.58	29.94	39.91	36.29	34.60	27.55	32.28
S138	SDDDGGDsPVQDIDT	SAP30	O75446	9.85	10.83	9.36	7.12	11.04	10.42	5.47	10.71	10.32	8.26	6.70	4.82
S1630	RGLGPVPs-	SCRIB	Q14160	5.02	2.15	3.18	6.04	3.49	3.20	4.30	3.71	3.13	2.58	1.13	1.14
T1185	APEPPPAtPPQAKFP	SETD1A	O15047	3.60	5.31	2.70	1.95	3.39	3.47	3.56	2.36	1.66	0.93	0.96	0.97
S45	CEERNLLsVAYKNVV	SFN	P31947	31.28	26.77	28.75	21.11	28.03	26.42	14.22	36.87	22.01	18.28	17.05	9.42
S63	RAAWRVLsSIEQKSN	SFN	P31947	45.31	30.15	34.35	26.95	33.16	35.84	18.62	42.35	20.73	21.07	27.45	18.20

S34	LPPPAPGsPAAPAAV	SIK3	Q9Y2K2	8.75	8.69	5.69	5.84	5.34	3.93	4.77	6.50	4.05	6.40	3.75	3.57
S2	#NAME?	SLC33A1	O00400	7.59	7.15	9.07	6.04	5.95	9.87	5.24	5.57	7.74	6.61	3.75	5.31
S216	KDLDLLAsVPSPSSS	SMAP2	Q8WU79	10.68	8.79	11.38	10.04	11.09	10.33	9.73	8.10	7.09	7.33	4.50	7.15
S451	PRAGPGSsPLFSLLP	SMG9	Q9H0W8	10.04	12.26	14.86	10.35	10.68	15.27	7.86	11.56	9.76	6.09	10.61	7.15
S258	PVPAVAHsPPATVEA	SOWAHB	A6NEL2	14.54	17.27	9.74	13.11	10.47	9.87	15.16	13.75	7.65	10.02	6.97	7.26
S180	KQGEVPEsPEARKES	SP140L	Q9H930	9.78	11.55	9.07	12.81	7.08	9.60	6.08	5.82	7.37	4.65	7.08	10.40
S1822	RGGSGYHsRSPARQE	SRRM2	Q9UQ35	7.08	5.62	4.73	5.63	3.70	5.58	4.30	4.39	3.32	4.44	3.54	2.82
S1824	GSGYHSRsPARQESS	SRRM2	Q9UQ35	7.08	5.62	4.73	5.63	3.70	5.58	4.30	4.39	3.32	4.44	3.54	2.82
S67	RNRRERFsPPRHELS	SRRT	Q9BXP5	4.12	3.58	4.15	3.79	2.87	4.39	3.37	2.19	2.21	2.48	1.29	2.82
S342	RRCTVDGsPHELESR	STRN4	Q9NRL3	19.95	17.58	14.57	13.63	14.68	16.27	17.03	16.87	12.53	11.98	13.19	8.45
S41	IKRHTPLsKLMKAYC	SUMO2	P61956	6.56	5.62	7.53	3.89	5.65	6.22	2.53	5.65	2.95	3.00	3.97	1.46
S40	IKRHTPLsKLMKAYC	SUMO3	P55854	6.56	5.62	7.53	3.89	5.65	6.22	2.53	5.65	2.95	3.00	3.97	1.46
S1039	NRGSRHSsAPPKKKR	SUPT16H	Q9Y5B9	10.55	14.51	11.67	10.76	10.06	13.99	9.92	10.55	9.58	7.44	7.29	9.10
S425	SSDDEVYsRPSSLVS	TBC1D9	Q6ZT07	27.03	26.67	35.60	27.05	25.25	25.96	22.55	27.17	28.65	15.70	19.62	19.61
S91	DPAGPAAsPVLADGL	TEX2	Q8IWB9	2.57	2.15	2.32	1.02	2.77	3.11	4.87	1.69	0.92	2.27	1.07	1.08
S224	PKDGSNKsGAEEQGP	TGOLN2	O43493	21.62	24.22	28.94	27.05	19.81	21.67	25.45	26.32	30.21	14.98	15.55	17.01
S266	PDYERAYsPEYRRGA	TJP2	Q9UDY2	174.27	171.98	162.66	166.59	119.29	122.13	85.89	98.88	71.48	90.68	89.74	104.43
S271	ARRDLVDsPASLASS	TLE2	Q04725	10.43	6.54	7.04	7.38	4.82	6.58	5.89	5.32	4.05	6.92	4.07	4.98
S393	FIRGVVDsEDIPLNL	TRAP1	Q12931	18.79	14.41	13.60	12.19	17.25	12.80	8.14	20.59	7.18	5.68	10.72	8.77
T109	NYARGHYtIGKEIID	TUBA1A	Q71U36	14.29	8.89	11.87	9.53	9.24	8.87	6.74	17.38	9.67	6.51	7.61	4.12
T223	NLDIERPtYTNLNRL	TUBA1A	Q71U36	23.30	19.52	19.78	14.55	15.50	20.29	11.98	23.54	15.66	10.22	14.69	9.42
S277	ATYAPVIsAEKAYHE	TUBA1A	Q71U36	10.17	10.42	14.47	8.40	11.91	10.88	8.33	16.11	7.83	6.82	6.75	3.79
T109	NYARGHYtIGKEIID	TUBA1B	P68363	14.29	8.89	11.87	9.53	9.24	8.87	6.74	17.38	9.67	6.51	7.61	4.12
T223	NLDIERPtYTNLNRL	TUBA1B	P68363	23.30	19.52	19.78	14.55	15.50	20.29	11.98	23.54	15.66	10.22	14.69	9.42

S277	ATYAPVIsAEKAYHE	TUBA1B	P68363	10.17	10.42	14.47	8.40	11.91	10.88	8.33	16.11	7.83	6.82	6.75	3.79
T109	NYARGHYtIGKEIID	TUBA1C	Q9BQE3	14.29	8.89	11.87	9.53	9.24	8.87	6.74	17.38	9.67	6.51	7.61	4.12
T223	NLDIERPtYTNLNRL	TUBA1C	Q9BQE3	23.30	19.52	19.78	14.55	15.50	20.29	11.98	23.54	15.66	10.22	14.69	9.42
S277	ATYAPVIsAEKAYHE	TUBA1C	Q9BQE3	10.17	10.42	14.47	8.40	11.91	10.88	8.33	16.11	7.83	6.82	6.75	3.79
T109	NYARGHYtIGKEIVD	TUBA3C	P0DPH7	14.29	8.89	11.87	9.53	9.24	8.87	6.74	17.38	9.67	6.51	7.61	4.12
T223	NLDIERPtYTNLNRL	TUBA3C	P0DPH7	23.30	19.52	19.78	14.55	15.50	20.29	11.98	23.54	15.66	10.22	14.69	9.42
S277	ATYAPVIsAEKAYHE	TUBA3C	P0DPH7	10.17	10.42	14.47	8.40	11.91	10.88	8.33	16.11	7.83	6.82	6.75	3.79
T109	NYARGHYtIGKEIVD	TUBA3D	P0DPH8	14.29	8.89	11.87	9.53	9.24	8.87	6.74	17.38	9.67	6.51	7.61	4.12
T223	NLDIERPtYTNLNRL	TUBA3D	P0DPH8	23.30	19.52	19.78	14.55	15.50	20.29	11.98	23.54	15.66	10.22	14.69	9.42
S277	ATYAPVIsAEKAYHE	TUBA3D	P0DPH8	10.17	10.42	14.47	8.40	11.91	10.88	8.33	16.11	7.83	6.82	6.75	3.79
T109	NYARGHYtIGKEIVD	TUBA3E	Q6PEY2	14.29	8.89	11.87	9.53	9.24	8.87	6.74	17.38	9.67	6.51	7.61	4.12
T223	NLDIERPtYTNLNRL	TUBA3E	Q6PEY2	23.30	19.52	19.78	14.55	15.50	20.29	11.98	23.54	15.66	10.22	14.69	9.42
S277	ATYAPVIsAEKAYHE	TUBA3E	Q6PEY2	10.17	10.42	14.47	8.40	11.91	10.88	8.33	16.11	7.83	6.82	6.75	3.79
T109	NYARGHYtIGKEIID	TUBA4A	P68366	14.29	8.89	11.87	9.53	9.24	8.87	6.74	17.38	9.67	6.51	7.61	4.12
T223	NLDIERPtYTNLNRL	TUBA4A	P68366	23.30	19.52	19.78	14.55	15.50	20.29	11.98	23.54	15.66	10.22	14.69	9.42
S277	ATYAPVIsAEKAYHE	TUBA4A	P68366	10.17	10.42	14.47	8.40	11.91	10.88	8.33	16.11	7.83	6.82	6.75	3.79
T162	NLDIERPtYTNLNRL	TUBA4B	Q9H853	23.30	19.52	19.78	14.55	15.50	20.29	11.98	23.54	15.66	10.22	14.69	9.42
T48	NYAWGHYtIGKEFID	TUBA4B	Q9H853	14.29	8.89	11.87	9.53	9.24	8.87	6.74	17.38	9.67	6.51	7.61	4.12
T223	NLDIERPtYTNLNRL	TUBA8	Q9NY65	23.30	19.52	19.78	14.55	15.50	20.29	11.98	23.54	15.66	10.22	14.69	9.42
S48	DLQLDRIsVYYNEAT	TUBB	P07437	6.95	4.80	9.45	6.76	11.19	9.78	4.77	7.76	5.62	2.17	4.61	4.44
S288	LIDASEDsQLEAAIR	UBXN7	O94888	9.40	11.14	6.85	7.89	7.08	6.76	8.98	6.50	6.08	4.96	7.61	5.31
Y541	HKVRGYRyLEEDNSD	UNC93B1	Q9H1C4	4.38	4.91	2.60	5.12	0.92	3.11	3.37	3.21	3.68	3.00	0.96	1.95
S680	VNEKTIGsPPNEFYC	USP38	Q8NB14	10.17	9.40	11.19	9.02	7.39	6.49	4.21	7.68	6.54	6.40	8.47	4.98
S407	EAHGVSEsEGEERPV	UTP14A	Q9BVJ6	8.37	8.69	7.62	6.04	8.32	7.59	8.79	7.85	4.15	4.65	5.36	5.85

S569	QNLLTTQsPSVKSLA	UTP14A	Q9BVJ6	5.41	6.03	6.37	4.71	4.52	4.75	3.46	4.22	3.78	3.30	4.50	4.22
S356	EDFSPFGsGGGLFSG	WASHC2A	Q641Q2	5.02	4.70	5.50	4.92	4.72	4.85	3.18	2.53	2.39	3.10	2.57	3.90
S356	EDFSPFGsGGGLFSG	WASHC2C	Q9Y4E1	5.02	4.70	5.50	4.92	4.72	4.85	3.18	2.53	2.39	3.10	2.57	3.90
S544	LFSDEEDsEDLFSSQ	WASHC2C	Q9Y4E1	1.48	2.66	2.80	3.28	1.18	2.10	2.43	0.97	1.06	1.19	1.23	1.25
S47	NEERNLLsVAYKNVV	YWHAB	P31946	31.28	26.77	28.75	21.11	28.03	26.42	14.22	36.87	22.01	18.28	17.05	9.42
S46	VEERNLLsVAYKNVI	YWHAE	P62258	31.28	26.77	28.75	21.11	28.03	26.42	14.22	36.87	22.01	18.28	17.05	9.42
S46	NEERNLLsVAYKNVV	YWHAG	P61981	31.28	26.77	28.75	21.11	28.03	26.42	14.22	36.87	22.01	18.28	17.05	9.42
S46	NEDRNLLsVAYKNVV	YWHAH	Q04917	31.28	26.77	28.75	21.11	28.03	26.42	14.22	36.87	22.01	18.28	17.05	9.42
S45	NEERNLLsVAYKNVV	YWHAQ	P27348	31.28	26.77	28.75	21.11	28.03	26.42	14.22	36.87	22.01	18.28	17.05	9.42
S114	PNASQAEsKVFYLKM	YWHAZ	P63104	15.45	10.01	19.30	7.48	11.39	11.88	7.77	16.87	7.65	8.06	11.26	5.85
S156	YQEAFEIsKKEMQPT	YWHAZ	P63104	14.29	14.82	13.02	14.96	16.43	13.80	8.51	20.08	11.61	8.68	12.44	5.52
S45	NEERNLLsVAYKNVV	YWHAZ	P63104	31.28	26.77	28.75	21.11	28.03	26.42	14.22	36.87	22.01	18.28	17.05	9.42
S63	RSSWRVVsSIEQKTE	YWHAZ	P63104	35.78	22.07	28.75	19.47	29.15	28.07	15.72	33.07	21.00	18.28	22.62	15.06
S345	SLLRRSLsMDSQVPV	ZBTB21	Q9ULJ3	29.86	32.70	21.42	18.34	25.36	34.83	12.26	23.88	13.26	12.70	12.01	13.54
S1182	GEEEAPPsRSDPDGG	ZNF687	Q8N1G0	6.44	4.91	4.53	4.61	3.29	4.48	3.84	4.98	4.88	2.89	4.82	2.38
S1184	EEAPPSRsDPDGGDS	ZNF687	Q8N1G0	6.44	4.91	4.53	4.61	3.29	4.48	3.84	4.98	4.88	2.89	4.82	2.38
Y483	IDIRPRPyTCSECGK	ZNF749	O43361	9.27	9.30	8.97	10.55	10.37	8.78	6.08	9.28	8.47	4.65	5.90	7.15
T484	DIRPRPYtCSECGKA	ZNF749	O43361	9.27	9.30	8.97	10.55	10.37	8.78	6.08	9.28	8.47	4.65	5.90	7.15
S82	PAAPRSRsLGGAVGS	ZNRF2	Q8NHG8	66.93	73.27	59.04	57.99	53.07	59.42	41.73	47.42	38.23	41.83	42.99	42.14
**0.1 µM ENDX:**															
S1410	PQDPDNVsLQEVEAE	ABCA2	Q9BZC7	1.29	1.02	0.96	2.05	1.03	0.91	2.34	3.21	2.39	1.03	2.25	2.17
S404	EKFRQKAsIHEAWTD	ACTN1	P12814	13.13	13.18	18.52	18.34	17.55	21.30	22.64	25.31	24.87	23.86	24.98	22.32
S423	EKFRQKAsIHEAWTD	ACTN4	O43707	13.13	13.18	18.52	18.34	17.55	21.30	22.64	25.31	24.87	23.86	24.98	22.32
T217	RSQSAAVtPSSTTSS	ADRM1	Q16186	8.37	9.91	6.95	10.76	8.93	15.45	11.13	10.63	8.38	11.98	13.40	13.97

S62	TLPSIWDsPTKQLSV	ALG5	Q9Y673	4.38	5.42	5.69	4.92	7.90	5.58	8.05	7.17	11.15	7.44	5.15	6.61
S100	PPLQSARsLPGPAPC	AMPD2	Q01433	4.76	3.47	2.70	6.86	5.95	3.11	4.87	4.81	4.33	6.71	5.68	5.42
S669	EAEGAPGsPEREPPA	ANO8	Q9HCE9	1.29	2.04	0.96	2.56	2.05	1.92	3.27	4.13	2.95	3.41	3.00	2.60
S96	QQQGEDGsPQAHRRG	AR	P10275	1.29	1.02	0.96	1.02	1.03	2.29	2.90	3.12	0.92	2.89	2.14	2.17
S592	IEEEIPDsPGIEKHD	ARHGAP12	Q8IWW6	1.48	5.31	2.22	3.48	5.34	3.38	5.80	5.32	5.71	4.54	6.22	4.87
S227	QERGLNTsQESDDDI	ARHGEF16	Q5VV41	7.98	6.95	9.26	9.94	8.32	8.87	14.31	8.61	14.37	11.88	12.65	9.32
S413	DDRPSCPs-	ARRDC3	Q96B67	1.29	1.02	0.96	1.02	2.05	3.20	0.94	2.02	2.86	3.30	2.47	2.60
S728	AMPIPAAsPTPASPA	ATXN2	Q99700	13.71	9.81	12.98	17.83	13.96	15.72	19.41	15.40	14.74	18.54	19.89	16.90
S733	AASPTPAsPASNRAV	ATXN2	Q99700	1.35	1.07	1.01	3.18	3.39	3.47	0.98	2.95	2.21	2.17	4.61	2.38
S134	LYQVPGPsPQFQSPP	BCAR1	P56945	4.25	3.27	6.46	6.45	6.06	9.69	6.36	9.20	9.40	6.51	6.97	5.20
S165	GAELEYDsEHSDWHG	C20orf27	Q9GZN8	5.41	7.15	8.88	10.04	12.22	12.16	10.67	10.21	8.38	11.77	7.40	12.46
S168	LEYDSEHsDWHGF-	C20orf27	Q9GZN8	5.41	7.15	8.88	10.04	12.22	12.16	10.67	10.21	8.38	11.77	7.40	12.46
S643	ISEEIIGsPIPEPRQ	C2CD5	Q86YS7	1.54	2.45	3.38	4.00	4.52	3.11	4.12	4.89	4.70	4.13	2.68	4.55
S280	SGSSGCPsPTPQSSD	CBX6	O95503	2.45	5.42	4.44	3.89	5.34	6.49	5.33	4.98	7.74	8.88	6.00	7.26
S240	AQRQRAHsVDVEKNR	CCSAP	Q6IQ19	5.66	5.11	7.53	8.81	6.98	5.85	12.35	7.09	9.21	11.67	6.22	6.61
T177	KPSRDPEtPRSSGSM	CDCA3	Q99618	4.12	4.09	7.81	9.22	4.62	9.60	10.95	6.58	9.86	8.06	6.86	10.94
S793	DVAPTLMsVPRYLPR	CDH1	P12830	1.42	4.50	2.60	5.12	2.26	4.66	6.64	4.13	3.68	4.34	5.47	6.61
S12	KKIKRQLsMTLRGGR	CDK16	Q00536	6.69	7.05	4.82	7.68	6.06	7.50	8.70	7.26	8.01	10.33	8.36	9.86
T417	PEPVSSPtTPPEYKA	CDR2	Q01850	1.29	1.02	1.93	3.38	2.57	2.56	2.25	2.28	5.71	3.72	3.32	1.08
T418	EPVSSPTtPPEYKAL	CDR2	Q01850	1.29	1.02	1.93	3.38	2.57	2.56	2.25	2.28	5.71	3.72	3.32	1.08
S164	NENQLLNsQD-	CGGBP1	Q9UFW8	1.48	2.35	3.09	6.15	4.31	4.48	3.93	5.32	5.16	4.03	3.43	4.66
S756	GDSGSLFsPSKEEAR	CPSF1	Q10570	4.25	2.35	6.08	3.79	6.57	7.77	6.36	6.83	11.51	7.33	8.15	5.31
S533	PPLSTERsPEAVGSE	CRYBG1	Q9Y4K1	2.70	2.15	2.60	3.28	4.31	4.30	2.81	4.81	4.15	4.65	3.00	2.71
S320	DPRRRLRsYEDMIGE	CTNND1	O60716	2.70	3.17	3.76	3.18	4.11	5.12	5.24	5.06	4.88	3.30	5.47	3.68

S560	GKVSSPLsPLSPGIK	CTTNBP2NL	Q9P2B4	5.92	3.99	5.60	7.58	8.42	7.04	8.79	7.85	6.91	10.43	9.11	4.87
S381	KVELQHIsQKDCQED	DBF4	Q9UBU7	5.15	6.74	4.24	8.61	7.60	5.12	6.36	7.00	5.25	7.44	9.76	7.47
S413	EKKLLFIsEPIPHPS	DBF4	Q9UBU7	3.35	2.25	4.82	5.63	4.93	5.85	4.68	4.89	5.34	4.13	4.61	5.96
S420	SEPIPHPsNELRGLN	DBF4	Q9UBU7	3.35	2.25	4.82	5.63	4.93	5.85	4.68	4.89	5.34	4.13	4.61	5.96
S287	EAGAETRsPGKAEAE	DDX24	Q9GZR7	1.48	1.18	1.11	1.18	2.77	1.05	2.15	4.30	2.39	3.51	1.23	1.25
S109	FHSKPVDsDSDDDPL	DDX42	Q86XP3	29.73	21.66	37.14	35.81	33.06	34.28	35.27	31.81	39.06	48.33	41.71	45.93
S111	SKPVDSDsDDDPLEA	DDX42	Q86XP3	29.73	21.66	37.14	35.81	33.06	34.28	35.27	31.81	39.06	48.33	41.71	45.93
S160	KADSEPEsPLNASYV	DDX59	Q5T1V6	13.77	9.91	15.05	19.77	21.04	21.03	14.88	15.02	16.86	20.04	14.37	17.87
S265	STSFMSVsPSKEIKI	DEPTOR	Q8TB45	23.17	22.28	25.57	35.14	28.44	36.11	31.53	27.34	30.58	40.17	34.63	41.38
S460	TKQSMDMsPIKIVKN	DHX8	Q14562	54.96	30.96	65.41	77.25	54.61	64.72	72.70	63.28	77.10	84.07	71.72	77.78
S454	NSRKRREsASSSSSV	DMAP1	Q9NPF5	3.22	4.19	3.67	5.33	6.98	4.85	9.73	5.23	6.17	7.75	7.29	3.68
S370	ENPLDDNsEEEMEDA	DNAJC21	Q5F1R6	59.46	30.25	63.19	68.85	50.30	65.00	76.06	67.33	72.22	80.35	71.08	89.37
S196	RKEDRSAsSGAEGDV	DTD1	Q8TEA8	3.86	5.82	4.15	8.09	4.72	5.67	7.58	7.34	7.37	5.37	7.40	6.28
T55	TNTERPDtPTNTPNA	EED	O75530	3.93	3.63	7.24	7.07	5.18	7.86	7.02	9.28	9.58	5.89	5.25	6.39
S1194	SKEVEERsRERPSQP	EIF4G1	Q04637	9.01	5.82	7.91	10.96	10.88	10.97	11.79	8.86	13.91	10.64	10.08	9.86
S66	FREVVSFsPDPLPVR	ENGASE	Q8NFI3	1.42	2.55	1.06	4.10	2.57	3.38	2.53	3.80	3.68	2.27	3.43	1.19
T148	HFRFPPStPSEVLSP	ERF	P50548	2.51	1.99	3.71	3.89	3.29	2.93	3.23	2.45	3.68	3.61	4.72	5.74
S150	RFPPSTPsEVLSPTE	ERF	P50548	2.51	1.99	3.71	3.89	3.29	2.93	3.23	2.45	3.68	3.61	4.72	5.74
S161	SPTEDPRsPPACSSS	ERF	P50548	2.51	1.99	3.71	3.89	3.29	2.93	3.23	2.45	3.68	3.61	4.72	5.74
S369	SRMKPAGsVNDMALD	EVL	Q9UI08	4.63	6.34	6.08	6.04	8.32	8.32	9.36	9.03	8.11	4.44	9.11	9.32
S270	TTTDSPVsPAQAASP	FAM122A	Q96E09	4.18	6.85	8.20	8.61	7.49	6.49	8.61	7.00	9.03	9.71	10.40	10.72
S276	VSPAQAAsPFIPLDE	FAM122A	Q96E09	2.90	4.80	4.44	6.15	5.34	4.39	5.15	3.80	4.88	6.09	7.08	5.09
S436	VSRQTFLsHGDDFRF	FAM83H	Q6ZRV2	8.37	5.42	4.53	9.53	8.52	12.43	11.51	7.09	7.92	7.02	10.51	10.29
S461	VGVQSCPsPFSGQAC	FBXL18	Q96ME1	1.29	2.96	0.96	2.25	5.13	4.48	3.65	5.57	5.07	3.10	2.14	6.50

S135	DSLLFDTsDDEELRE	FEZ2	Q9UHY8	1.29	1.02	2.41	1.02	2.87	3.20	0.94	2.19	2.03	2.07	3.22	3.03
S2107	TRTSRAPsVATVGSI	FLNB	O75369	8.37	10.73	13.60	16.39	17.76	19.56	21.61	16.79	19.62	18.07	25.62	21.99
S13	PPPQDYEsDDDSYEV	FUNDC1	Q8IVP5	2.83	3.58	2.22	5.12	4.52	2.65	3.18	4.81	5.16	3.92	3.54	4.66
S23	SPPPKARsNENLDKI	FYTTD1	Q96QD9	9.78	15.74	12.16	20.39	13.35	15.08	18.06	18.81	15.84	20.04	18.65	18.20
S10	WMFKEDHsLEHRCVE	GABARAPL2	P60520	2.96	3.78	4.15	5.23	7.19	6.31	5.89	4.64	5.71	7.44	7.50	6.82
S620	GPLDAPGsPLACTEP	GAS2L1	Q99501	3.35	4.50	4.05	6.97	5.95	5.21	4.30	5.57	8.29	4.96	5.57	6.82
S508	RRDRQAFsMYEPGSA	GIT1	Q9Y2X7	11.71	7.66	9.07	13.01	13.55	10.15	17.31	10.46	12.71	15.70	15.22	13.76
S9	SGRPRTTsFAESCKP	GSK3B	P49841	27.16	34.23	28.07	39.44	30.39	36.84	41.26	47.84	37.77	46.58	40.53	48.42
T427	AKRLRLDtGPQSLSG	GTF2F1	P35269	11.97	13.28	11.29	11.78	17.25	15.08	18.99	21.01	18.05	13.84	18.01	19.17
S784	GVPFRRPsTFGIPRL	GTF2I	P78347	2.70	3.37	4.44	2.25	5.95	4.94	5.05	4.56	7.18	5.78	5.04	7.91
S454	VERTRKRsEGFSMDR	HDGFL2	Q7Z4V5	19.44	17.47	21.03	21.82	23.20	26.51	30.31	29.19	28.37	24.79	24.77	30.66
S1003	LLPPEPPsPARAPVP	INPPL1	O15357	1.93	3.37	4.44	7.17	4.00	5.76	4.40	5.91	4.24	4.03	5.47	3.25
T33	PGPSGSEtPPPPRRA	ITPKB	P27987	5.92	5.11	5.02	6.35	9.85	7.13	7.30	8.44	9.03	7.54	6.65	5.52
T104	METGIAEtPEGRRTS	KDM1A	O60341	1.16	0.92	2.51	3.79	1.85	3.66	3.09	2.36	3.50	3.82	0.96	3.03
S17	KKASFDHsPDSLPLR	KDM1B	Q8NB78	17.38	17.17	27.11	25.10	17.66	23.86	26.85	24.55	28.55	30.05	28.30	32.71
S730	GRSSSPTsSLTQPIE	KDM3B	Q7LBC6	3.99	6.03	6.56	7.07	5.85	8.96	9.26	10.71	7.18	7.33	5.79	6.39
T259	GTRAPPLtRPMSLAV	KIAA1522	Q9P206	2.00	1.58	2.99	3.89	5.54	3.11	4.30	2.78	4.15	3.82	4.07	3.79
S177	TELAMRQsVENDIHG	KRT18	P05783	1.35	1.07	2.60	2.15	4.21	0.96	2.34	4.72	3.87	2.58	2.68	3.03
S64	SASERLPsVEEAEVP	LAD1	O00515	5.28	6.23	7.53	10.45	6.06	7.59	10.85	11.64	10.32	5.78	6.97	11.16
S324	DSGDKRWsGNEPTDE	LRCH3	Q96II8	2.96	2.04	4.05	3.89	6.88	4.66	4.49	6.16	3.41	2.48	6.22	5.42
S592	SLRCGEAsPPSAACG	MAP1S	Q66K74	1.87	1.48	1.40	1.49	1.49	3.75	4.58	3.71	2.86	1.50	3.54	3.14
S696	PNKELPPsPEKKTKP	MAP4	P27816	8.37	6.95	13.89	11.27	9.65	10.79	14.50	12.82	16.21	13.32	14.90	10.29
S42	HDSERAVsADPLPPP	MARVELD2	Q8N4S9	1.35	2.15	2.80	3.18	3.29	3.38	4.49	4.05	3.78	2.79	4.07	2.60
T474	KPQKRPLtPFHHRVS	MED13	Q9UHV7	4.89	2.86	4.34	5.94	4.62	6.40	7.20	5.23	6.82	6.71	4.50	5.63

T821	PVILEPEtEESENEF	MIS18BP1	Q6P0N0	3.35	3.07	2.41	6.04	2.57	2.29	3.84	3.04	3.87	5.58	4.93	4.55
S824	LEPETEEsENEFYIK	MIS18BP1	Q6P0N0	3.35	3.07	2.41	6.04	2.57	2.29	3.84	3.04	3.87	5.58	4.93	4.55
S357	QYSQQQNsPQKHKNK	MKI67	P46013	3.09	4.60	6.37	7.68	6.88	7.50	6.36	7.51	7.92	5.68	5.90	4.66
S649	EVIEVDEsDVEEDIF	MRE11	P49959	2.70	2.15	2.03	3.28	4.41	5.03	6.27	3.21	1.93	2.17	4.50	2.27
S1307	PSEDRRWsTELVPEG	MYO9A	B2RTY4	3.86	4.50	7.14	7.89	6.36	8.41	7.30	6.58	10.04	7.95	9.43	10.51
S1317	LVPEGLQsPRGTPDS	MYO9A	B2RTY4	7.34	9.40	9.84	11.58	11.09	11.06	9.82	10.71	13.45	13.32	13.83	13.87
S69	TPTGYIEsLPRVVKR	NAP1L1	P55209	1.54	1.23	2.32	3.07	2.77	3.38	3.84	3.37	2.76	2.58	5.47	3.25
T1867	NLGEVPLtPTEEASL	NBEAL2	Q6ZNJ1	7.59	5.72	9.17	7.68	9.03	7.04	12.07	9.53	13.08	9.81	9.43	11.70
S674	CEGTEINsDDEQESK	NCAPG	Q9BPX3	6.69	5.52	6.66	9.02	8.52	7.40	11.88	9.96	8.66	8.78	9.97	6.93
S1299	SLSDRANsTESVRNT	NEO1	Q92859	10.55	12.57	13.12	13.22	13.35	12.52	16.93	18.56	18.88	15.80	15.33	13.43
S53	PNAHKVAsPPSGPAY	NFATC2	Q13469	7.34	11.60	8.54	9.48	12.01	13.35	11.04	12.66	13.45	16.01	11.63	14.84
S295	DFYPSPSsPAAGSRT	NFIB	O00712	2.45	0.97	0.92	5.94	4.93	4.39	2.62	4.56	3.59	5.27	4.07	2.92
T709	FFQPPPLtPTSKVYT	OGA	O60502	6.05	6.03	5.02	7.38	6.47	7.04	7.30	8.35	8.75	9.50	9.97	7.04
S203	VSRQRVEsLRKKRPL	PANK2	Q9BZ23	7.85	3.07	4.24	7.79	4.82	4.57	11.51	8.86	6.63	8.78	8.79	7.26
T883	ARYGPQFtLQHVPDY	PCDHGA1	Q9Y5H4	6.44	6.03	7.43	8.50	12.42	9.51	11.88	10.46	10.59	11.77	10.19	9.10
S899	QNVYIPGsNATLTNA	PCDHGA1	Q9Y5H4	17.50	16.35	18.81	21.21	21.46	25.60	23.95	23.45	20.36	25.92	33.66	22.10
T888	ARYGPQFtLQHVPDY	PCDHGA10	Q9Y5H3	6.44	6.03	7.43	8.50	12.42	9.51	11.88	10.46	10.59	11.77	10.19	9.10
S904	QNVYIPGsNATLTNA	PCDHGA10	Q9Y5H3	17.50	16.35	18.81	21.21	21.46	25.60	23.95	23.45	20.36	25.92	33.66	22.10
T887	ARYGPQFtLQHVPDY	PCDHGA11	Q9Y5H2	6.44	6.03	7.43	8.50	12.42	9.51	11.88	10.46	10.59	11.77	10.19	9.10
S903	QNVYIPGsNATLTNA	PCDHGA11	Q9Y5H2	17.50	16.35	18.81	21.21	21.46	25.60	23.95	23.45	20.36	25.92	33.66	22.10
T884	ARYGPQFtLQHVPDY	PCDHGA12	O60330	6.44	6.03	7.43	8.50	12.42	9.51	11.88	10.46	10.59	11.77	10.19	9.10
S900	QNVYIPGsNATLTNA	PCDHGA12	O60330	17.50	16.35	18.81	21.21	21.46	25.60	23.95	23.45	20.36	25.92	33.66	22.10
T884	ARYGPQFtLQHVPDY	PCDHGA2	Q9Y5H1	6.44	6.03	7.43	8.50	12.42	9.51	11.88	10.46	10.59	11.77	10.19	9.10
S900	QNVYIPGsNATLTNA	PCDHGA2	Q9Y5H1	17.50	16.35	18.81	21.21	21.46	25.60	23.95	23.45	20.36	25.92	33.66	22.10

T884	ARYGPQFtLQHVPDY	PCDHGA3	Q9Y5H0	6.44	6.03	7.43	8.50	12.42	9.51	11.88	10.46	10.59	11.77	10.19	9.10
S900	QNVYIPGsNATLTNA	PCDHGA3	Q9Y5H0	17.50	16.35	18.81	21.21	21.46	25.60	23.95	23.45	20.36	25.92	33.66	22.10
T914	ARYGPQFtLQHVPDY	PCDHGA4	Q9Y5G9	6.44	6.03	7.43	8.50	12.42	9.51	11.88	10.46	10.59	11.77	10.19	9.10
S930	QNVYIPGsNATLTNA	PCDHGA4	Q9Y5G9	17.50	16.35	18.81	21.21	21.46	25.60	23.95	23.45	20.36	25.92	33.66	22.10
T883	ARYGPQFtLQHVPDY	PCDHGA5	Q9Y5G8	6.44	6.03	7.43	8.50	12.42	9.51	11.88	10.46	10.59	11.77	10.19	9.10
S899	QNVYIPGsNATLTNA	PCDHGA5	Q9Y5G8	17.50	16.35	18.81	21.21	21.46	25.60	23.95	23.45	20.36	25.92	33.66	22.10
T884	ARYGPQFtLQHVPDY	PCDHGA6	Q9Y5G7	6.44	6.03	7.43	8.50	12.42	9.51	11.88	10.46	10.59	11.77	10.19	9.10
S900	QNVYIPGsNATLTNA	PCDHGA6	Q9Y5G7	17.50	16.35	18.81	21.21	21.46	25.60	23.95	23.45	20.36	25.92	33.66	22.10
T884	ARYGPQFtLQHVPDY	PCDHGA7	Q9Y5G6	6.44	6.03	7.43	8.50	12.42	9.51	11.88	10.46	10.59	11.77	10.19	9.10
S900	QNVYIPGsNATLTNA	PCDHGA7	Q9Y5G6	17.50	16.35	18.81	21.21	21.46	25.60	23.95	23.45	20.36	25.92	33.66	22.10
T884	ARYGPQFtLQHVPDY	PCDHGA8	Q9Y5G5	6.44	6.03	7.43	8.50	12.42	9.51	11.88	10.46	10.59	11.77	10.19	9.10
S900	QNVYIPGsNATLTNA	PCDHGA8	Q9Y5G5	17.50	16.35	18.81	21.21	21.46	25.60	23.95	23.45	20.36	25.92	33.66	22.10
T884	ARYGPQFtLQHVPDY	PCDHGA9	Q9Y5G4	6.44	6.03	7.43	8.50	12.42	9.51	11.88	10.46	10.59	11.77	10.19	9.10
S900	QNVYIPGsNATLTNA	PCDHGA9	Q9Y5G4	17.50	16.35	18.81	21.21	21.46	25.60	23.95	23.45	20.36	25.92	33.66	22.10
T879	ARYGPQFtLQHVPDY	PCDHGB1	Q9Y5G3	6.44	6.03	7.43	8.50	12.42	9.51	11.88	10.46	10.59	11.77	10.19	9.10
S895	QNVYIPGsNATLTNA	PCDHGB1	Q9Y5G3	17.50	16.35	18.81	21.21	21.46	25.60	23.95	23.45	20.36	25.92	33.66	22.10
T883	ARYGPQFtLQHVPDY	PCDHGB2	Q9Y5G2	6.44	6.03	7.43	8.50	12.42	9.51	11.88	10.46	10.59	11.77	10.19	9.10
S899	QNVYIPGsNATLTNA	PCDHGB2	Q9Y5G2	17.50	16.35	18.81	21.21	21.46	25.60	23.95	23.45	20.36	25.92	33.66	22.10
T881	ARYGPQFtLQHVPDY	PCDHGB3	Q9Y5G1	6.44	6.03	7.43	8.50	12.42	9.51	11.88	10.46	10.59	11.77	10.19	9.10
S897	QNVYIPGsNATLTNA	PCDHGB3	Q9Y5G1	17.50	16.35	18.81	21.21	21.46	25.60	23.95	23.45	20.36	25.92	33.66	22.10
T875	ARYGPQFtLQHVPDY	PCDHGB4	Q9UN71	6.44	6.03	7.43	8.50	12.42	9.51	11.88	10.46	10.59	11.77	10.19	9.10
S891	QNVYIPGsNATLTNA	PCDHGB4	Q9UN71	17.50	16.35	18.81	21.21	21.46	25.60	23.95	23.45	20.36	25.92	33.66	22.10
T875	ARYGPQFtLQHVPDY	PCDHGB5	Q9Y5G0	6.44	6.03	7.43	8.50	12.42	9.51	11.88	10.46	10.59	11.77	10.19	9.10
S891	QNVYIPGsNATLTNA	PCDHGB5	Q9Y5G0	17.50	16.35	18.81	21.21	21.46	25.60	23.95	23.45	20.36	25.92	33.66	22.10

T882	ARYGPQFtLQHVPDY	PCDHGB6	Q9Y5F9	6.44	6.03	7.43	8.50	12.42	9.51	11.88	10.46	10.59	11.77	10.19	9.10
S898	QNVYIPGsNATLTNA	PCDHGB6	Q9Y5F9	17.50	16.35	18.81	21.21	21.46	25.60	23.95	23.45	20.36	25.92	33.66	22.10
T881	ARYGPQFtLQHVPDY	PCDHGB7	Q9Y5F8	6.44	6.03	7.43	8.50	12.42	9.51	11.88	10.46	10.59	11.77	10.19	9.10
S897	QNVYIPGsNATLTNA	PCDHGB7	Q9Y5F8	17.50	16.35	18.81	21.21	21.46	25.60	23.95	23.45	20.36	25.92	33.66	22.10
T886	ARYGPQFtLQHVPDY	PCDHGC3	Q9UN70	6.44	6.03	7.43	8.50	12.42	9.51	11.88	10.46	10.59	11.77	10.19	9.10
S902	QNVYIPGsNATLTNA	PCDHGC3	Q9UN70	17.50	16.35	18.81	21.21	21.46	25.60	23.95	23.45	20.36	25.92	33.66	22.10
T890	ARYGPQFtLQHVPDY	PCDHGC4	Q9Y5F7	6.44	6.03	7.43	8.50	12.42	9.51	11.88	10.46	10.59	11.77	10.19	9.10
S906	QNVYIPGsNATLTNA	PCDHGC4	Q9Y5F7	17.50	16.35	18.81	21.21	21.46	25.60	23.95	23.45	20.36	25.92	33.66	22.10
T896	ARYGPQFtLQHVPDY	PCDHGC5	Q9Y5F6	6.44	6.03	7.43	8.50	12.42	9.51	11.88	10.46	10.59	11.77	10.19	9.10
S912	QNVYIPGsNATLTNA	PCDHGC5	Q9Y5F6	17.50	16.35	18.81	21.21	21.46	25.60	23.95	23.45	20.36	25.92	33.66	22.10
S1909	LPGPPPAsPIPTEGP	PCNX3	Q9H6A9	1.22	2.76	1.83	4.00	2.05	3.20	5.15	2.36	5.62	3.10	4.72	5.96
S1097	SALCNADsPKDPVLP	PDS5A	Q29RF7	6.82	7.56	7.81	9.84	7.29	8.78	7.95	7.76	9.40	12.08	11.15	11.48
T657	VDRKRRDtSGLERSH	PNN	Q9H307	4.38	4.39	4.15	2.97	6.26	6.03	7.58	7.59	9.76	7.75	4.40	8.88
T707	VPVSSNDtPPSALQE	POGZ	Q7Z3K3	1.22	0.97	0.92	2.15	2.87	0.87	0.89	0.80	2.58	1.96	2.25	2.49
T8	MGRKRLItDSYPVVK	POLD4	Q9HCU8	1.16	2.55	0.87	0.92	1.85	3.29	5.24	2.95	2.49	4.34	2.14	4.22
S527	IPLDEECsMDETPYV	PPP1R10	Q96QC0	1.29	1.02	1.93	3.07	1.03	2.10	1.87	0.84	2.95	2.48	3.54	2.82
S545	EPGGSGGsPDGAGGS	PPP1R10	Q96QC0	5.28	5.62	4.24	7.07	10.88	5.30	4.68	7.09	5.34	8.16	6.75	11.59
S78	RARSLPSsPERRQKA	PPP1R3D	O95685	47.49	44.86	50.36	47.95	47.22	49.00	80.18	65.72	75.99	56.60	65.61	69.65
S2079	EWRVRAIsAANLHLR	PRPF8	Q6P2Q9	3.86	2.86	2.80	3.28	5.34	5.39	2.90	3.54	5.07	5.78	7.18	5.20
S277	TVGTPIAsVPGSTNT	PSMD1	Q99460	8.62	8.48	9.74	9.94	11.09	9.05	16.09	12.32	13.08	11.77	10.72	10.51
S98	EQLGGGGsGGGGYNN	PUM1	Q14671	2.96	1.18	6.17	5.23	6.88	5.76	7.20	7.68	7.37	4.75	6.86	7.26
S773	STRVRHFsQSEETGN	RALGAPA1	Q6GYQ0	14.03	7.82	8.73	11.37	14.27	19.38	14.13	16.37	15.57	11.46	12.28	14.62
S2628	KKPEDSPsDDDVLIV	RANBP2	P49792	5.02	2.55	3.96	6.76	3.80	4.21	4.96	4.47	4.88	6.30	4.61	7.69
S583	QIKQEPEsEEEEEEK	RBM25	P49756	72.85	66.83	89.24	128.79	62.72	128.99	78.31	134.74	87.51	152.13	79.98	132.27

T208	FNGKFIKtPPGVSAP	RBMS1	P29558	4.25	5.01	2.89	5.02	6.67	6.67	4.58	5.91	7.18	5.47	5.25	5.20
S350	EKAEVYGsENESERN	RIOK2	Q9BVS4	5.79	8.07	6.27	7.99	6.67	8.87	10.01	8.52	15.29	10.33	10.08	10.51
S501	SQPSQPSsPLPGSHG	RNF214	Q8ND24	3.22	3.58	4.63	6.25	8.42	5.03	4.58	4.30	5.80	6.51	2.89	6.28
S9	GHQQLYWsHPRKFGQ	RPS29	P62273	2.70	3.47	4.24	3.07	3.80	4.02	5.71	4.56	5.16	5.68	6.75	4.12
S452	PVSPVKFsPGDFWGR	RPS6KB1	P23443	37.84	37.91	32.51	42.01	42.81	45.16	55.20	55.01	56.37	47.51	44.71	50.26
S503	LPIRQPNsGPYKKQA	RPS6KB1	P23443	1.87	2.96	7.53	6.97	7.49	8.32	9.92	6.92	11.15	6.92	8.26	7.80
S528	QEKIEPGsLNEEPFM	RPS6KC1	Q96S38	5.53	7.46	3.86	6.25	5.34	5.76	9.92	8.02	8.66	5.47	9.11	8.45
T77	GKSEAPEtPMEEEAE	RRP12	Q5JTH9	1.29	1.02	1.93	2.77	2.05	2.19	2.62	0.84	3.22	3.10	1.07	2.60
S212	AEDGVRGsPPVPSGP	SENP3	Q9H4L4	7.92	10.32	10.42	15.06	8.88	13.99	12.35	12.91	12.34	19.11	10.51	15.71
T14	TQFINPEtPGYVGFA	SEPTIN2	Q15019	1.42	1.12	2.12	3.48	1.13	1.01	2.43	2.36	2.58	3.10	1.18	1.19
T473	SSTGRRHtLAEVSTR	SIK1	P57059	5.66	2.96	2.80	9.12	5.34	7.59	5.43	6.67	7.55	5.99	6.43	7.15
T473	SSTGRRHtLAEVSTR	SIK1B	A0A0B4J2F2	5.66	2.96	2.80	9.12	5.34	7.59	5.43	6.67	7.55	5.99	6.43	7.15
S534	NFLEDNPsLKDIMLA	SIK2	Q9H0K1	2.77	2.20	3.04	4.66	3.85	3.98	3.88	4.43	4.10	4.39	4.93	3.52
T171	QPGIHPKtPNKFKKY	SLBP	Q14493	20.34	10.42	22.86	28.48	20.63	20.84	34.15	28.35	30.21	20.76	25.84	21.56
S417	KPLRRNNsYTSYTMA	SLC20A1	Q8WUM9	1.42	1.12	1.06	1.13	1.13	2.47	4.02	2.19	1.01	2.27	3.11	3.14
T478	GASQSDKtPEELFHP	SLC2A1	P11166	12.48	10.53	11.67	19.77	14.47	19.56	15.44	16.87	14.28	15.08	13.83	16.14
S297	EGHKLCLsTVDLEVK	SLC43A2	Q8N370	4.38	4.19	4.24	4.10	5.65	6.76	8.05	5.65	6.45	3.30	4.72	7.69
S709	TQTHENMsQLSEEEQ	SLC4A1AP	Q9BWU0	5.41	9.81	10.32	10.35	10.57	10.06	10.85	11.47	12.25	11.26	12.65	14.41
T479	PQLHFLDtDDEVSPT	SMG6	Q86US8	2.70	1.07	1.01	4.30	2.57	2.10	2.53	3.21	2.95	2.48	2.57	1.14
S484	LDTDDEVsPTSWGDS	SMG6	Q86US8	2.70	1.07	1.01	4.30	2.57	2.10	2.53	3.21	2.95	2.48	2.57	1.14
S1323	ELNQAWSsLGKRADQ	SPTAN1	Q13813	7.59	4.09	6.66	10.86	4.31	5.21	8.98	6.41	6.91	9.40	9.86	8.88
S17	DASQRRRsLEPAENV	SRC	P12931	1.61	3.17	2.60	2.56	3.59	3.57	4.02	4.64	3.32	1.29	5.36	3.36
S837	PSRQSHSsSSPHPKV	SRRM2	Q9UQ35	4.25	1.69	6.85	9.02	8.42	6.58	4.02	6.24	9.76	8.99	7.40	10.18
T714	KTKFICVtPTTCSNT	STAT3	P40763	1.29	2.04	2.99	1.02	5.95	3.38	3.37	3.88	3.32	3.82	3.43	3.79

S281	NRRQLAFsTVGTPDY	STK38	Q15208	8.11	4.39	4.53	11.47	7.80	7.50	9.92	6.50	7.18	11.57	9.11	6.93
S823	AAQAKQRsPSKVKPP	TCOF1	Q13428	9.27	4.60	7.91	13.01	11.09	13.99	13.19	13.08	14.09	7.64	13.51	9.86
S153	FPRGELEsPKPLVTP	TGIF2	Q9GZN2	8.11	8.58	11.29	10.45	10.47	14.90	13.10	15.19	13.91	13.84	10.83	12.78
S307	LFKPPEDsQDDESDS	THOC5	Q13769	1.48	3.17	2.41	4.41	4.00	5.30	1.08	3.97	3.96	5.16	3.65	2.49
S403	KYLQDQLsPLTRQRE	TNIP1	Q15025	1.48	1.18	2.51	1.18	2.57	2.38	2.25	0.97	4.70	2.38	4.18	3.03
S1585	NSSTSPAsPPGSIGD	TNRC6A	Q8NDV7	1.67	4.19	2.51	4.61	5.44	3.75	3.46	4.64	3.87	2.89	4.29	4.22
S154	QKRKRTEsSCVKSGS	TRIP12	Q14669	23.94	16.35	22.09	25.00	25.66	21.67	31.53	26.49	38.60	29.54	24.12	28.60
S8	MSSDRQRsDDESPST	UBE2E3	Q969T4	1.80	1.43	2.80	4.51	3.18	3.11	4.40	3.71	4.33	3.20	3.00	4.66
S130	LEESVSMsPEERARY	UCHL3	P15374	3.22	2.96	3.76	4.92	3.70	5.03	3.74	6.92	6.08	5.68	6.33	5.85
S42	RADGGGHsPTEVAGT	VAX2	Q9UIW0	1.42	2.66	1.06	1.13	2.57	2.38	2.34	1.86	2.21	3.10	3.22	2.82
S779	DMEEEKEsEDSDEEN	WDR75	Q8IWA0	13.06	13.69	21.13	16.50	20.12	16.91	19.18	14.85	22.20	24.89	21.55	29.14
S782	EEKESEDsDEENDFT	WDR75	Q8IWA0	13.06	13.69	21.13	16.50	20.12	16.91	19.18	14.85	22.20	24.89	21.55	29.14
S1978	KKEGPVAsPPFMDLE	WNK1	Q9H4A3	1.93	1.53	3.09	3.07	3.18	3.29	5.05	2.95	5.62	4.65	3.97	5.85
S883	TDAGGGLsSDEEEGT	XPC	Q01831	7.59	11.75	9.55	13.01	19.81	14.54	15.06	10.55	11.42	14.87	10.29	12.02
S884	DAGGGLSsDEEEGTS	XPC	Q01831	7.59	11.75	9.55	13.01	19.81	14.54	15.06	10.55	11.42	14.87	10.29	12.02
S164	AQHLRQSsFEIPDDV	YAP1	P46937	70.53	33.31	81.91	73.15	60.77	78.98	85.33	75.76	86.31	92.23	107.43	121.33
S61	IVHVRGDsETDLEAL	YAP1	P46937	10.43	7.87	13.80	12.70	13.04	16.18	17.78	12.06	19.62	13.32	16.40	15.71
S189	RFIPRPPsVAPPPMV	YBX2	Q9Y2T7	3.86	4.70	4.82	5.94	4.93	5.58	7.02	7.51	8.20	7.02	7.08	6.39
S293	TYRPRYRsRGPPRPR	YBX3	P16989	1.67	2.66	4.44	5.23	2.87	6.03	6.17	5.32	9.76	5.89	8.58	3.47
S1053	SHRCDAGsPSKDSAA	ZBED4	O75132	2.70	1.07	1.01	4.82	2.16	6.03	4.68	2.87	2.67	4.03	4.18	4.98
T168	QYLEAFAtATASGVP	ZBTB7B	O15156	1.35	1.07	2.51	2.15	2.26	4.21	2.43	0.89	2.03	2.48	2.47	3.90
S2801	LSPRTLLsPSSIKVE	ZFHX3	Q15911	1.54	2.96	2.41	3.69	2.87	3.75	2.99	2.02	2.30	3.61	4.29	4.33
S426	AGAAAPPsPPFSFQL	ZFP36L2	P47974	3.41	2.71	2.56	6.30	2.72	2.42	5.47	4.68	3.91	4.18	5.84	4.22
S1348	NAENNVPsCHHSQPA	ZGRF1	Q86YA3	1.54	2.86	2.41	2.87	2.46	4.94	4.12	4.13	7.00	3.82	2.57	5.20

S1352	NVPSCHHsQPAKLVM	ZGRF1	Q86YA3	1.54	2.86	2.41	2.87	2.46	4.94	4.12	4.13	7.00	3.82	2.57	5.20
S551	ASEYSPAsLDAFGAF	ZKSCAN1	P17029	2.83	2.76	3.38	3.48	5.34	4.02	4.49	5.48	4.97	6.51	5.47	5.96
S301	LLTSEEDsGFSTSPK	ZNF148	Q9UQR1	5.41	4.09	5.50	7.07	5.44	7.95	7.30	9.11	6.91	8.26	4.72	5.20
S412	LEQNQTIsPLSTYEE	ZNF148	Q9UQR1	17.12	13.90	25.95	22.44	22.48	20.75	29.66	23.88	32.79	27.47	29.80	23.18
S452	PSTPERQsSPSGSEQ	ZNF185	O15231	3.35	1.33	3.38	4.30	5.34	5.12	7.20	3.97	6.26	6.61	5.79	3.79
**5 µM ENDX:**															
S166	PKEPAPPsPESHSPR	ACBD4	Q8NC06	8.75	5.82	5.69	3.38	5.65	4.02	5.05	5.23	5.62	3.10	5.25	9.21
S332	PKAGLRVsAPEVSVG	AHNAK	Q09666	15.32	13.18	12.06	8.91	8.93	4.94	17.78	12.74	12.34	15.18	11.79	11.92
S5237	KIKFPKFsMPKIGIP	AHNAK	Q09666	57.66	41.08	36.18	28.79	28.54	29.62	57.63	46.07	46.89	61.14	44.28	61.31
T444	RYFDDEFtAQSITIT	AKT2	P31751	3.99	2.66	3.28	1.95	0.98	0.87	2.81	2.87	2.12	4.85	2.14	3.90
T1023	RRMLRRHtVEDAVVS	ANKRD27	Q96NW4	49.81	26.77	35.70	22.64	29.57	15.63	23.95	12.91	16.86	23.24	44.60	23.18
S675	AQLGAPTsPIPDSAI	ANKZF1	Q9H8Y5	3.86	3.17	4.53	1.95	2.67	2.93	1.96	5.48	5.71	3.30	4.93	2.60
S782	EKLCRANsRDGEAGR	ARHGEF2	Q92974	147.12	109.44	82.39	77.35	59.64	54.67	114.42	102.93	89.90	108.44	82.66	103.78
S557	VGESNLPsPSPTVSV	ASH1L	Q9NR48	4.12	5.11	5.40	3.18	1.75	3.20	3.37	3.29	3.87	5.58	2.36	3.79
S381	GKLFWKKsVKEDSVP	BCAS1	O75363	23.17	11.55	19.39	11.58	11.09	9.14	17.50	17.55	17.78	14.46	12.22	16.14
S332	STPSRTSsGLFPRIP	C17orf53	Q8N3J3	13.13	12.16	12.73	8.30	7.08	8.14	11.70	9.87	11.70	14.05	8.26	12.57
S878	PPPKRLSsVSGPSPE	CASKIN2	Q8WXE0	15.32	15.33	16.98	12.09	9.14	10.33	13.19	12.32	13.54	12.60	13.51	14.84
S250	KSKLDWEsFKEEEGI	CFDP1	Q9UEE9	16.99	13.80	13.22	10.96	8.01	6.95	13.57	14.26	19.62	16.01	17.80	14.62
S512	RSPRRGPsPACSDSS	CROCC	Q5TZA2	5.41	5.62	7.33	4.20	3.29	3.02	3.74	7.17	4.61	6.40	3.54	4.77
T85	STSSSSStPPLQPRD	CUL4B	Q13620	8.88	10.01	6.66	6.15	5.75	4.21	7.20	7.85	7.65	7.85	8.15	7.04
T172	CPHIVVGtPGRILAL	DDX39B	Q13838	8.88	10.73	7.72	6.56	6.57	4.94	6.55	4.13	6.54	7.95	5.25	9.53
S14	PNIPKAKsLDGVTND	DHCR7	Q9UBM7	180.45	176.07	122.91	114.65	101.01	92.88	162.33	143.34	132.92	170.92	146.02	171.59
S184	ESHTEAIsDAETSSS	DNTTIP2	Q5QJE6	3.99	7.15	4.82	2.66	3.49	3.20	2.81	6.50	3.87	1.34	5.79	4.33
S896	LNLNRSRsLSNSNPD	DOCK7	Q96N67	12.48	16.66	11.77	9.32	5.24	8.04	8.98	11.81	8.84	10.43	8.47	15.38

S470	YSNRKYEsDEDSLGS	EEF2K	O00418	5.28	3.78	5.50	2.66	2.57	2.10	3.74	2.19	4.97	3.51	4.61	3.90
S1257	GVRLRRPsVNGEPGS	EHBP1L1	Q8N3D4	28.32	33.11	30.97	19.77	19.50	22.21	19.93	24.72	22.20	26.23	17.90	26.11
S597	ASKYAALsVDGEDEN	EIF4B	P23588	25.48	15.33	17.08	12.09	12.63	9.23	13.94	20.75	14.74	18.80	15.76	16.03
S348	LGSRFRYsGKTEYQT	EPB41L5	Q9HCM4	92.29	78.38	56.05	53.89	45.48	43.24	68.21	56.36	57.20	62.17	57.57	58.82
S435	AGEVPARsPGAFDMS	EPN1	Q9Y6I3	4.50	4.70	3.86	3.79	2.52	2.24	3.37	4.05	5.43	5.06	2.63	4.55
S358	SGTVLSRsQPWDLTP	EPN3	Q9H201	4.89	4.39	2.51	2.46	2.26	2.38	2.25	5.40	2.30	5.68	3.86	4.01
S177	DKILPPPsPWPKSSI	GGA2	Q9UJY4	2.45	3.58	2.51	0.97	0.98	0.87	2.81	3.71	4.33	2.38	3.43	4.33
S3016	ASPETSAsPDGSQNL	GOLGB1	Q14789	3.73	4.70	5.40	3.18	2.57	1.74	3.09	3.21	3.13	3.82	3.00	3.47
S1160	PQGEHARsPQPRGPA	GREB1	Q4ZG55	7.59	11.85	11.09	6.04	7.29	4.85	4.58	6.24	9.58	13.63	11.79	8.77
S600	KLVSSAVsPSIIPQE	INTS4	Q96HW7	5.79	7.05	4.92	3.28	4.72	3.38	4.68	4.47	4.33	2.79	4.07	6.17
S180	SNMRMQFsFEGPEKV	IQSEC1	Q6DN90	19.18	19.72	23.25	14.14	12.22	14.72	23.67	16.11	20.45	18.28	19.51	24.59
S253	QVKSLAEsIDDALNC	IQSEC1	Q6DN90	4.50	4.50	3.76	2.97	2.57	2.83	3.74	4.22	4.88	4.54	3.75	4.77
S832	SSANLRHsPRVLVQH	KDM2A	Q9Y2K7	1.35	5.93	2.80	1.08	1.08	0.96	0.98	1.77	0.97	1.08	3.54	3.14
S897	ALASLPSsPGLLQSL	KDM5C	P41229	7.98	4.80	6.66	3.48	2.77	4.57	5.43	4.64	4.70	5.37	6.22	4.98
S12	VEKLTHLsYKEVPTA	LRATD2	Q96KN1	28.96	28.51	22.58	18.65	17.14	12.34	25.54	24.64	21.00	30.47	22.09	25.13
S148	AVPCTTRsPREGEVP	MAGI1	Q96QZ7	5.02	4.50	5.11	2.87	2.26	2.83	4.30	3.37	3.50	4.34	2.89	3.57
S775	SKSRRSAsPPTSLPS	MAP3K21	Q5TCX8	4.76	5.21	3.09	3.38	1.13	2.01	5.05	5.15	6.26	4.75	4.29	6.39
S639	PVLSRRDsPLQGSGQ	MAP4K4	O95819	10.17	6.95	9.55	7.27	5.24	4.57	8.79	8.10	7.74	7.13	9.97	8.23
S156	ACDGDKEsEVEDVET	MIER3	Q7Z3K6	14.93	12.88	10.71	9.84	6.98	8.59	8.61	13.25	11.05	13.53	11.15	9.64
S1030	FLLGSPGs-	MMS19	Q96T76	3.48	2.76	2.51	1.28	2.57	1.14	4.49	2.95	2.49	1.29	4.29	2.92
S107	TDMQRKRsSECLDGT	MON1A	Q86VX9	12.48	13.18	9.94	8.50	6.36	7.50	8.23	8.18	11.61	7.75	8.68	9.86
S145	KNAKKEDsDEEEDDD	NCL	P19338	3.73	3.27	2.70	2.36	2.26	1.01	1.03	3.21	3.22	4.34	2.68	2.82
S153	DEEEDDDsEEDEEDD	NCL	P19338	3.73	3.27	2.70	2.36	2.26	1.01	1.03	3.21	3.22	4.34	2.68	2.82
S1329	VITSQSDsPTRATDV	NHS	Q6T4R5	2.83	4.19	4.63	1.13	1.13	1.01	3.18	5.23	2.58	4.44	3.86	1.19

S411	PGHKRTPsEAERWLE	NUMBL	Q9Y6R0	10.81	10.73	9.94	6.56	7.08	6.12	6.36	8.10	10.50	9.40	11.04	8.99
S648	VTPKRSHsPSIDGTP	PAPOLG	Q9BWT3	11.20	10.93	12.83	5.12	9.55	8.32	8.51	8.35	12.07	10.84	10.51	14.41
S116	KDIIRQPsEEEIIKL	PEA15	Q15121	605.45	447.79	424.40	356.23	300.37	296.47	514.02	465.72	476.68	478.17	463.90	533.51
S537	PSLEPQKsLGDEGLN	PLCB3	Q01970	25.74	24.93	15.63	14.04	13.45	14.54	21.71	22.95	19.44	26.13	23.48	23.07
S77	FGESSTEsDEEEEEG	PPP1R11	O60927	3.86	6.85	5.98	2.46	4.62	5.21	3.18	3.97	3.41	5.47	3.86	6.17
S710	GEKSFRRsVVGTPAY	PRKD2	Q9BZL6	24.45	17.88	13.41	13.93	8.42	10.15	19.37	16.28	17.23	20.04	15.22	22.64
S973	GKPGESRsASSDTIE	PRRT3	Q5FWE3	6.56	8.28	8.39	4.51	4.93	4.30	5.43	4.56	6.26	7.13	6.75	7.04
T978	SRSASSDtIE-	PRRT3	Q5FWE3	6.56	8.28	8.39	4.51	4.93	4.30	5.43	4.56	6.26	7.13	6.75	7.04
S449	PLQEGPKsFDGNTLL	PTPN12	Q05209	53.03	42.61	40.62	22.23	19.71	21.57	36.30	44.72	38.23	53.08	38.70	50.81
S564	RSPIKRRsGLFPRLH	RAP1GAP2	Q684P5	13.13	12.16	12.73	8.30	7.08	8.14	11.70	9.87	11.70	14.05	8.26	12.57
S971	STVEEPVsPMLPPSA	REST	Q13127	2.83	4.60	2.60	1.08	2.77	0.96	3.46	1.77	3.22	3.72	2.25	4.33
S608	KTEELIEsPKLESSE	SCAF11	Q99590	13.51	9.30	14.37	7.79	10.68	6.22	12.63	8.86	9.76	10.43	10.61	10.40
S330	FVLHKSKsEEAHAED	SERBP1	Q8NC51	28.44	36.07	20.55	16.39	14.37	14.08	26.48	21.35	22.38	28.19	24.55	23.72
S21	NMKKQSDsVKGKRRD	SERF2	P84101	80.96	61.21	55.38	40.98	37.98	36.75	51.36	61.76	59.69	84.48	62.40	74.20
S1021	EQKKRSYsEPEKMNE	SHROOM3	Q8TF72	8.24	7.36	8.78	5.63	4.31	5.49	6.46	5.74	5.80	6.30	7.29	7.26
S1441	AHAAREDsLPEESSA	SHROOM3	Q8TF72	35.27	38.01	27.50	25.10	22.38	18.01	30.78	31.39	27.17	28.09	28.52	36.29
T148	LPPPLIPtPPPDDPR	SNX15	Q9NRS6	2.57	1.02	2.41	1.02	1.03	0.91	0.94	2.19	0.92	2.17	4.40	2.27
S136	SSNNGSAsPTKTKSG	SP4	Q02446	5.02	7.36	7.91	4.20	4.52	4.30	7.30	6.24	5.71	7.75	5.79	4.33
S13	FRRILRLsTFEKRKS	STK10	O94804	34.75	29.94	22.38	21.00	17.45	14.17	36.77	29.87	33.07	34.70	33.13	34.88
S707	LFREMEKsFDEQNVP	SVIL	O95425	63.33	58.55	48.14	42.31	35.42	34.74	67.55	51.89	50.75	65.99	56.93	68.03
S509	SAARKMPsKSLEDIS	SYTL2	Q9HCH5	47.62	32.70	26.92	18.34	16.43	14.90	36.11	30.71	33.62	44.51	33.66	31.96
S222	KTLVKSLsTDTSRQE	TEX2	Q8IWB9	78.90	71.43	51.61	53.69	35.62	37.12	64.84	54.67	57.29	67.03	62.08	84.93
S263	DPSSPRAsPAHSPRE	TLE1	Q04724	9.65	10.93	10.13	6.56	4.93	6.49	8.33	7.93	9.21	7.02	10.51	10.29
S267	PRASPAHsPRENGID	TLE1	Q04724	7.08	9.91	8.01	5.53	3.90	5.58	6.17	5.99	8.29	4.96	6.65	9.21

S24	VKYSRLSsTDDGYID	TMEM230	Q96A57	23.81	22.89	17.17	14.86	14.99	11.52	26.01	22.70	19.80	24.68	21.98	23.07
S46	RHGVLRHsVDLIGRP	TRMT61A	Q96FX7	5.28	5.11	5.02	2.56	3.90	2.93	5.05	3.80	5.99	4.44	5.47	4.66
S29	LPKDYLLsESEDEGD	UTP14A	Q9BVJ6	4.12	4.91	6.27	4.00	2.57	2.56	5.33	4.39	4.42	4.75	5.90	3.79
S31	KDYLLSEsEDEGDND	UTP14A	Q9BVJ6	4.12	4.91	6.27	4.00	2.57	2.56	5.33	4.39	4.42	4.75	5.90	3.79
S158	PIVSKSLsSSLDDTE	VAPB	O95292	9.91	8.99	10.23	3.48	4.62	7.13	6.36	8.27	9.12	4.23	7.18	7.91
S284	QALPGDLsGLFPRLR	VASN	Q6EMK4	13.13	12.16	12.73	8.30	7.08	8.14	11.70	9.87	11.70	14.05	8.26	12.57
S480	RGQAEEEsPSQEETV	ZBTB37	Q5TC79	5.15	6.34	5.02	2.87	3.80	3.66	4.40	3.54	3.13	4.44	3.22	6.50

Supplementary Table 3. List of the predicted upstream kinases and their corresponding substrates for a subset of phosphosites in cluster 1 as revealed by NetworKIN and RoKAI upstream kinase prediction tools.

NETWORKIN:							
#substrates	position	id (kinase)	networkin_score	netphorest_group	netphorest_score	string_score	sequence
CALR_HUMAN	80	PKCepsilon	4.065470903	PKC_group	0.137074	0.8	YALSAsFEPFS
SRRM2_HUMAN	1824	CLK1	2.875628747	CLK_group	0.362799	0.518	GYHSRsPARQE
DCP1A_HUMAN	315	MAPK1	2.19639563	MAPK3_MAP	0.310604	0.400591	YTIPLsPVLSP
HJURP_HUMAN	185	CDK1	6.421757393	CDK2_CDK3_	0.109025	0.784	LPSLAsPAVPA
CHMP5_HUMAN	86	PKCbeta	2.140616152	PKC_group	0.21809	0.17949	NLAQQsFNMEQ
LTBP1_HUMAN	1616	TGFbR2	2.299561278	ACTR2_ACTF	0.063905	0.599	DRFLNsFEELQ
IKZF1_HUMAN	258	PKCbeta	2.49482	PKC_group	0.337303	0.355444	LCKIGsERSLV
ACTB_HUMAN	202	CK1alpha	6.51385129	CK1_group	0.091339	0.577	GYSFTtTAERE
ACTB_HUMAN	199	CaMKIIalpha	2.633651739	CaMKII_group	0.136666	0.429647	TERGYsFTTTA
ACTB_HUMAN	199	PKCeta	4.6791866	PKC_group	0.105486	0.8	TERGYsFTTTA
ACTB_HUMAN	199	PKCtheta	4.6791866	PKC_group	0.105486	0.8	TERGYsFTTTA
ACTB_HUMAN	199	PKCdelta	2.463258217	PKC_group	0.105486	0.918	TERGYsFTTTA
ACTB_HUMAN	199	PKCalpha	2.416426046	PKC_group	0.105486	0.88	TERGYsFTTTA
ACTB_HUMAN	199	PKCgamma	4.6791866	PKC_group	0.105486	0.8	TERGYsFTTTA
ACTB_HUMAN	52	PKCeta	3.720939899	PKC_group	0.07651	0.8	MGQKDsYVGDE
ACTB_HUMAN	52	PKCtheta	3.720939899	PKC_group	0.07651	0.8	MGQKDsYVGDE
ACTB_HUMAN	52	PKCdelta	2.288892878	PKC_group	0.07651	0.918	MGQKDsYVGDE
ACTB_HUMAN	52	PKCgamma	3.720939899	PKC_group	0.07651	0.8	MGQKDsYVGDE
NDE1_HUMAN	282	CDK1	23.73356046	CDK2_CDK3_	0.265051	0.979	LVYDQsPNRTG
LEO1_HUMAN	140	CK2alpha	3.070586115	CK2_group	0.472848	0.382	SEKAHsDDEKW
H2BFS_HUMAN	56	PKCbeta	2.49482	PKC_group	0.27139	0.0948168	PDTGIsSKAMG
HMGB1_HUMAN	121	CK1alpha	3.716011139	CK1_group	0.080802	0.584	EHPGLsIGDVA
GTSE1_HUMAN	466	CDK1	7.897446404	CDK2_CDK3_	0.303688	0.485	SPDSStPKLSR
FLNA_HUMAN	962	MAP2K4	3.895767008	MAP2K_group	0.08979	0.918	PKSPFsVAVSP
FLNA_HUMAN	962	PAK1	7.594150906	PAK_group	0.138624	0.815	PKSPFsVAVSP
FLNA_HUMAN	962	PKCtheta	3.712592031	PKC_group	0.071264	0.914	PKSPFsVAVSP
HSPB1_HUMAN	98	DMPK1	5.820349691	DMPK_group	0.176467	0.864	DRWRVsLDVNH
SCRIB_HUMAN	1630	GRK2	3.629764457	GRK_group	0.116805	0.447223	LGPVPs-----
ALDOA_HUMAN	132	PKCbeta	2.401351502	PKC_group	0.233593	0.277559	GLDGLsERCAQ
TRAP1_HUMAN	393	TGFbR2	2.770734565	ACTR2_ACTF	0.064374	0.741	RGVVDsEDIPL
KRT84_HUMAN	178	PKCalpha	2.150968509	PKC_group	0.358835	0.173082	NNKFAsFIDKV
KRT84_HUMAN	178	PKCbeta	2.49482	PKC_group	0.358835	0.152893	NNKFAsFIDKV

ROKAI:										
KinID	KinName	KinGene	SubsProtein	SubsGene	Position	Flanking	Quantification	ZScore	PValue	FDR
P31749	Akt1	AKT1	P02545	LMNA	S301	QsRIRIDsLsAQLsQ	-0.47	-1.732	0.0833	0.0835
P31749	Akt1	AKT1	P06899	H2BC11	S37	RKRSRkEsysIyVyk	-1.79	-6.597	4.21e-11	1.37e-9
Q6P2M8	CAMK1B	PNCK	P07900	HSP90AA1	S391	FIrGVVDsEDLPLNI	-1.09	-4.017	0.000059	0.000347
O15530	PDK1	PDPK1	P08559	PDHA1	S232	NRyGMGtsVERAAAs	-0.64	-2.359	0.0183	0.0204
Q15118	PDHK1	PDK1	P08559	PDHA1	S232	NRyGMGtsVERAAAs	-0.64	-2.359	0.0183	0.0204
Q15119	PDHK2	PDK2	P08559	PDHA1	S232	NRyGMGtsVERAAAs	-0.64	-2.359	0.0183	0.0204
Q16654	PDHK4	PDK4	P08559	PDHA1	S232	NRyGMGtsVERAAAs	-0.64	-2.359	0.0183	0.0204
P49841	GSK3B	GSK3B	P22670	RFX1	S120	SETVsEAsPGStAsQ	-0.71	-2.616	0.00888	0.0118
P17252	PKCA	PRKCA	P23528	CFL1	S23	NDMkVRksstPEEVk	-0.94	-3.464	0.000532	0.00113
O43318	TAK1	MAP3K7	P68104	EEF1A1	T261	ykIGGIGtVPVGrVE	-0.83	-3.059	0.00222	0.00355
P17612	PKACA	PRKACA	Q14289	PTK2B	S778	HNVFKRHsMREEDFI	-1.13	-4.164	0.0000312	0.000209
P31749	Akt1	AKT1	Q71DI3	H3C14	T46	PHryrPGtVALrEIR	-1.24	-4.57	0.00000489	0.0000495
P45983	JNK1 iso2	MAPK8	Q9NPI6	DCP1A	S315	PtytIPLsPVLsPtL	-1.04	-3.833	0.000127	0.000472
P06493	CDK1	CDK1	Q9NXR1	NDE1	S282	RNLVyDQsPNRtGGP	-0.65	-2.395	0.0166	0.0188

Supplementary Table 4. *In vitro* kinase assay showing the IC_{50} concentration of ENDX and staurosporine (a broad-spectrum kinase inhibitor) on the kinase activity (shown in percentage (%)) of the 12 PKC family members.

PKC family kinase	Symbol	ENDX IC50* (M)	Staurosporine IC50 (M)
PKC alpha	PKCa	>5.00E-05	<1.00E-09
PKC beta 2	PKCb2	4.33E-05	<1.00E-09
PKC delta	PKCd	n/a*	<1.00E-09
PKC epsilon	PKCe	n/a*	<1.00E-09
PKC eta	PKCh	>5.00E-05	<1.00E-09
PKC gamma	PKCg	4.97E-05	1.65E-09
PKC iota	PKCi	>5.00E-05	1.84E-08
PKC mu	PKCm/PRKD1	3.16E-05	1.24E-09
PKC nu	PKCn/PRKD3	2.64E-05	<1.00E-09
PKC thetha	PKCq	4.18E-05	1.54E-09
PKC zeta	PKCz	4.81E-05	7.72E-08

*: not available due to no inhibition or compound activity that could not be fit to an IC50 curve. Staurosporine serves as the positive control.

Supplementary Table 5. List of the primary antibodies used in this study. Information about the vendor, product catalog number and antibody dilution used in the IB assay are provided.

**Protein name**	**Primary Antibody Species**	**Vendor and Catalog #**	**Dilution**
phospho-AKT^{Ser473}	Rabbit	CST #9271	1:1000
phospho-AKT^{Thr308}	Rabbit	CST #9275	1:1000
AKT	Rabbit	CST #9272	1:1000
phospho-AKT substrate	Rabbit	CST #9614	1:1000
phospho-PKCβ1^{Ser661}	Rabbit	Abcam #192184	1:1000
ERα	Mouse	SantaCruz #8002	1:500
PKCβ1	Rabbit	Abcam #136917	1:1000
PKCβ2	Mouse	SantaCruz #sc-13149	1:200
cleaved PARP	Rabbit	CST #5625	1:500
PARP	Rabbit	CST #9542	1:1000
β-actin	Mouse	Sigma #A2228	1:20,000
HA-tag	Rat	Roche #11867423001	1:4,000

SUPPLEMENTARY FIGURES:

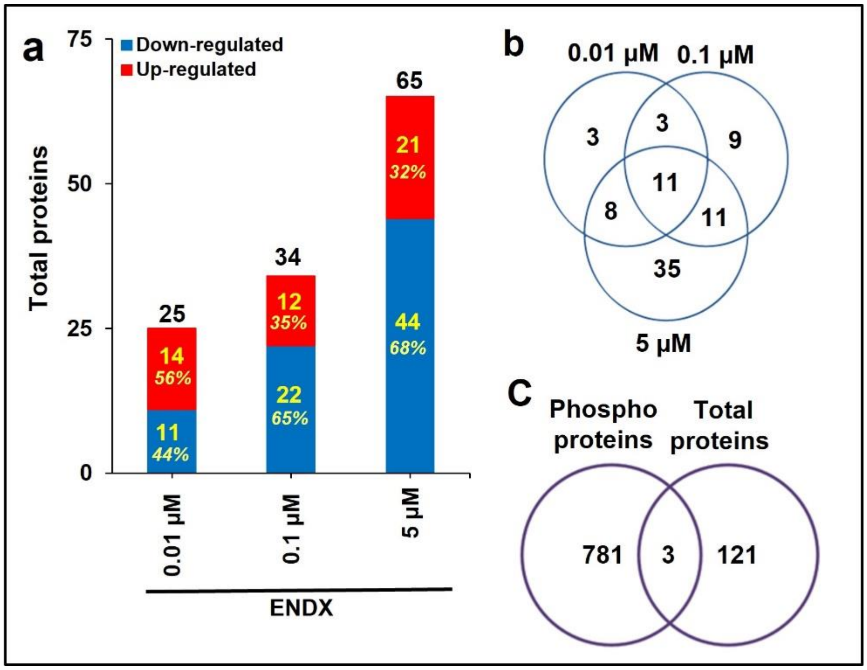

Supplementary Figure 1. Effects of ENDX on the global protein expression in MCF7AC1 cells. a, Stacked barplot showing the number of total proteins and the percentage that are upregulated (red) and downregulated (blue) (Fold change (FC) |1.5|; p value < 0.05) in 0.01, 0.1 and 5 μM ENDX treated cells relative to vehicle treated cells for 24 hours in CSS medium, as identified by mass spectrometry analysis. **b**, Venn diagram indicating the overlap of the total proteins in the 0.01, 0.1 and 5 μM ENDX treated cells relative to vehicle treated cells. **C**, Venn diagram indicating the overlap of the list of the phosphosites and the list of the total proteins that are altered by ENDX treatment regardless of the concentration.

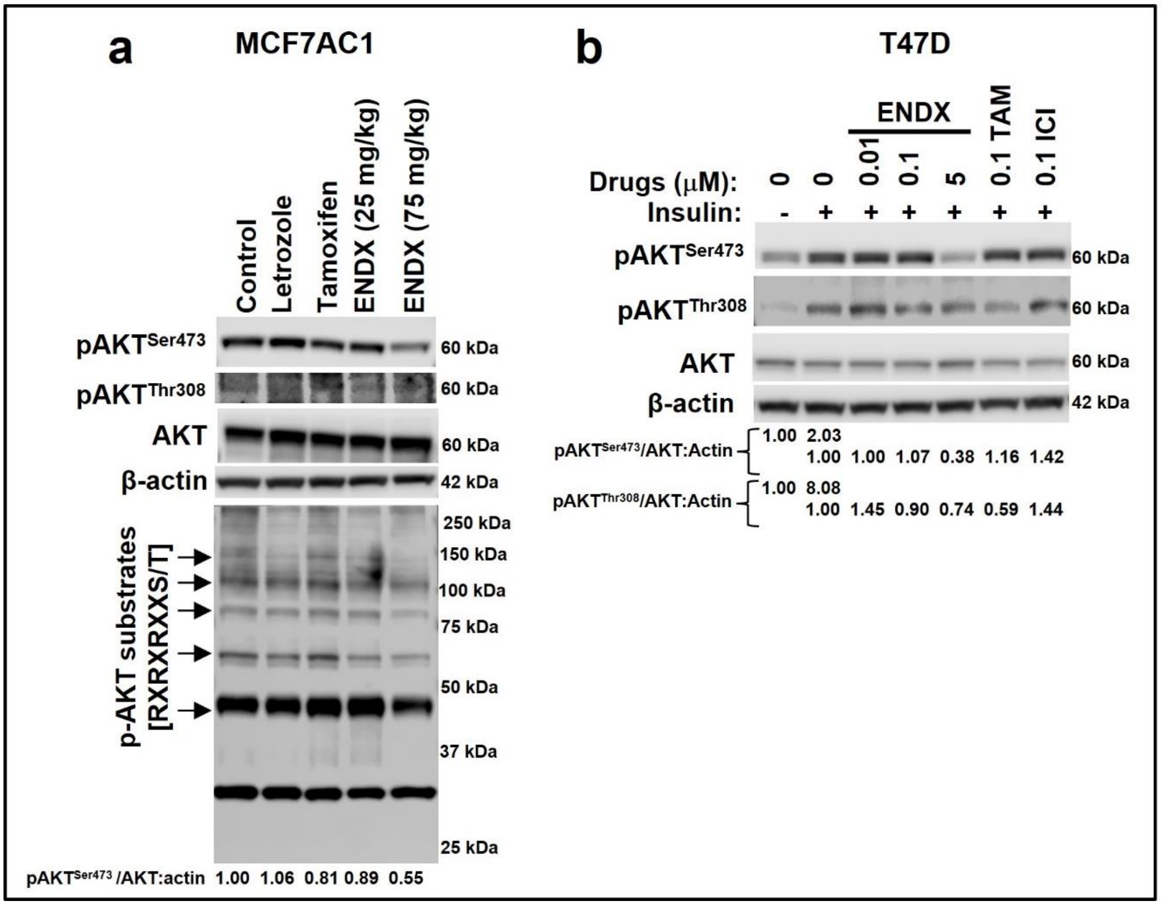

Supplementary Figure 2. ENDX effects on AKT phosphorylations in ERα+ breast cancer models *in vivo* and *in vitro*. **a**, MCF7AC1 xenograft tumors were treated with control, letrozole, tamoxifen (TAM) and 25 mg/kg and 75 mg/kg ENDX for four weeks *in vivo.* IB assay of $pAKT^{Ser473}$, $pAKT^{Thr308}$, AKT, p-AKT substrates are shown with β-actin as a loading control. **b**, Serum starved T47D cells were pretreated with vehicle control or 0.01, 0.1 and 5 µM ENDX and 0.1 µM tamoxifen (TAM) and ICI-182780 (ICI) for two hours followed by the addition of 100 nM insulin treatment for one hour. IB assay of $pAKT^{Ser473}$, $pAKT^{Thr308}$, AKT and β-actin.

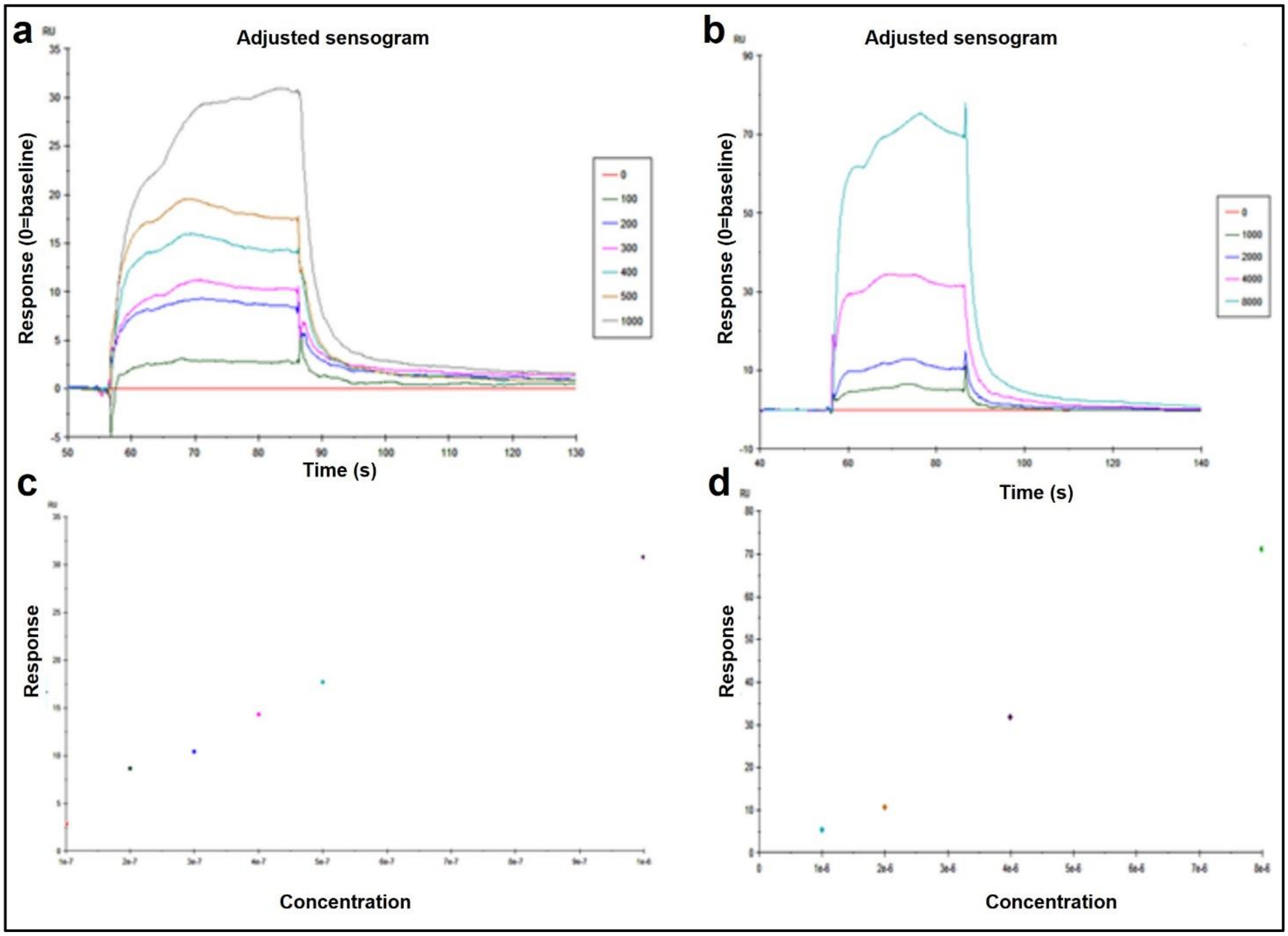

Supplementary Figure 3. ENDX binds to PKCβ1. a and **b**, SPR sensograms (relative units, RU) of ENDX binding at the indicated concentrations to immobilized PKCβ1**. c** and **d**, Dot plot showing the affinity binding of ENDX to PKCβ1 corresponding to sensograms **a** and **b**, respectively.

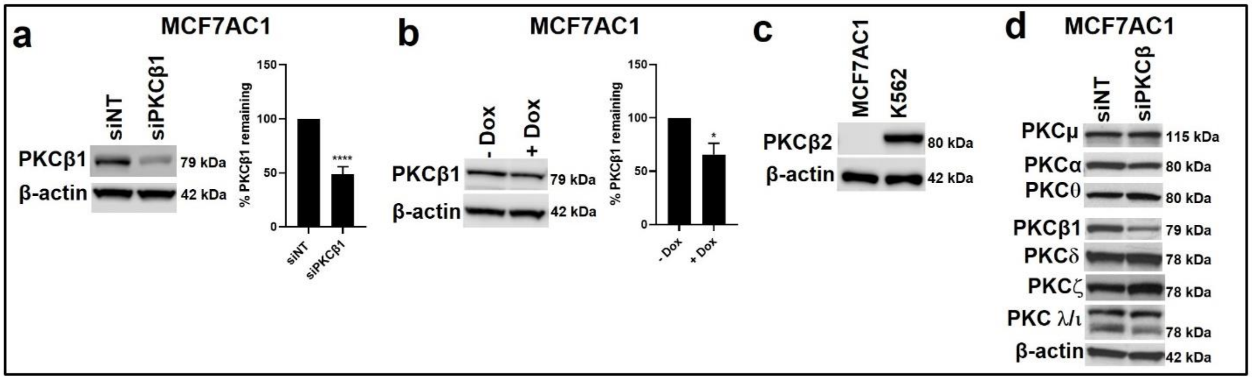

Supplementary Figure 4. PKCβ1 knockdown by different strategies and the effects of PKCβ1 knockdown on PKC family members. **a**, MCF7AC1 cells in CSS medium were transfected with non-targeting (siNT) or PKCβ1-targeting (siPKCβ1) siRNAs for 72 hours. IB assay of PKCβ1 and β-actin are shown. The histogram indicates the percentage (%) of PKCβ1 protein levels remaining in siPKCβ1-transfected cells compared to siNT-transfected cells. **b**, MCF7AC1 cells in CSS medium in the absence (-) or presence (+) of doxycycline (Dox) for 72 hours. IB assay of PKCβ1 and β-actin are shown. The histogram indicates the percentage (%) of PKCβ1 protein levels remaining in dox induced cells compared to noninduced cells. **c**, IB assay of basal PKCβ2 protein expression and β-actin in MCF7AC1 and K562 (a positive control for PKCβ2) cells. **d**, IB assay of the relative protein expression of PKC family members and β-actin in siNT and siPKCβ1 transfected MCF7AC1 cells. For (**a**) and (**b**) data represents mean of six wells per treatment performed as biological triplicates ± s.d. *, $p < 0.05$; ****, $p < 0.0001$ by one sample t test.

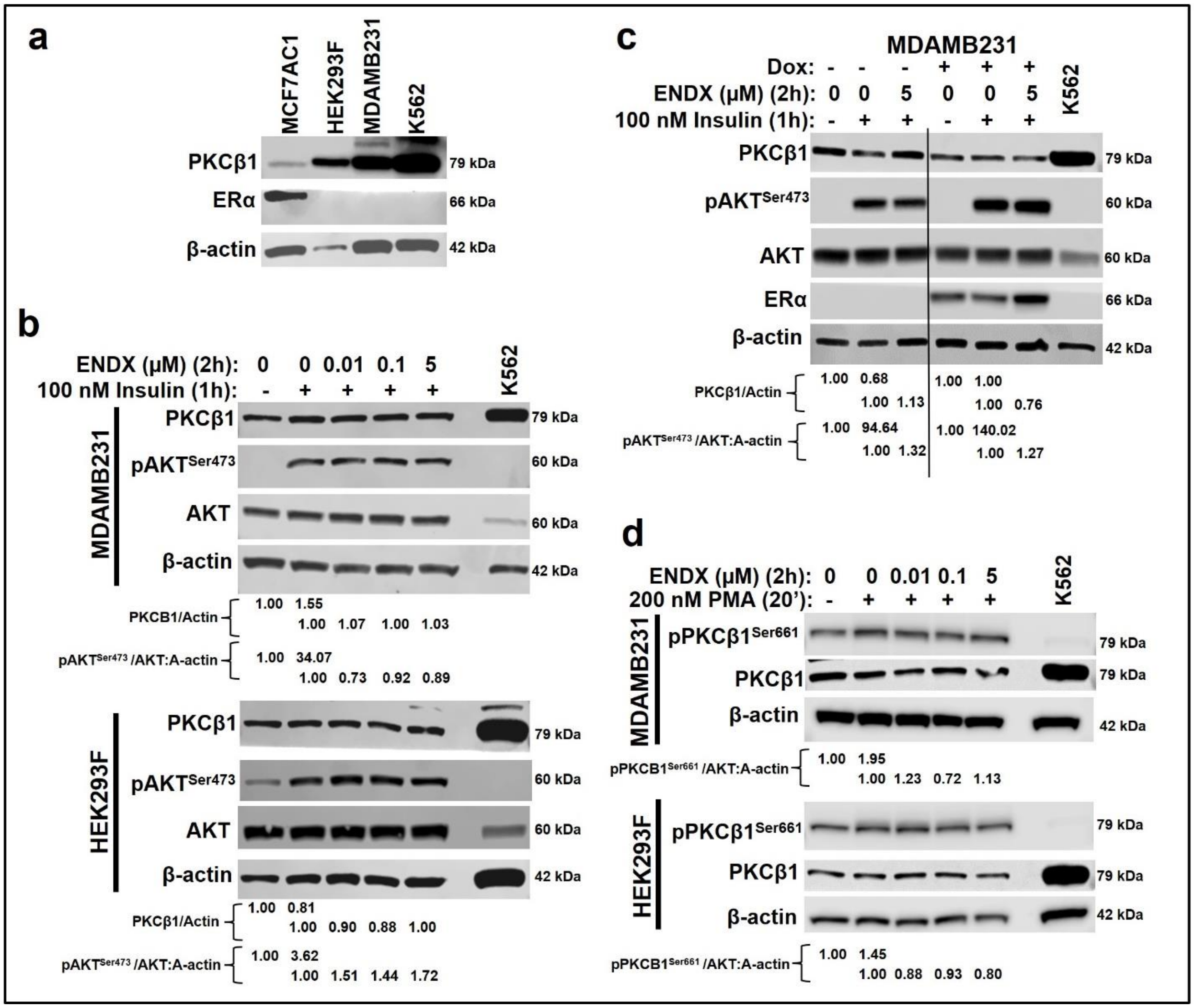

Supplementary Figure 5. Effects of ENDX on PKCβ1, phospho-PKCB1^{Ser661} and phospho-AKT^{Ser473} expression levels in ERα- breast cancer cells. a, Basal expression of PKCβ1 and ERα in the indicated cell lines. K562 cells serves as the positive control for PKCβ1. **b**, The effects of ENDX pretreatment for two hours followed by treatment in the presence or absence of 100 nM insulin for one hour on the protein expression levels of PKCβ1, pAKT^{Ser473}, AKT and β-actin in MDAMB231 and HEK293F cells. **c**, The effects of ENDX pretreatment for two hours followed by treatment in the presence or absence of 100 nM insulin for one hour on the protein expression levels of PKCβ1, pAKT^{Ser473}, AKT, ERα and β-actin in MDAMB231-ERα cells. Dox was added 48 hours prior to the treatments to allow for induction of ERα protein expression. **d**, The effects of ENDX pretreatment for two hours followed by treatment in the presence or absence of 20 nM PMA for 20 minutes on the protein expression levels of pPKCβ1^{Ser661}, PKCβ1 and β-actin in MDAMB231 and HEK293F cells.

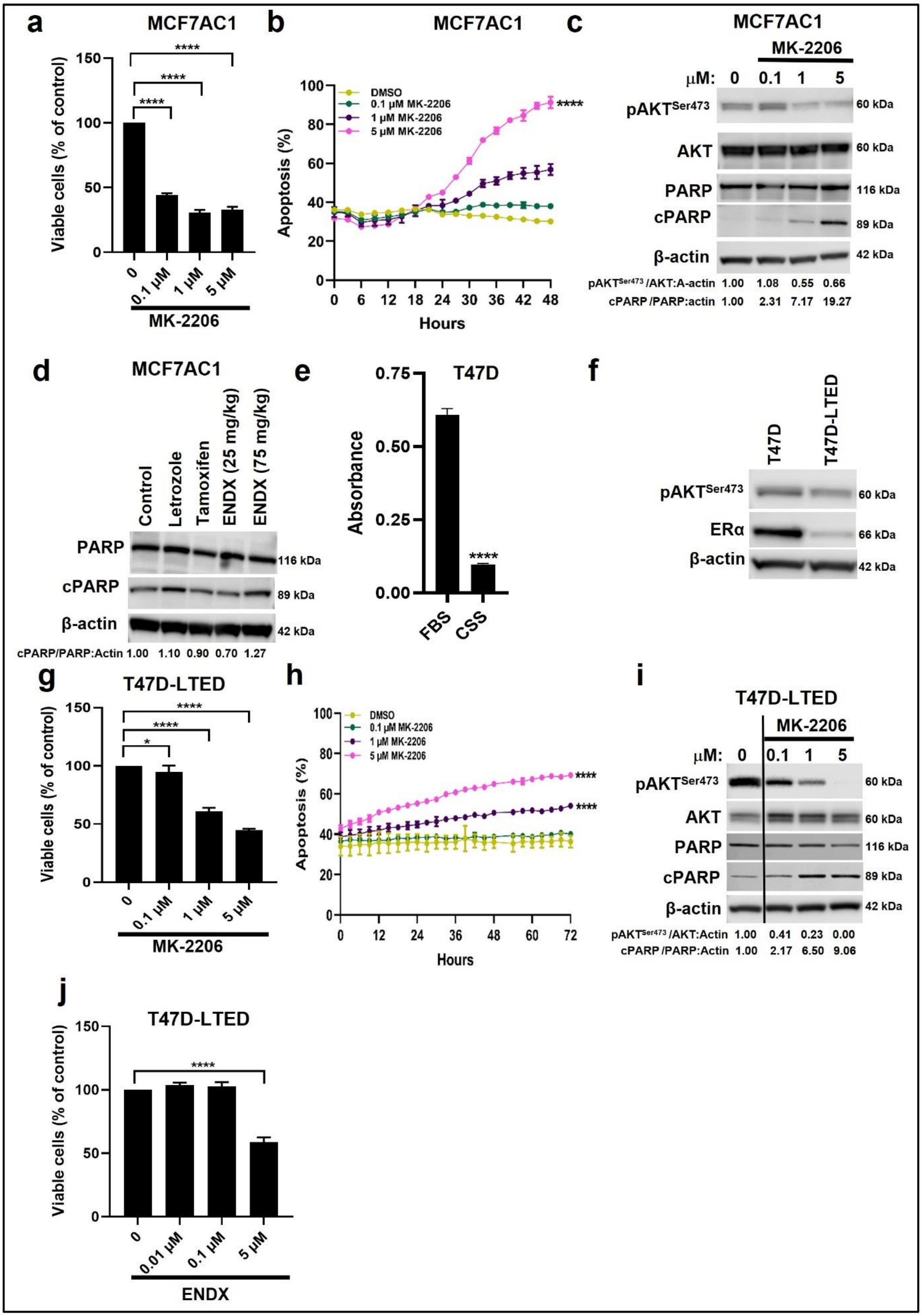
a
MCF7AC1
Viable cells (% of control)
MK-2206
b
MCF7AC1
Apoptosis (%)
Hours
DMSO
0.1 µM MK-2206
1 µM MK-2206
5 µM MK-2206
c
MCF7AC1
MK-2206
µM: 0 0.1 1 5
pAKTSer473
AKT
PARP
cPARP
β-actin
pAKTSer473 /AKT:A-actin 1.00 1.08 0.55 0.66
cPARP /PARP:actin 1.00 2.31 7.17 19.27
d
MCF7AC1
Control
Letrozole
Tamoxifen
ENDX (25 mg/kg)
ENDX (75 mg/kg)
PARP
cPARP
β-actin
cPARP/PARP:Actin 1.00 1.10 0.90 0.70 1.27
e
T47D
Absorbance
FBS
CSS
f
T47D
T47D-LTED
pAKTSer473
ERα
β-actin
g
T47D-LTED
Viable cells (% of control)
MK-2206
h
Apoptosis (%)
Hours
DMSO
0.1 µM MK-2206
1 µM MK-2206
5 µM MK-2206
i
T47D-LTED
MK-2206
µM: 0 0.1 1 5
pAKTSer473
AKT
PARP
cPARP
β-actin
pAKTSer473 /AKT:Actin 1.00 0.41 0.23 0.00
cPARP /PARP:Actin 1.00 2.17 6.50 9.06
j
T47D-LTED
Viable cells (% of control)
ENDX

Supplementary Figure 6. Effects of the pan-AKT inhibitor MK-2206 or ENDX on phenotypes in ERα+ breast cancer cells. a, MCF7AC1 cells were treated with vehicle control or 0.01, 0.1 and 5 µM MK-2206 in CSS medium for six days. Cell viability is assessed by the crystal violet assay. **b**, MCF7AC1 cells were co-treated with vehicle control or 0.01, 0.1 and 5 µM MK-2206 and IncuCyte Annexin V green and NucLight rapid red reagents in CSS medium for 48 hours. The apoptosis graphs are presented as the green object count divided by the red object count and displayed as percentage using the IncuCyte S3 analysis software. Cells were plated at a density of 2000 cells per well. **c**, MCF7AC1 cells were treated with vehicle control or 0.01, 0.1 and 5 µM MK-2206 in CSS medium for 24 hours. IB assay of $pAKT^{Ser473}$, AKT, PARP, cleaved PARP and β-actin**. d,** MCF7AC1 xenograft protein lysates were treated with the indicated drugs for four weeks. IB assay of cPARP, PARP and β-actin. **e**, T47D cells were cultured in FBS versus CSS medium for six days. Cell viability is assessed by the crystal violet assay. **f**, Parental T47D and T47D-LTED cells were processed for protein lysates. IB assay of ERα, $pAKT^{Ser473}$ and β-actin. **g**, T47D-LTED cells were treated with vehicle control or 0.01, 0.1 and 5 µM MK-2206 in CSS medium for six days. Cell viability is assessed by the crystal violet assay. **h**, T47D-LTED cells were co-treated with vehicle control or 0.01, 0.1 and 5 µM MK-2206 and IncuCyte Annexin V green and NucLight rapid red reagents in CSS medium for 48 hours. Percentage of cells undergoing apoptosis was calculated as mentioned in b. **i**, T47D-LTED cells were treated with vehicle control or 0.01, 0.1 and 5 µM MK-2206 in CSS medium for 24 hours. IB assay of $pAKT^{Ser473}$, AKT, PARP, cleaved PARP and β-actin. **i**, T47D-LTED cells were treated with vehicle control or 0.01, 0.1 and 5 µM ENDX in CSS media for six days. Cell viability is assessed by the crystal violet assay. For figs. (**a**), (**b**), (**e**), (**g**), (**h**) and (**j**) data represents mean of six wells per treatment performed as biological duplicates ± s.d. *, $p \leq 0.05$, ****, $p \leq 0.0001$ by one-way ANOVA for figs. (**a**), (**b**), (**g**), (**h**) and (**i**) and unpaired t test for fig. (**e**).

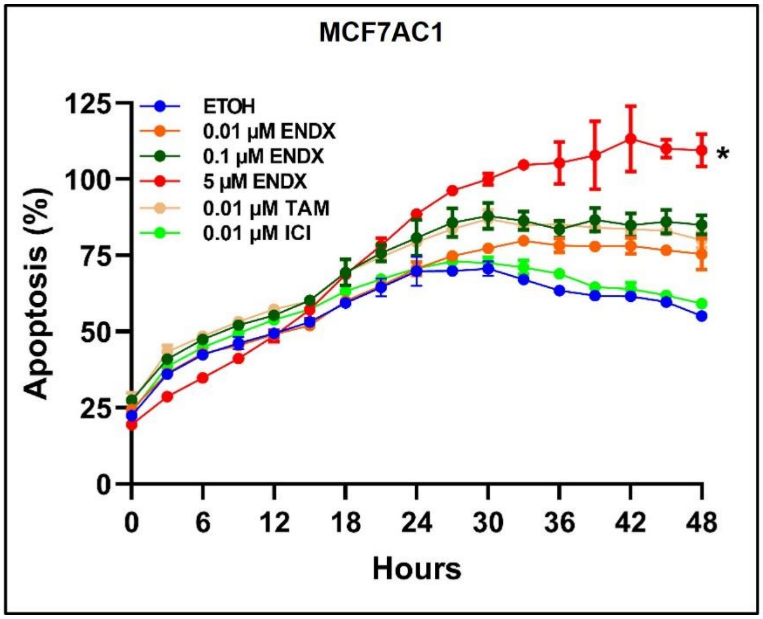

Supplementary Figure 7. Unlike ENDX, TAM and ICI do not induce apoptosis. MCF7AC1 cells were co-treated with vehicle control or 5 µM ENDX, 0.1 µM TAM and 0.1 µM ICI and IncuCyte Annexin V green and NucLight rapid red reagents in CSS medium for 48 hours. The percentage (%) of cells undergoing apoptosis is calculated as described in Supplementary Fig. S7. Cells were plated at a density of 2000 cells per well. Data represents mean of six wells per treatment performed as biological duplicates ± s.d. *, $p \leq 0.05$ by one-way ANOVA.

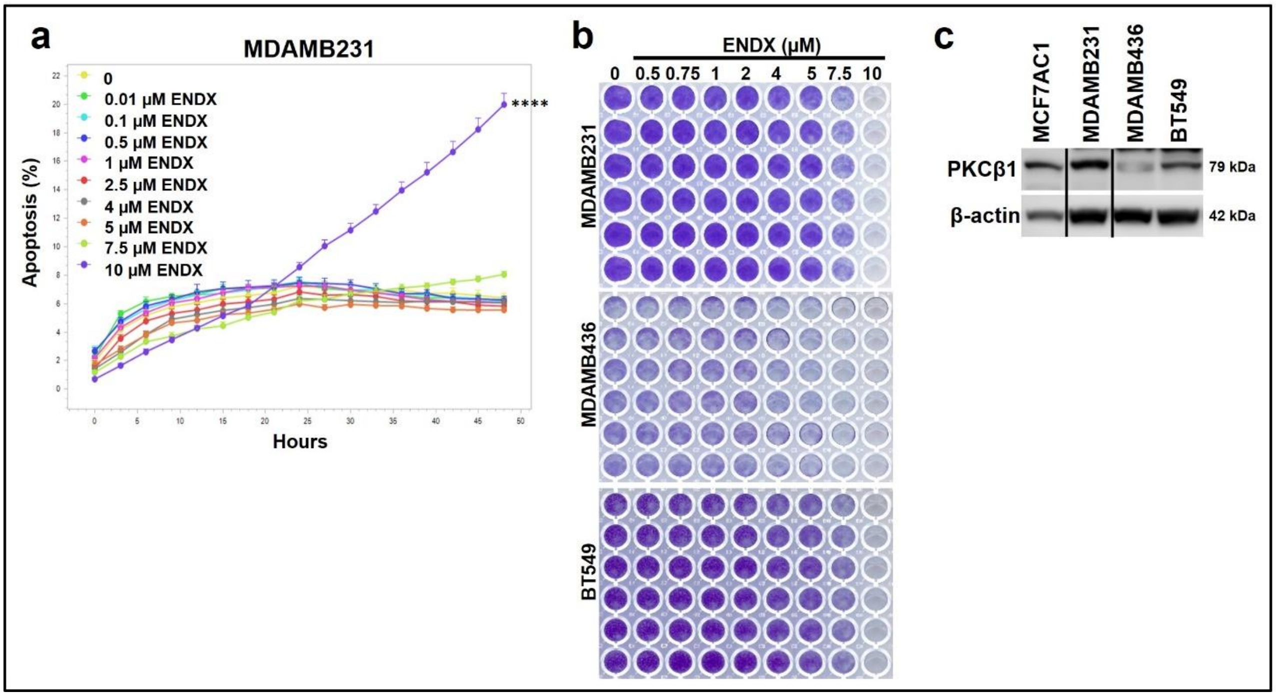

Supplementary Figure 8. ENDX neither induces apoptosis nor inhibit growth of ER- breast cancer cells. a, MDAMB231 cells grown in CSS medium were were co-treated with vehicle control or the indicated ENDX concentrations, IncuCyte Annexin V green and NucLight red reagents in CSS medium for 48 hours. The apoptosis graphs are presented as the green object count divided by the red object count and displayed as percentage using the IncuCyte S3 analysis software. Cells were plated at a density of 2000 cells per well. Data represents the mean of six wells per treatment performed as biological duplicates ± s.d. ****, $p \leq 0.0001$ by one-way ANOVA. **b**, Effects of ENDX on cell proliferation at the indicated concentrations on day seven of treatment. Cell viability was assessed by the crystal violet assay. **c**, immunoblot of basal PKCβ1 protein expression in the indicated cell lines.

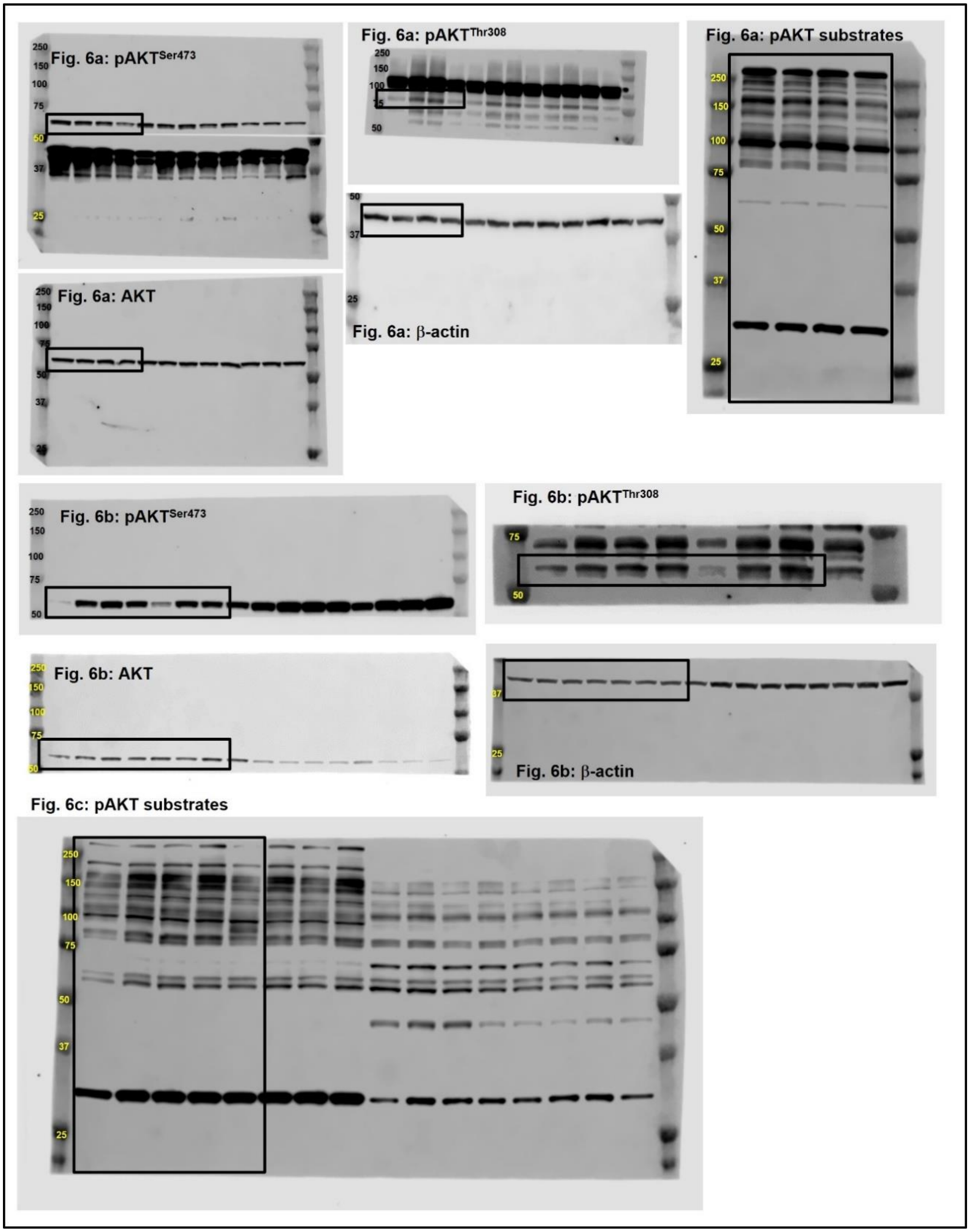

Supplementary Figure 9: The uncropped immunoblot images of $pAKT^{Ser473}$, $pAKT^{Thr308}$, AKT, pAKT substrates and β-actin (loading control) from Figures 6a-6c is highlighted within the box. The membranes were developed in Li-Cor image analysis software using SuperSignal West Pico PLUS Chemiluminescent substrate (WP) (ThermoFisher, Catalog #34580) or SuperSignal West Femo maximum sensitivity substrate (WF) (ThermoFisher, Catalog #34096). The numbers denote protein molecular weight in kDa.

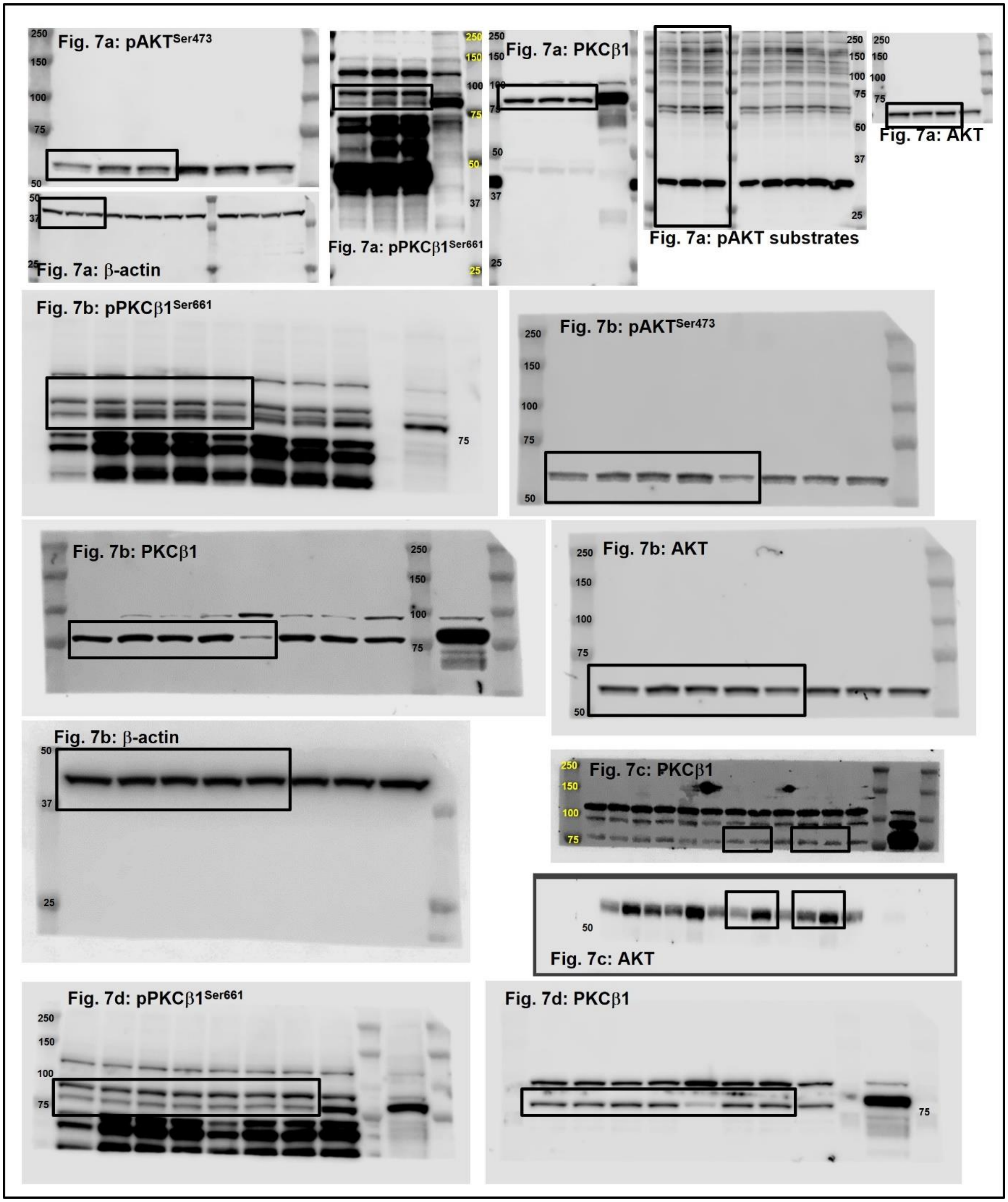

Supplementary Figure 10: The uncropped immunoblot images of pAKT^{Ser473}, AKT, pAKT substrates, pPKCβ1^{Ser661}, PKCβ1, and β-actin from Figures 7a-7d is highlighted within the box. The membranes were developed in Li-Cor image analysis software using WP or WF chemiluminescent substrates. The numbers denote protein molecular weight in kDa.

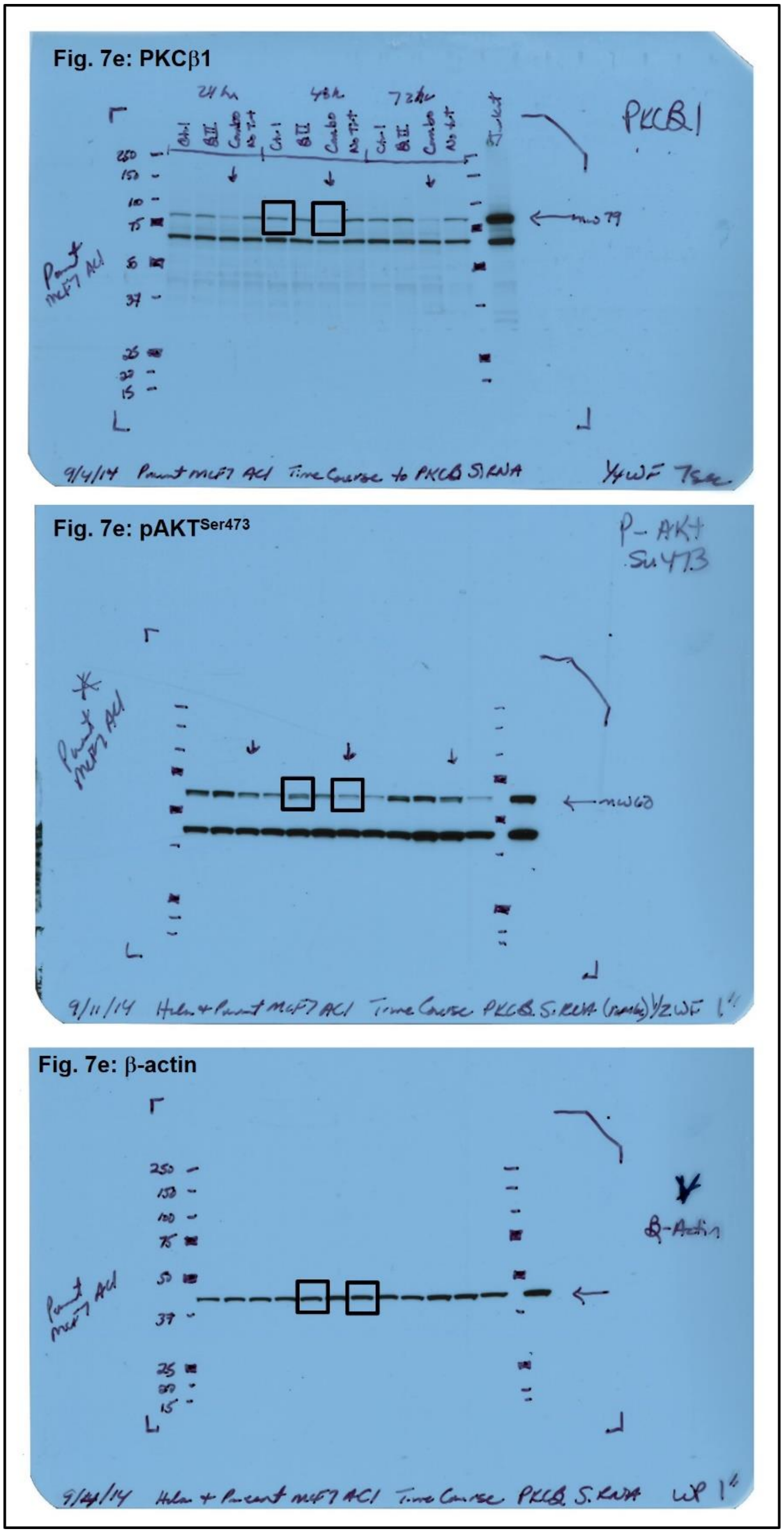

Supplementary Figure 11: The uncropped immunoblot images of pAKT^{Se4731}, PKCβ1, and β-actin shown from Figure 7e is highlighted within the box. The membranes were developed by X-ray films using WP or WF chemiluminescent substrates. The numbers on the end of the membrane and within parentheses denotes molecular weight in kDa.

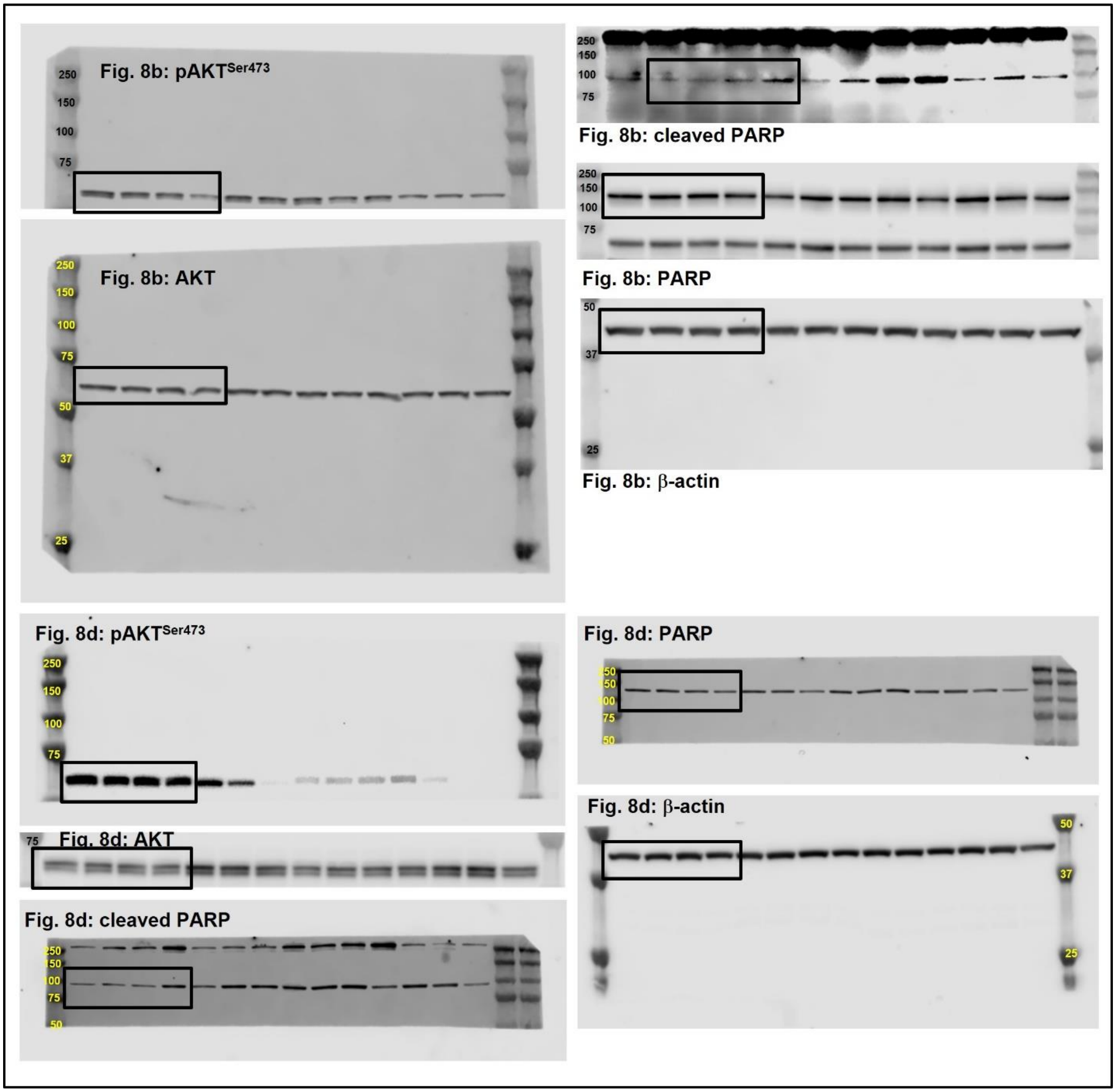

Supplementary Figure 12: The uncropped immunoblot images of pAKT^{Ser473}, AKT, cleaved PARP, PARP, and β-actin from Figures 8a-8d is highlighted within the box. The membranes were developed in Li-Cor image analysis software using WP or WF chemiluminescent substrates. The numbers denote protein molecular weight in kDa.

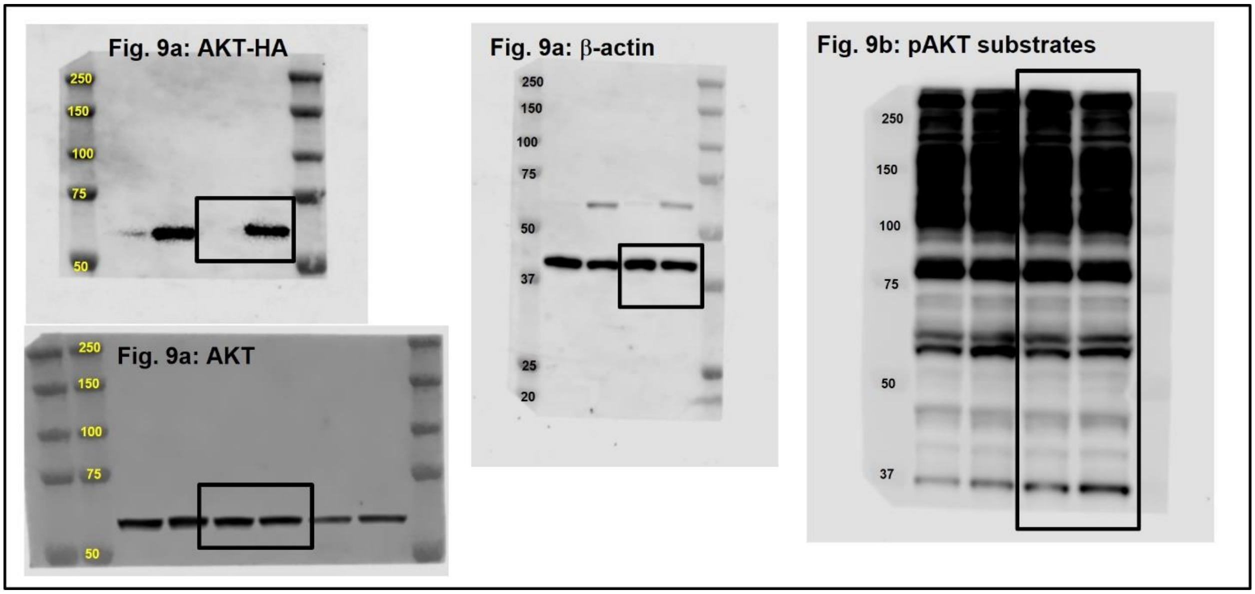

Supplementary Figure 13: The uncropped immunoblot images of AKT-HA, AKT, pAKT substrates and β-actin from Figures 9a and 9b is highlighted within the box. The membranes were developed in Li-Cor image analysis software using WP or WF chemiluminescent substrates. The numbers denote protein molecular weight in kDa.
