# Supplementary material for: Endoxifen downregulates AKT phosphorylation through protein kinase C beta 1 inhibition in ERα+ breast cancer
Source: NPJ Breast Cancer. 2023 Dec 19;9:101. doi: 10.1038/s41523-023-00606-2 (PMC10730845; doi:10.1038/s41523-023-00606-2)
Supplement: Supplementary file 2 — Reporting Summary [file 41523_2023_606_MOESM2_ESM.pdf]

nature portfolio

Corresponding author(s): Matthew P. Goetz

Last updated by author(s): Nov 20, 2023

Reporting Summary

Nature Portfolio wishes to improve the reproducibility of the work that we publish. This form provides structure for consistency and transparency in reporting. For further information on Nature Portfolio policies, see our Editorial Policies and the Editorial Policy Checklist.

Statistics

For all statistical analyses, confirm that the following items are present in the figure legend, table legend, main text, or Methods section.

n/a	Confirmed	
☐	☒	The exact sample size (n) for each experimental group/condition, given as a discrete number and unit of measurement
☒	☐	A statement on whether measurements were taken from distinct samples or whether the same sample was measured repeatedly
☐	☒	The statistical test(s) used AND whether they are one- or two-sided<br>*Only common tests should be described solely by name; describe more complex techniques in the Methods section.*
☒	☐	A description of all covariates tested
☒	☐	A description of any assumptions or corrections, such as tests of normality and adjustment for multiple comparisons
☒	☐	A full description of the statistical parameters including central tendency (e.g. means) or other basic estimates (e.g. regression coefficient) AND variation (e.g. standard deviation) or associated estimates of uncertainty (e.g. confidence intervals)
☒	☐	For null hypothesis testing, the test statistic (e.g. F, t, r) with confidence intervals, effect sizes, degrees of freedom and P value noted<br>*Give P values as exact values whenever suitable.*
☒	☐	For Bayesian analysis, information on the choice of priors and Markov chain Monte Carlo settings
☒	☐	For hierarchical and complex designs, identification of the appropriate level for tests and full reporting of outcomes
☒	☐	Estimates of effect sizes (e.g. Cohen's d, Pearson's r), indicating how they were calculated

Our web collection on statistics for biologists contains articles on many of the points above.

Software and code

Policy information about availability of computer code

Data collection	No software were used.
Data analysis	No data were used.

For manuscripts utilizing custom algorithms or software that are central to the research but not yet described in published literature, software must be made available to editors and reviewers. We strongly encourage code deposition in a community repository (e.g. GitHub). See the Nature Portfolio guidelines for submitting code & software for further information.

Data

Policy information about availability of data

All manuscripts must include a data availability statement. This statement should provide the following information, where applicable:

- Accession codes, unique identifiers, or web links for publicly available datasets
- A description of any restrictions on data availability
- For clinical datasets or third party data, please ensure that the statement adheres to our policy

All mass spectrometry datasets acquired for this study were deposited to ProteomeXchange (http://proteomecentral.proteomexchange.org) and are available via the accession number PXD035007. The reviewer can access the dataset with the Username: reviewer_pxd035007@ebi.ac.uk and password: 9kkfUJ4E. All other relevant data are included in the manuscript or available from the corresponding author upon request.

Research involving human participants, their data, or biological material

Policy information about studies with human participants or human data. See also policy information about sex, gender (identity/presentation), and sexual orientation and race, ethnicity and racism.

Reporting on sex and gender	Not applicable to this study.
Reporting on race, ethnicity, or other socially relevant groupings	Not applicable to this study.
Population characteristics	Not applicable to this study.
Recruitment	Not applicable to this study.
Ethics oversight	Not applicable to this study.

Note that full information on the approval of the study protocol must also be provided in the manuscript.

Field-specific reporting

Please select the one below that is the best fit for your research. If you are not sure, read the appropriate sections before making your selection.

☒ Life sciences ☐ Behavioural & social sciences ☐ Ecological, evolutionary & environmental sciences

For a reference copy of the document with all sections, see nature.com/documents/nr-reporting-summary-flat.pdf

Life sciences study design

All studies must disclose on these points even when the disclosure is negative.

Sample size	Not applicable to this study.
Data exclusions	Not applicable to this study.
Replication	The mass spectrometry studies were conducted using three replicates per treatment condition. All the Western blot (WB) experiments reported in this study were performed at least twice for reproducibility. In an event of a discrepancy, the WB was repeated for a third time. Apoptosis and cell proliferation assays were performed as biological replicates with each replicate containing six technical replicates per treatment.
Randomization	Not applicable to this study.
Blinding	Not applicable to this study.

Reporting for specific materials, systems and methods

We require information from authors about some types of materials, experimental systems and methods used in many studies. Here, indicate whether each material, system or method listed is relevant to your study. If you are not sure if a list item applies to your research, read the appropriate section before selecting a response.

Materials & experimental systems

n/a	Involved in the study	
☐	☒	Antibodies
☐	☒	Eukaryotic cell lines
☒	☐	Palaeontology and archaeology
☒	☐	Animals and other organisms
☒	☐	Clinical data
☒	☐	Dual use research of concern
☒	☐	Plants

Methods

n/a	Involved in the study	
☒	☐	ChIP-seq
☒	☐	Flow cytometry
☒	☐	MRI-based neuroimaging

Antibodies

Antibodies used	phospho-AKTSer473 Rabbit CST #9271 1:1000

Antibodies used

phospho-AKTThr308 Rabbit CST #9275 1:1000
AKT Rabbit CST #9272 1:1000
phospho-AKT substrate Rabbit CST #9614 1:1000
phospho-PKCβ1Ser661 Rabbit Abcam #192184 1:1000
ERα Mouse SantaCruz #8002 1:500
PKCβ1 Rabbit Abcam #136917 1:1000
PKCβ2 Mouse SantaCruz #sc-13149 1:200
cleaved PARP Rabbit CST #5625 1:500
PARP Rabbit CST #9542 1:1000
β-actin Mouse Sigma #A2228 1:20,000
HA-tag Rat Roche #11867423001 1:4,000

Validation

All these antibodies listed are commercially available and purchased from Cell Signaling Technology, Abcam, SantaCruz or Roche. The data sheet for each of the listed antibodies can be obtained from the company website, which contains multiple WB images and citations for antibody validation.

Eukaryotic cell lines

Policy information about cell lines and Sex and Gender in Research

Cell line source(s)

MCF7AC1 cells were a kind gift from the laboratory of Dr. Angela Brodie, University of Maryland, Baltimore, MD.
T47D, T47D-LTED, MDAMB231, BT549 and MDAMB436 were a kind gift from Dr. John R. Hawse, Mayo Clinic, Rochester, MN.
HEK293T cells were a kind gift from Dr. Matthew J. Schellenberg, Mayo Clinic, Rochester, MN.

Authentication

MCF7AC1 and MDAMB231 cell lines were authenticated by short-tandem repeat profiling at Genetica Cell Line Testing.
HEK293T cells were purchased from ThermoFisher.
T47D, BT549 and MDAMB436 cells were a kind gift from the laboratory of Dr. John R. Hawse.

Mycoplasma contamination

All the cell lines reported in this study are mycoplasma negative as confirmed by Polymerase Chain Reaction (PCR) analysis using the Mycoplasma Detection Kit (SouthernBiotech, Catalog # 13100-01) protocol.

Commonly misidentified lines
(See ICLAC register)

None of the commonly misidentified cell line were used in this study.

Plants

Seed stocks

Not applicable to this study.

Novel plant genotypes

Not applicable to this study.

Authentication

Not applicable to this study.
